# Standardization procedure for flow cytometry data harmonization in prospective multicenter studies

**DOI:** 10.1038/s41598-020-68468-3

**Published:** 2020-07-14

**Authors:** Lucas Le Lann, Pierre-Emmanuel Jouve, Marta Alarcón-Riquelme, Christophe Jamin, Jacques-Olivier Pers, PRECISESADS Flow Cytometry Study Group, Montserrat Alvarez, Damiana Alvarez-Errico, Nancy Azevedo, Nuria Barbarroja, Anne Buttgereit, Qingyu Cheng, Carlo Chizzolini, Jonathan Cremer, Aurélie De Groof, Ellen De Langhe, Julie Ducreux, Aleksandra Dufour, Velia Gerl, Maria Hernandez-Fuentes, Laleh Khodadadi, Katja Kniesch, Tianlu Li, Chary Lopez-Pedrera, Zuzanna Makowska, Concepción Marañón, Brian Muchmore, Esmeralda Neves, Bénédicte Rouvière, Quentin Simon, Elena Trombetta, Nieves Varela, Torsten Witte, PRECISESADS Clinical Consortium, Rocío Aguilar-Quesada, Maria Angeles Aguirre-Zamorano, Isabel Almeida, Niklas Baerlecken, Attila Balog, Doreen Belz, Lorenzo Beretta, Ricardo Blanco Alonso, Márta Bocskai, Mariana Brandão, José Luis Callejas Rubio, Ana Campar, Maria-Carmen Castro-Villegas, Ricardo Cervera, Eduardo Collantes, Divi Cornec, Alfonso Corrales Martínez, Magdolna Deák, Valérie Devauchelle-Pensec, Sonja Dulic, Alejandro Escudero-Contreras, Gerard Espinosa, Raquel Faria, Fátima Farinha, María Concepción Fernández Roldán, Tania Gomes Anjos, Miguel A. González-Gay, Falk Hiepe, Nicolas Hunzelmann, Sandrine Jousse-Joulin, Gabriella Kádár, Laszló Kovács, Bernard Lauwerys, Michaela Lehner, Antonio López-Berrio, Rik Lories, António Marinho, Jacqueline Marovac, Pier Luigi Meroni, Blanca Miranda, Immaculada Jiménez Moleón, Héctor Navarro-Linares, Rafaela Ortega-Castro, Norberto Ortego, Enrique Ramón Garrido, Enrique Raya, Raquel Ríos Fernández, Ignasi Rodríguez-Pintó, Alain Saraux, Georg Stummvoll, Carlos Vasconcelos, Michael Zauner

**Affiliations:** 1 INSERM, UMR1227, CHRU Morvan, Lymphocytes B et Autoimmunité, Univ Brest, BP 824, 29609 Brest, France; 2 Altrabio SAS, Lyon, France; 3 GENYO, Centre for Genomics and Oncological Research Pfizer, University of Granada, Andalusian Regional Government, PTS Granada, Granada, Spain; 4 Laboratoire d’Immunologie et Immunothérapie, CHU de Brest, Brest, France; 5 Immunology and Allergy, University Hospital and School of Medicine, Geneva, Switzerland; 6 Chromatin and Disease Group, Bellvitge Biomedical Research Institute (IDIBELL), Barcelona, Spain; 7 Serviço de Imunologia EX-CICAP, Centro Hospitalar e Universitário do Porto, Porto, Portugal; 8 IMIBIC, Reina Sofia Hospital, University of Cordoba, Córdoba, Spain; 9 Bayer AG, Berlin, Germany; 10 Department of Rheumatology and Clinical Immunology, Charité University Hospital, Berlin, Germany; 11 Laboratory of Clinical Immunology, Department of Microbiology and Immunology, KU Leuven, Leuven, Belgium; 12 Pôle de Pathologies Rhumatismales Inflammatoires et Systémiques, Institut de Recherche Expérimentale et Clinique, Université Catholique de Louvain, Brussels, Belgium; 13 Division of Rheumatology, University Hospitals Leuven and Skeletal Biology and Engineering Research Center, KU Leuven, Leuven, Belgium; 14 UCB, Slough, UK; 15 Klinik für Immunologie Und Rheumatologie, Medical University Hannover, Hannover, Germany; 16 Laboratorio di Analisi Chimico Cliniche e Microbiologia - Servizio di Citofluorimetria, Fondazione IRCCS Ca’ Granda Ospedale Maggiore Policlinico di Milano, Milan, Italy; 17 Andalusian Public Health System Biobank, PTS Granada, Granada, Spain; 18 Hospital Universitario Reina Sofía Andaluz de Salud, Córdoba, Spain; 19 Centro Hospitalar do Porto, Porto, Portugal; 20 University of Szeged, Szeged, Hungary; 21 Klinikum Der Universitaet Zu Koeln, Cologne, Germany; 22 Fondazione IRCCS Ca Granda Ospedale Maggiore Policlinico, Milan, Italy; 23 Hospital Universitario Marqués de Valdecilla, Servicio Cántabro de Salud, Santander, Spain; 24 Hospital Universitario San Cecilio, Servicio Andaluz de Salud, Granada, Spain; 25 Hospital Clinic I Provicia, Institut D’Investigacions Biomèdiques August Pi I Sunyer, Barcelona, Spain; 26 Centre Hospitalier Universitaire de Brest, Hopital de La Cavale Blanche, Brest, France; 27 Deutsches Rheuma-Forschungszentrum Berlin, Berlin, Germany; 28 Université Catholique de Louvain, Louvain, Belgium; 29 Medizinische Universitat Wien, Vienna, Austria; 30 Katholieke Universiteit Leuven, Leuven, Belgium; 31 Università Degli Studi di Milano, Milan, Italy; 32 Hospital Regional de Málaga, Servicio Andaluz de Salud, Málaga, Spain

**Keywords:** Software, Data processing

## Abstract

One of the most challenging objective for clinical cytometry in prospective multicenter immunomonitoring trials is to compare frequencies, absolute numbers of leukocyte populations and further the mean fluorescence intensities of cell markers, especially when the data are generated from different instruments. Here, we describe an innovative standardization workflow to compare all data to carry out any large-scale, prospective multicentric flow cytometry analysis whatever the duration, the number or type of instruments required for the realization of such projects.

One of the challenges not yet fully resolved for clinical cytometry^1^ is to compare, in prospective multicenter immunomonitoring trials, the frequencies and absolute values of various leukocyte populations, especially if the data are generated from different instruments and from different companies. The problem is even more difficult when it comes to comparing the expression of the markers^2^ on freshly collected sample to avoid alteration of expression following freezing processes^3^. If the fluorescence variations between different antibody lots remain a major problem, the divergent results can be seen as a result of inappropriate gating strategies between the centers^4^ or because of imperfect fluorescence compensation^5,6^.

Recently, several studies have sought to move this issue forward without being able to meet all the criteria. Some have chosen to use equipment mainly from the same manufacturer^7,8^, others have preferred frozen samples^3,9^ or have resigned themselves to not doing prospective studies^10^, while others have carried out inter-instrument comparability studies but on few samples^11^.

As part of the IMI PRECISESADS study project, a multicenter analysis, using eleven different instruments (Navios, Gallios from Beckman Coulter, Canto II, Fortessa, Verse, Aria from BD Biosciences), prospectively on blood freshly collected from 2,559 individuals over a period of 4 years, was required. The objective was to compare the distribution (frequencies and absolute values) of leukocyte populations and the mean fluorescence intensities (MFIs) of the markers of the studied populations in order to establish a new classification of autoimmune diseases in relation to all the "omic" collection of data. We have preliminarily developed and published a standard operating procedure (SOP) for the standardization of all instruments based on VersaComp Capture beads (Beckman Coulter) for inter-center harmonization. The objective was to establish the settings of each instrument leading to the generation of similar MFIs when acquiring identical samples. A second SOP was also developed using 8 peak beads (Beckman Coulter) for the intra-center daily QC to preserve intra-instrument stability over the project period. Overall, these different SOPs were followed by all centers with the aim of obtaining inter-instrument coefficients of variation (CVs) of less than 5%^12^. The relevance of this approach was demonstrated by an analysis of the same sample of control blood which highlighted a similar sensitivity of all devices in terms of frequencies of populations studied but also in terms of MFI markers studied^12^.

## Harmonization of the instruments

**Figure 1:**
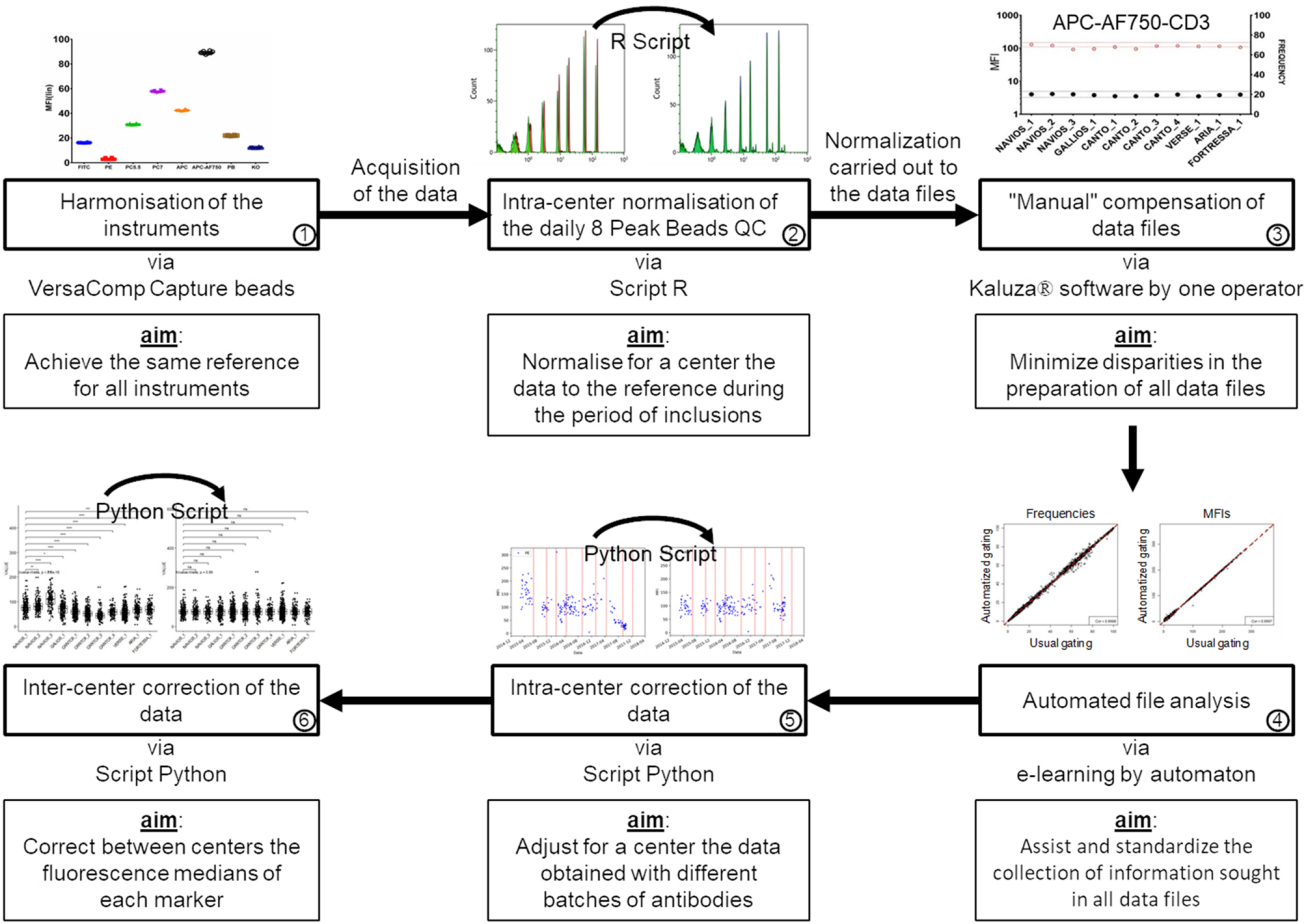
Workflow of the standardization procedure for the harmonization of flow cytometry data in multicentric prospective studies. Flow cytometers are firstly harmonized using VersaComp capture beads to achieve the same reference for all instruments (step 1). For the acquisition of blood samples, 8 peak beads are used as daily quality control. A R script allows the normalization of the data for each instrument to the reference during the period of inclusions (step 2). The compensation of all flow cytometry files are verified and adjusted to minimize disparities in the data file preparation (step 3). Frequencies of the populations of interest and the mean fluorescence intensities of the cell surface markers are automatically collected from all flow cytometry files by automaton having learned the gating strategy through machine learning (step 4). The mean fluorescence intensities of the cell surface markers are corrected by a Python script to adjust the median values and eliminate antibody batches variations in each instrument (step 5). The mean fluorescence intensities are finally corrected by an additional python script to correct the variations of the median values between the instruments (step 6).

The effective harmonization of the instruments through this procedure (Fig. 1, step 1) enables the realization of the large-scale multicentric phenotypic analyzes and assumes the stability of the 11 instruments throughout the duration of the study. Immunomonitoring of the individuals included in the PRECISESADS study was performed using 2 DuraClone antibody panels (Beckman Coulter) dedicated to the analysis of various populations of leukocytes and mononuclear cells in the peripheral blood (Supplementary Table 1). Despite the preliminary precautions taken upstream^12^, the development of a supplementary standardization was necessary to harmonize the flow cytometry data. Different steps have been identified for this procedure and are described in the current manuscript.

## Intra-center normalization

Initially, a first R script was developed to normalize the results over the 4-year period for each single center based on the targets of the initial harmonization in order to correct variations potentially observed in the daily QC (Supplementary Document 1). The different steps are: 1. Loading of the packages flowCore, flowStats,flowViz, ggcyt; 2. Function to revise channel name position and standard names when necessary; 3. Function to extract the MFI of the 8 peak beads files with identification of Beckman Coulter and BD Biosciences files, identification of files containing debris; 4. Function to define the alpha and beta transformation parameters that will be used to normalize the FCS files; 5. Application of the normalization function using a linear regression with the determined alpha and beta parameters and using as a reference the MFI of the 8 peak beads obtained during the initial calibration procedure for the harmonization of the instruments; 6. Verification of the effect in all channels. In order to validate this script, we performed an additional experiment. We voluntarily modified the PMTs of a Navios to provoke a measurement variation of the MFIs of the order of 10 to 15% on the 8 peak beads (Supplementary Figure 1a and Supplementary Table 2). As a result, these changes were made in the MFIs of the membrane markers studied during the acquisition of a blood test sample with DuraClone Panel 1 (Supplementary Figure 1b and Supplementary Table 3). The application of the R script on the LMD files of the 8 peak beads makes its MFI correspond to those obtained without modification of the PMTs, with coefficients of variation lower than 2.5% (Supplementary Figure 1c and Supplementary Table 2). Executed on the LMD files of the blood test sample stained with DuraClone Panel 1, the script also allowed MFIs of the membrane markers corresponding to the MFIs obtained without PMT modification, with coefficients of variation of less than 5% (Supplementary Figure 1d and Supplementary Table 3). Thus, this R script makes it possible to standardize the results generated by the machine (Fig. 1, step 2) and ensures the stability of its data throughout the duration of the project. This standardization R script also enables the comparability of the results between the centers, as demonstrated by the analysis of the immunomonitoring of the same blood control stained with the DuraClone panel 1, carried out after the 4 years. The results show frequency variation coefficients measured between centers ranging from 2.3% for neutrophils to 17.7% for monocytes, and MFI variation coefficients ranging from 10.9% for the CD3 molecule to 30.9% for the CD15 molecule (Supplementary Figure 2), confirming the stability of all instrument mirroring, performed as a prerequisite before starting the inclusions^12^, over the entire study period^12^.

## “Manual” compensation of the flow cytometry data files

The inclusion of the study's 2,559 individuals was done along the way during the 4 years of the PRECISESADS project between December 2014 and December 2018. All FCS and LMD files from the acquisition of samples labeled with DuraClone Panel 1 and DuraClone Panel 2 were standardized with the R script. Then, the standardized file compensation matrices were verified and adjusted when necessary. This step was completed by the same single operator using Kaluza^®^ software (Beckman Coulter) to minimize file preparation variations (Fig. 1, step 3). Values at the beginning (December 2014) and at the end of the inclusions (December 2018) have been compared and found statistically reproducible (not shown).

## Automated gating of the compensated files

**Figure 2:**
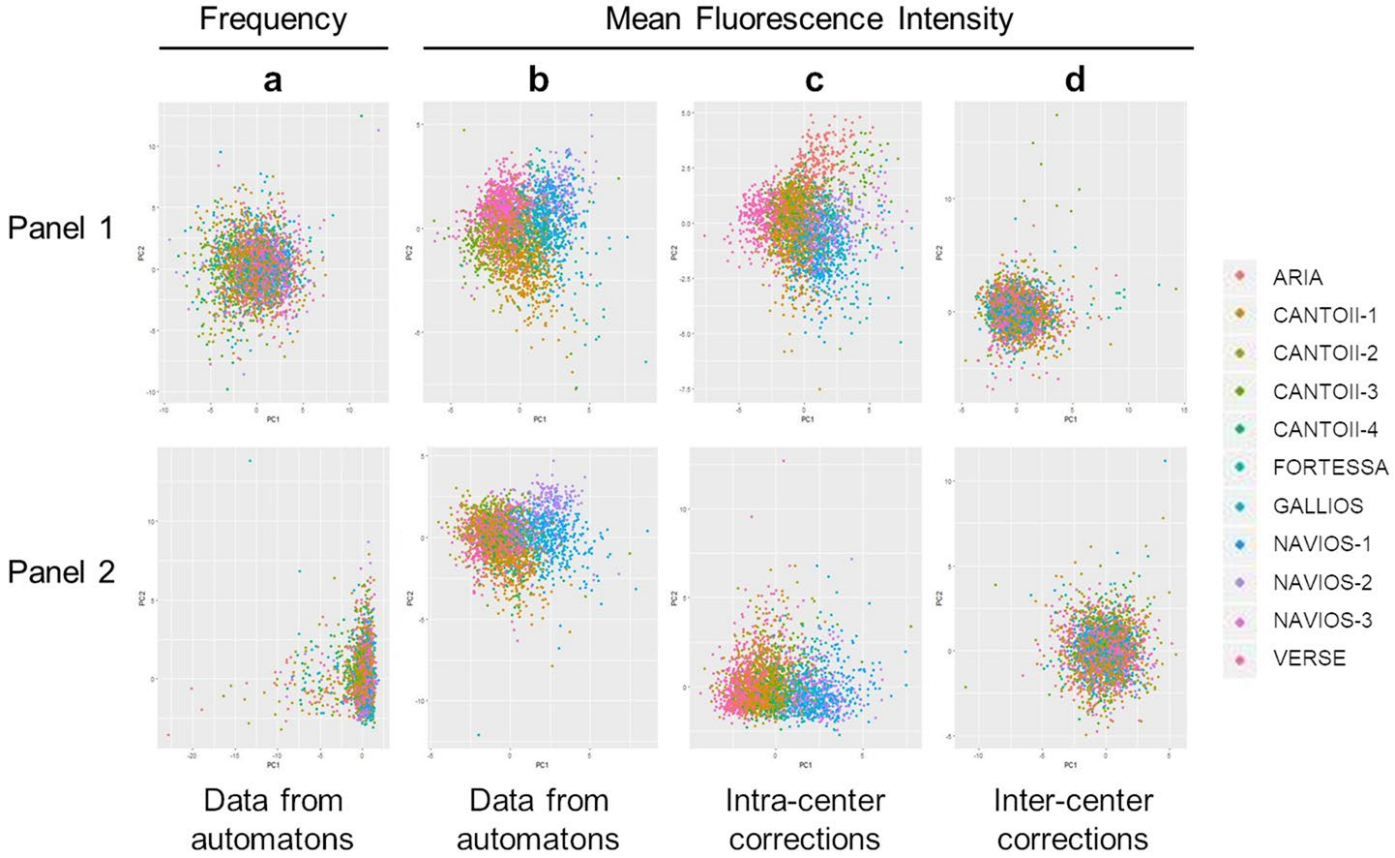
Evolution of principal component analysis during the workflow of the standardization procedure of flow cytometry data in multicentric prospective studies. Peripheral blood of 2,559 individuals was labeled with the dry panel 1 and panel 2 antibody formulations and then analyzed by flow cytometry using 11 different, previously harmonized instruments. The data of each instrument was then standardized by the R script. The frequencies of the leukocyte populations (panel 1) and mononuclear cells (panel 2) (**a**) and the mean fluorescence intensities of the cell surface markers (**b**) were collected from all flow cytometry files by automaton having learned the gating strategy through machine learning and were analyzed by principal component analysis (PCA). The mean fluorescence intensities of the cell surface markers were corrected by a Python script to adjust the median values and eliminate antibody batches variations in each instrument, before being analyzed by PCA (**c**). The mean fluorescence intensities were finally corrected by an additional Python script to eliminate the variations of the median values between the instruments, before being analyzed by PCA (**d**).

Being extremely time-consuming, the “manual” compensation adjustments were followed by a procedure dedicated to the automated (algorithm based) gating of the FCS files, in order to gather information on frequency and absolute number of the cell populations as well as their MFI for the studied markers. Several automated gating algorithms have been able to achieve similar results to the centralized manual gating analysis for public datasets and have shown the advantages of consistency and reproducibility^13^. There are a number of software packages available for automatic analysis by multiple algorithms such as dimensionality reduction^14^, mixture model-based clustering^15^, artificial neural network^16^, density based clustering^17^… Approximately, there are two broad categories of automated flow cytometry data analysis approaches. The supervised methods which are suitable for mimicking manual gating process for predetermined cell populations and the unsupervised strategies which can identify cell populations with minimal user input, and are notably appropriate for biomarker discovery^18,19^. For this current study, in order to address the need of an efficient, consistent and robust analysis of large scale, multi-parameter flow cytometry datasets from multiple immunomonitoring panels in 11 different centers, we have developed, validated and applied robust and reproducible automated pipelines that replicates manual analysis. The automation of gating has been built using a supervised Machine Learning based approach using training datasets gated manually. Some unique approaches have been adopted to leverage data pre-processing, feature engineering, transfer learning and data augmentation to overcome the major challenges encountered in building predictive models for cytometry data automated gating^20^. This automation requires a two steps workflow. A first step customized for each instrument due to potential strong differences in signal for the Forward Scatter and Side Scatter (FS / SS) measures accross cytometers (Supplementary Figure 3a and 3b). A second non instrument specific step, for gating remaining populations of interest (Supplementary Figure 3c and 3d). In order to validate the Machine Learning based algorithms for panel 1 and panel 2 (these algorithms are hereafter name automatons), we carried out additional intermediate evaluations. We compared the results of the automatons with a traditional "manual" analysis performed with the Kaluza^®^ software, on 300 patients distributed throughout the 11 centers. The comparison of the results shows a very good correlation of the data of frequencies, absolute values and the MFIs (Supplementary Figure 4a–c), making it possible to validate the efficiency of the automatons (Fig. 1, step 4). The flow cytometry data (frequencies, absolute values, and MFIs) of the 2,559 individuals included throughout the 4 years of the study are being extracted automatically by the automatons. A principal component analysis (PCA) of these data shows homogeneity of results between centers on frequencies (Fig. 2a) but disparities between centers on MFIs (Fig. 2b). What was not observed in analyses of a small number of samples (reference^12^ and Supplementary Figure 2) has been unveiled with the data from the whole cohort.

## Intra-center correction of the data

We, therefore, sought which parameter(s) could be responsible for these "center effects" despite the initial harmonization of the 11 cytometers and the intra-center normalization of each instrument to circumvent these dispersions.

First, the monoclonal antibodies chosen for immunomonitoring are used in DuraClone (Beckman Coulter) format. This dried antibody formulation provides stability over time, ease of storage, and avoids pipetting errors during sample preparation^21^. However, three different lots were used during the 4 years of the study. The MFI analysis of each panel 1 (Supplementary Figure 5a–d) and panel 2 (Supplementary Figure 5e–g) markers shows variations in batch changes. Therefore, we elaborated a new script under Python to correct the data between batches for each of the 11 instruments including also the variations observed after the 7 calibration procedures^12^ repeated during the 4-year inclusion (see the “Methods” section, and Supplementary Document 2). The different steps are: 1. Importation of the usefull modules (os.path, glob, os, matplotlib.pyplot, matplotlib.cbook, numpy, pandas, csv, re); 2. Extraction of all the data from the FCS files of a patient ID with search of the acquisition dates of the patient ID; 3. Determination of the coefficient for the correction of the batch effect; 4. Computation of the coefficient for each batch and application to the MFI of each channel and saving in a new csv file. Briefly, a coefficient for each batch is calculated by dividing the MFI of all samples of the first batch by the MFI of all samples of each subsequent batch. Then the MFI of each sample is corrected using these coefficients. Based on the alignment of the median values of the different batches with those of the first batch used for each center (Fig. 1, step 5), the variations of the MFIs over time for each of the markers studied disappeared after application of the Python script (Supplementary Figure 5a–g). Nevertheless, despite this correction, the PCA evaluation of the distribution of MFIs between instruments showed a persistent "center effect" (Fig. 2c).

## Inter-center correction of the data

Intrinsic variations in the optical bench of each instrument remained and lead to measurement disparities in MFIs that are dependent on the target of the antibodies used and the associated fluorochromes^8^. Assuming that the number of individuals included is different from one center to another (Supplementary Figure 6a) but that all centers included all the variety of patients (Supplementary Figure 6b) and that the inclusions of the type of individuals were heterogeneous throughout the 4-year period in all centers (Supplementary Figure 6c), the medians of the MFIs on all measures must be equivalent regardless of the center. We therefore developed an additional Python script (Supplementary Document 3) to correct the medians of the MFIs of each marker between all the instruments (Fig. 1, step 6), using the same Navios cytometer as a reference for all other machines throughout the project^12^. The different steps are: 1. Importation of the usefull packages (os.path, glob, os, numpy, re, subprocess); 2. Extraction of the coefficient for the different cytometers used by each center; 3. Computation of the coefficient of each instrument and application of each coefficient to the MFI of all channels saved in a new csv file. Briefly, a coefficient for each channel is calculated by dividing the MFI of all samples of the reference center by the MFI of all samples of all subsequent center. Then the MFI of each sample is corrected using these coefficients. After applying this script, the median values of each marker became insignificantly different regardless of the instrument with which the measurements were made (Supplementary Figure 7a,b). A new PCA showed homogeneous distribution of MFIs for all the markers studied in panel 1 as well as in panel 2 (Fig. 2d). Yet, individual variations specific of each patient are still maintained. As an example, the CD19 MFI variations observed between diseases in all centers persist after application of the script pipeline (Supplementary Figure 7c). Importantly, this indicate that the harmonization procedure, while correcting the instrument differences, preserve the biological differences between individuals.

Finally, the implementation of the workflow described in Fig. 1 made it possible to compare all the data of frequencies, absolute values and MFIs resulting from the data generated by the immunomonitoring of 2,559 individuals included over a period of 4 years by 11 different flow cytometers. Biostatistical analyzes including these flow cytometry data with the set of "omic" data collected as part of the PRECISESADS project are now possible. More broadly, with all the validity required to follow the elaborated standardization procedures, this new workflow opens the possibility of carrying out any large-scale, prospective multicentric flow cytometry analysis whatever the duration, number and type of instruments required for the realization of such projects.

## Methods

### Participant recruitment

The PRECISESADS study is a European multi center, non-randomized, and observational clinical study with recruitment performed between December 2014 and December 2018 at 19 institutions in 9 countries (Austria, Belgium, France, Germany, Hungary, Italy, Portugal, Spain and Switzerland). The study had two types of patient recruitment that included a cross-sectional study and an inception prospective study with patients followed at two time points, registered with number NCT02890121 and NCT02890134 in ClinicalTrials.gov, respectively. The target number of included patients affected by systemic autoimmune diseases was 2005 (around 400 by disease or group of diseases: rheumatoid arthritis, scleroderma or systemic sclerosis, primary Sjögren’s syndrome, systemic lupus erythematosus, and primary antiphospholipid syndrome, mixed connective tissue disease and undifferentiated connective tissue disease) and 554 healthy controls. An ethical protocol was prepared, reached consensus across all partners, academic and industrial, translated into all participant’s languages and approved by each of the local ethical committees of the clinical recruitment centers, and all experimental protocols were approved by each of the local committees:

- Referral Center for Systemic Autoimmune Diseases, Fondazione IRCCS Ca’ Granda Ospedale Maggiore Policlinico di Milano, Comitato Etico Italy.
- Centre Hospitalier Universitaire de Brest, Hospital de la Cavale Blanche, Avenue Tanguy Prigent 29,609, Brest, France. Comite de Protection des Personnes Ouest VI.
- Pôle de pathologies rhumatismales systémiques et inflammatoires, Institut de Recherche Expérimentale et Clinique, Université catholique de Louvain, Brussels, Belgium. Comité d`Èthique Hospitalo-Facultaire.
- Centro Hospitalar do Porto, Portugal. Comissao de ética para a Saude – CES do CHP.
- Servicio Cantabro de Salud, Hospital Universitario Marqués de Valdecilla, Santander, Spain. Comite ético de investigacion clinical de Cantabria. IDIVAL.
- Hospital Clinic I Provicia, Institut d’Investigacions Biomèdiques August Pi i Sunyer, Barcelona, Spain. Comité Ética de Investigación Clínica del Hospital Clínic de Barcelona. HOSPITAL CLíNIC DE BARCELONA.
- Katholieke Universiteit Leuven, Belgium. Commissie Medische Ethiek UZ KU Leuven /Onderzoek.
- Klinikum der Universitaet zu Koeln, Cologne, Germany. Geschaftsstelle Ethikkommission
- Medizinische Hochschule Hannover, Germany. Ethikkommission.
- Medical University Vienna, Vienna, Austria. Ethik Kommission. Borschkegasse.
- Servicio Andaluz de Salud, Hospital Universitario Reina Sofía Córdoba, Spain. Comité de Ética e la Investigación de Centro de Granada (CEI – Granada).
- Servicio Andaluz de Salud, Complejo hospitalario Universitario de Granada (Hospital Universitario San Cecilio), Spain. Comité de Ética e la Investigación de Centro de Granada (CEI – Granada).
- Servicio Andaluz de Salud, Complejo hospitalario Universitario de Granada (Hospital Virgen de las Nieves), Spain. Comité de Ética e la Investigación de Centro de Granada (CEI – Granada).
- Servicio Andaluz de Salud, Hospital Regional Universitario de Málaga, Spain. Comité de Ética e la Investigación de Centro de Granada (CEI – Granada).
- Università degli studi di Milano, Milan, Italy. Policlinico di Milano, Comitato Etico Italy.
- Hospitaux Universitaires de Genève, Switzerland. DEAS –Commission Cantonale d`´ethique de la recherche Hopitaux universitaires de Geneve.
- University of Szeged, Szeged, Hungary. Csongrad Megyei Kormanyhivatal.
- Charite, Berlin, Germany. Ethikkommission.
- Andalusian Public Health System Biobank, Granada, Spain.
- Comité de Ética e la Investigación de Centro de Granada (CEI – Granada).

All patients recruited to the study were aged 18 years or older and signed an informed consent form, and all methods were carried out in accordance with relevant guidelines and regulations. The study adhered to the standards set by International Conference on Harmonization and Good Clinical Practice, and to the ethical principles that have their origin in the Declaration of Helsinki (2013). The protection of the confidentiality of records that could identify the included individuals is ensured as defined by the EU Directive 2001/20/EC and the applicable national and international requirements relating to data protection in each participating country.

The immunophenotyping of the individuals were performed by flow cytometry using 2 DuraClone antibody panels (Beckman Coulter) dedicated to the analysis of various populations of leukocytes and mononuclear cells in the peripheral blood of individuals (Supplementary Table 1). Absolute numbers using Flow Count fluorospheres (Beckman Coulter), frequencies of the different cell populations and MFIs of the cell surface markers were collected: CD3+CD19- T cells, CD3+CD4+CD8- T cells, CD3+CD4-CD8+ T cells, CD3-CD19+ B cells, CD3-CD56+ NK cells, CD3-CD56highCD16low NK cells, CD3-CD56lowCD16high NK cells CD3+ CD56+ NK-like T cells, CD4lowCD14+CD16- classic monocytes, CD4lowCD14+ CD16+ intermediate monocytes, CD4lowCD14-CD16+ non-classic monocytes, CD15+CD16low eosinophils, and CD15+CD16high neutrophils with panel 1 (Supplementary Figure 3c); HLA-DR-CD123+ basophils, HLA-DR+Lin-CD123+ CD11c- pDC, HLA-DR+ Lin-CD123-CD11c+ mDC, HLA-DR+ Lin-CD123-CD11c+ CD1c+CD141- mDC1 and HLA-DR+Lin-CD123-CD11c+CD1c-CD141+ mDC2 with panel 2 (Supplementary Figure 3d).

Furthermore, to reduce the variability that may occur during the sample preparations, all centers received the same SOP explaining how to prepare the lysis buffer, to handle the required volume of blood samples and rehydrate the antibodies in the DuraClone tubes, to add FlowCount fluorospheres (Beckman Coulter) for the absolute number calculation and finally how to prepare the instruments on a daily basis with the acquisition of Rainbow 8-peak beads (Beckman Coulter). These standardize procedure have been followed by all users in all centers to minimize technical bias.

Flow cytometry acquisition was managed at each center after a multi-center harmonization of flow cytometers to ensure mirroring of all instruments, thereby allowing subsequent integration of all the data obtained across the different sites and instruments, as previously described^12^. Briefly, one flow cytometer (NAVIOS-1) was chosen as reference instrument and fixed the mean fluorescence intensities (MFIs) of 8 different fluorochrome conjugated antibodies using VersaComp Ab capture beads (Beckman Coulter). The ten other instruments adjusted their own PMT voltages to reach the same MFIs. These values constituted their initial reference settings. Furthermore, to evaluate the fluctuation of the instruments overtime and to minimize deviation, this standardization procedure was repeated every 3 to 6 months. If required, PMT voltages were adjusted to maintain identical MFI values of the target fluorescence intensities. In that case, the PMT values were set in the 2 multiparameter panels serving as new intra-instrument reference assessments for the inclusion of future individual samples. Subsequently to the standardization of all instruments, each center used Rainbow 8-peak beads (Beckman Coulter) on a daily basis to follow the stability of their instrument overtime. The deviation of the MFI values of the peaks of every fluorochrome must be < 5% compared to the internal reference. In cases where instrument performance failed, while cleaning, de-gassing flow cell and laser delay were verified, PMT values were modified to adjust the position of the fluorescence peaks in their initial positions. Revised PMTs were then reported in the 2 panels of the immunophenotyping. Compensation matrices that could be impacted were adjusted later during the analysis procedure.

Moreover, to facilitate the elaboration of the scripts able to read LMD files generated by Beckman Coulter instruments as well as FCS files generated by BD Biosciences instruments, all centers received SOPs before starting the inclusions of samples to generate consistent instrument files. These SOPs were dedicated to the preparation of the flow cytometers (namely the protocols for the Beckman Coulter instruments and the sheets for the BD instruments), including uniform specific name of the different parameters (forward scatter and side scatter settings, fluorochrome designation), identical stop count or time of acquisition, and uniform labelling of the LMD and FCS files. Despite all these precautions, errors have been detected for some files. The script codes have thus been modified and implemented to detect and correct these errors in order to avoid the exclusion of the corresponding samples from the overall analyses (Supplementary Documents).

## Supplementary information

Supplementary Legends.

Supplementary Tables.

Supplementary Documents.

Supplementary Figure 1.

Supplementary Figure 2.

Supplementary Figure 3.

Supplementary Figure 4.

Supplementary Figure 5.

Supplementary Figure 6.

Supplementary Figure 7.

## References

[1] Pedreira CE (2013). Overview of clinical flow cytometry data analysis: recent advances and future challenges. Trends Biotechnol..

[2] Bottcher S (2017). Lot-to-lot stability of antibody reagents for flow cytometry. J. Immunol. Methods..

[3] Ivison S (2018). A standardized immune phenotyping and automated data analysis platform for multicenter biomarker studies. JCI Insight..

[4] Finak G (2016). Standardizing flow cytometry immunophenotyping analysis from the human immunophenotyping consortium. Sci. Rep..

[5] Kalina T (2015). Quality assessment program for EuroFlow protocols: summary results of four-year (2010–2013) quality assurance rounds. Cytometry A.

[6] Kalina T (2018). Frequent issues and lessons learned from EuroFlow QA. J. Immunol. Methods.

[7] Streitz M (2013). Standardization of whole blood immune phenotype monitoring for clinical trials: panels and methods from the ONE study. Transplant. Res..

[8] Kalina T (2012). EuroFlow standardization of flow cytometer instrument settings and immunophenotyping protocols. Leukemia.

[9] Veluchamy JP (2017). Standardized and flexible eight colour flow cytometry panels harmonized between different laboratories to study human NK cell phenotype and function. Sci. Rep..

[10] Solly F (2013). Comparable flow cytometry data can be obtained with two types of instruments, Canto II, and Navios. A GEIL study. Cytometry A.

[11] Glier H (2017). Standardization of 8-color flow cytometry across different flow cytometer instruments: a feasibility study in clinical laboratories in Switzerland. J. Immunol. Methods..

[12] Jamin C (2016). Multi-center harmonization of flow cytometers in the context of the European “PRECISESADS” project. Autoimmun. Rev..

[13] Aghaeepour N (2013). Critical assessment of automated flow cytometry data analysis techniques. Nat. Methods.

[14] Amir AD (2013). viSNE enables visualization of high dimensional single-cell data and reveals phenotypic heterogeneity of leukemia. Nat. Biotechnol..

[15] Sorensen T (2015). immunoClust—an automated analysis pipeline for the identification of immunophenotypic signatures in highdimensional cytometric datasets. Cytometry A.

[16] Van Gassen S (2015). FlowSOM: using self-organizing maps for visualization and interpretation of cytometry data. Cytometry A.

[17] Qian Y (2010). Elucidation of seventeen human peripheral blood B-cell subsets and quantification of the tetanus response using a density-based method for the automated identification of cell populations in multidimensional flow cytometry data. Cytometry B Clin. Cytom..

[18] Mair F (2016). The end of gating? An introduction to automated analysis of high dimensional cytometry data. Eur. J. Immunol..

[19] Conrad VK (2019). Implementation and validation of an automated flow cytometry analysis pipeline for human immune profiling. Cytometry A.

[20] Lee H (2019). High-throughput analysis of clinical flow cytometry data by automated gating. Bioinform Biol Insights.

[21] Hedley BD, Keeney M, Popma J, Chin-Yee I (2015). Novel lymphocyte screening tube using dried monoclonal antibody reagents. Cytometry B Clin. Cytom..

