# Supplementary material for: Standardization procedure for flow cytometry data harmonization in prospective multicenter studies
Source: Sci Rep. 2020 Jul 14;10:11567. doi: 10.1038/s41598-020-68468-3 (PMC7360585; doi:10.1038/s41598-020-68468-3)
Supplement: Supplementary file 1 — Supplementary Legends. [file 41598_2020_68468_MOESM1_ESM.pdf]

Standardization procedure for flow cytometry data harmonization in prospective multicenter studies

Lucas Le Lann[1], PRECISESADS Flow Cytometry Study Group[1] and PRECISESADS Clinical Consortium[1], Pierre-Emmanuel Jouve[2], Marta Alarcón-Riquelme[3], Christophe Jamin[1,4], Jacques-Olivier Pers[1]

Supplementary Table 1. Composition of the two panels of the dry formulation of antibodies (DuraClone). Fluorochromes indicated in the line title are classical fluorochromes from Beckman Coulter. The DuraClone panel compositions (Beckman Coulter) indicate the target molecule and common aliases, with the antibody clones mentioned in the brackets. FITC: fluorescein isothiocyanate; PE: phycoerythrin; PC5.5: PE-cyanin 5.5; PC7: PE-cyanin 7; APC: allophycocyanin; APC-AF750: APC-alexa fluor 750; PB: pacific blue; KRO: krome orange.

Supplementary Table 2. Evaluation of the normalization using a script R with the 8 peak beads fluorescence. The mean fluorescence intensity of the most intense peak from the 8 peak beads is measured before (Reference) and after (Shift) the modification of the PMT values of a Navios flow cytometer. The variation with the reference after the shift is indicated. The application of the R script on the LMD files after the shift of the PMTs restores the initial values of the mean fluorescence intensity with abolition of the variations. PMT: photomultiplying tube; FITC: fluorescein isothiocyanate; PE: phycoerythrin; PC5.5: PE-cyanin 5.5; PC7: PE-cyanin 7; APC: allophycocyanin; APC-AF750: APC-alexa fluor 750; PB: pacific blue; KRO: krome orange. Mean±SD of 3 experiments.

Supplementary Table 3. Evaluation of the normalization of the cell surface fluorescence using a R script. The peripheral blood cells from a control individual were labeled with the dry panel 1 DuraClone formulation. The mean fluorescence intensity of the positive cells for each marker was measured before (Reference) and after (Shift) the modification of the PMT values of a Navios flow cytometer. The variation with the reference after the shift is indicated. The application of the R script on the LMD files after the shift of the PMTs restores the initial values of the mean fluorescence intensity with abolition of the variations. PMT: photomultiplying tube; FITC: fluorescein isothiocyanate; PE: phycoerythrin; PC5.5: PE-cyanin 5.5; PC7: PE-cyanin 7; APC: allophycocyanin; APC-AF750: APC-alexa fluor 750; PB: pacific blue; KRO: krome orange. Mean±SD of 3 experiments.

Supplementary Document 1. R script for the data normalization of a single flow cytometer. R script runs for the intra-center normalization of the daily QC 8 peak beads of a flow cytometer on its 8 peak beads reference obtained after the harmonization of all the instruments. The coefficients of normalization are then carried out to the data files from peripheral blood stained with panel 1 and panel 2. Underlined and bold headers indicate the different steps of the script.

Supplementary Document 2. Python script for the data correction of a single flow cytometer. Python script runs for the intra-center correction of the mean fluorescence intensities of the markers analyzed from panel 1 and panel 2 stainings generated by a single flow cytometer to adjust the variations induced by the different batches of fluorochrome-associated antibodies during the duration of the study to the first batch used. Underlined and bold headers indicate the different steps of the script.

Supplementary Document 3. Python script for the data correction between all flow cytometers. Python script runs for the inter-center correction of the mean fluorescence intensities of the markers analyzed from panel 1 and panel 2 stainings generated by all flow cytometers to adjust the variations of the median values of all individuals obtained in one center to the median values of a reference center. Underlined and bold headers indicate the different steps of the script.

Supplementary Figure 1. Validation of the R script for the data normalization of a single flow cytometer. Fluorescence profile of the 8 peaks beads before (Fluorescence Reference) and after (Fluorescence with 15% deviation) modification of the PMTs of each of the 8 channels of a Navios flow cytometer **(a)**. The peripheral blood of a control was labeled with the dry panel 1 antibody formulation and analyzed with the Navios flow cytometer before and after modification of the PMTs of each of the channels **(b)**. The LMD files of the 8 peaks beads **(c)** and the blood sample **(d)** were standardized by the R script (15% deviated fluorescence normalization) and the fluorescence profiles

compared to their respective references. FITC: fluorescein isothiocyanate; PE: phycoerythrin; PC5.5: PE-cyanin 5.5; PC7: PE-cyanin 7; APC: allophycocyanin; APC-AF750: APC-alexa fluor 750; PB: pacific blue; KRO: krome orange.

Supplementary Figure 2. Checking the stability of the harmonization of the eleven flow cytometers. In all centers, the same batch of control blood was labeled with the dry panel 1 antibody formulation and analyzed by each of the eleven flow cytometers. The frequencies of the population targeted by the markers and the mean fluorescence intensities of the surface markers were obtained by an analysis with the Kaluza® software. The dotted lines indicate the threshold of 5% of variation compared to the Navios-1 reference flow cytometer. FITC: fluorescein isothiocyanate; PE: phycoerythrin; PC5.5: PE-cyanin 5.5; PC7: PE-cyanin 7; APC: allophycocyanin; APC-AF750: APC-alexa fluor 750; PB: pacific blue; KRO: krome orange.

Supplementary Figure 3. Gating strategies of the automatons. For all instruments, the data from the flow cytometry files are analyzed with a similar strategy by one automaton for the panel 1 and another automaton for the panel 2 **(a)**, and then specifically for each instrument from the gate [S4] to account for the variability of FSC and SSC signals **(b)**. The desired cell populations are identified by gating strategies identical for all instruments for panel 1 **(c)** and panel 2 **(d)** stainings.

Supplementary Figure 4. Comparisons of results obtained by traditional analysis and automated analysis. The peripheral blood of 300 individuals was labeled with the dry panel 1 and panel 2 antibody formulations and then analyzed by flow cytometry using 9 different, previously harmonized instruments. The data of each instrument was then standardized by an R script. The frequencies of the cell populations **(a)**, the absolute values **(b)** and the mean fluorescence intensities of the cell surface markers **(c)** were obtained by a simple manual analysis using Kaluza® software, and by automaton having learned the gating strategy through e-learning and compared.

Supplementary Figure 5. Intra-center correction of the fluorescence intensities of all the markers according to the batches formulation used. The peripheral blood of 2,559 individuals was labeled with the dry panel 1 **(a-d)** and panel 2 **(e-g)** antibody formulations and analyzed by flow cytometry using 11 different, previously harmonized instruments. The data of each instrument was then standardized by an R script. The mean fluorescence intensities of the cell surface markers were collected from all flow cytometry files by automaton having learned the gating strategy through e-learning (Before) and then treated with a Python script to adjust the median values (After) for the correction of the variations due to the batches of antibodies. The red dashed lines indicate the date of introduction of a new lot of antibodies and the black dashed lines indicate the common recurrent calibration procedures performed overtime. SLE: Systemic Lupus Erythematosus; SSc: Systemic Sclerosis; RA: Rheumatoid Arthritis; SjS: Sjörgren's Syndrome; UNDIFF: Undifferentiated Connective Tissue Disease.

Supplementary Figure 6. Evaluation of the disparity of the inclusions in all centers. For each instrument, the number **(a)**, the frequencies **(b)**, and the inclusion of the individuals over time **(c)** are shown. SLE: Systemic Lupus Erythematosus; SSc: Systemic Sclerosis; RA: Rheumatoid Arthritis; SjS: Sjörgren's Syndrome; UNDIFF: Undifferentiated Connective Tissue Disease.

Supplementary Figure 7. Comparisons between instruments of the mean fluorescence intensities after inter-center correction of the results. The peripheral blood of 2,559 individuals was labeled with the dry panel 1 **(a)** and panel 2 **(b)** antibody formulations, and analyzed by flow cytometry using 11 different, previously harmonized instruments. The data of each instrument was then standardized by an R script. The mean fluorescence intensities of the cell surface markers from leukocyte populations **(a)** and from mononuclear cells **(b)** were collected by an automaton having learned the gating strategy through e-learning, then corrected by a Python script to eliminate antibody batches variations in each instrument and finally corrected by an additional Python script to correct the fluorescence medians between instruments. The results collected by the 11 instruments

were compared with each other before and after the additional Python script. **(c)** The CD19 MFI was extracted before and after corrections with the workflow of the scripts to verify the persistence of the individual variations. *p<0.05; **p<0.01; ***p<0.001; ns: not significant. SLE: Systemic Lupus Erythematosus; SSc: Systemic Sclerosis; RA: Rheumatoid Arthritis; SjS: Sjörgren’s Syndrome; UNDIFF: Undifferentiated Connective Tissue Disease.
