# Supplementary material for: Standardization procedure for flow cytometry data harmonization in prospective multicenter studies
Source: Sci Rep. 2020 Jul 14;10:11567. doi: 10.1038/s41598-020-68468-3 (PMC7360585; doi:10.1038/s41598-020-68468-3)
Supplement: Supplementary file 2 — Supplementary Tables. [file 41598_2020_68468_MOESM2_ESM.pdf]

# Standardization procedure for flow cytometry data harmonization in prospective multicenter studies

Lucas Le Lann[1], PRECISESADS Flow Cytometry Study Group[1] and PRECISESADS Clinical Consortium[1], Pierre-Emmanuel Jouve[2], Marta Alarcón-Riquelme[3], Christophe Jamin[1,4], Jacques-Olivier Pers[1]

## Supplementary Tables

**Supplementary Table 1.** Composition of the two panels of the dry formulation of antibodies (DuraClone).

| | **FITC** | **PE** | **PC5.5** | **PC7** | **APC** | **APC-AF750** | **PB** | **KRO** |
|---|---|---|---|---|---|---|---|---|
| **Panel 1 (clone)** | CD16 (3G8) | CD15 (80H5) | CD56 (N901) | CD14 (RMO52) | CD19 (J4.119) | CD3 (UCHT1) | CD4 (13B8.2) | CD8 (B9.11) |
| **Panel 2 (clone)** | CD1c (L161) | Lin (various) | CD141 (M80) | CD11c (BU15) | CD123 (SSDCLY1 07D2) | DRAQ7 (drop in) | HLA DR (IMMU357) | |

Fluorochromes indicated in the line title are classical fluorochromes from Beckman Coulter. The DuraClone panel compositions (Beckman Coulter) indicate the target molecule and common aliases, with the antibody clones mentioned in the brackets. FITC: fluorescein isothiocyanate; PE: phycoerythrin; PC5.5: PE-cyanin 5.5; PC7: PE-cyanin 7; APC: allophycocyanin; APC-AF750: APC-alexa fluor 750; PB: pacific blue; KRO: krome orange.

**Supplementary Table 2.** Evaluation of the normalization using a script R with the 8 peak beads fluorescence.

| 8 Peak beads | Reference | | Shift | | | Script for normalization | |
|---|---|---|---|---|---|---|---|
| **Channel** | **PMT** | **Intensity** | **PMT** | **Intensity** | **Variation (% with Reference)** | **Intensity** | **Variation (% with Reference)** |
| FITC | 421 | 129.2±0.1 | 429 | 146.8±0.9 | 13.6±0.8 | 127.3±0.03 | -1.4±0.03 |
| PE | 426 | 211.9±0.1 | 435 | 243.5±1.4 | 14.9±0.7 | 210.6±0.04 | -0.6±0.05 |
| PC5.5 | 466 | 89.4±0.1 | 476 | 102.9±0.6 | 15.1±0.6 | 89.8±0.01 | 0.4±0.07 |
| PC7 | 556 | 51.6±0.1 | 565 | 57.7±0.5 | 11.8±0.7 | 50.4±0.01 | -2.3±0.22 |
| APC | 515 | 808.3±0.7 | 525 | 926.7±5.8 | 14.6±0.6 | 800.8±0.26 | -0.9±0.06 |
| APC-AF750 | 634 | 240.2±0.4 | 645 | 272.4±4.0 | 13.4±1.8 | 237.5±0.30 | -1.1±0.30 |
| PB | 412 | 541.8±1.2 | 421 | 623.6±4.3 | 15.1±1.0 | 532.1±0.06 | -1.8±0.20 |
| KRO | 372 | 487.5±0.8 | 380 | 562.6±3.4 | 15.4±0.9 | 481.7±0.02 | -1.2±0.17 |

The mean fluorescence intensity of the most intense peak from the 8 peak beads is measured before (Reference) and after (Shift) the modification of the PMT values of a Navios flow cytometer. The variation with the reference after the shift is indicated. The application of the R script on the LMD files after the shift of the PMTs restores the initial values of the mean fluorescence intensity with abolition of the variations. PMT: photomultiplying tube; FITC: fluorescein isothiocyanate; PE: phycoerythrin; PC5.5: PE-cyanin 5.5; PC7: PE-cyanin 7; APC: allophycocyanin; APC-AF750: APC-alexa fluor 750; PB: pacific blue; KRO: krome orange. Mean±SD of 3 experiments.

**Supplementary Table 3.** Evaluation of the normalization of the cell surface fluorescence using a R script.

| Antibody | | Reference | | Shift | | | Script for normalization | |
|---|---|---|---|---|---|---|---|---|
| **Channel** | **Target** | **PMT** | **Intensity** | **PMT** | **Intensity** | **Variation (% with Reference)** | **Intensity** | **Variation (% with Reference)** |
| FITC | CD16 | 421 | 70.3±0.4 | 429 | 84.3±0.2 | 20.0±0.6 | 73.6±0.10 | 4.7±0.52 |
| PE | CD15 | 426 | 66.7±0.2 | 435 | 75.5±0.4 | 13.2±0.4 | 65.7±0.15 | -1.6±0.09 |
| PC5.5 | CD56 | 466 | 8.4±0.3 | 476 | 10.2±0.1 | 21.0±4.2 | 8.6±0.42 | 1.6±2.72 |
| PC7 | CD14 | 556 | 217.5±0.3 | 565 | 250.8±0.2 | 15.3±0.1 | 221.6±0.26 | 1.9±0.23 |
| APC | CD19 | 515 | 30.3±0.2 | 525 | 32.6±0.4 | 7.6±1.5 | 28.8±0.31 | -4.8±0.41 |
| APC-AF750 | CD3 | 634 | 106.4±0.4 | 645 | 121.6±0.3 | 14.3±0.7 | 107.8±0.15 | 1.3±0.54 |
| PB | CD4 | 412 | 14.6±0.2 | 421 | 16.5±0.4 | 12.8±3.6 | 14.3±0.21 | -2.5±2.06 |
| KRO | CD8 | 372 | 20.2±0.2 | 380 | 22.4±0.3 | 9.5±1.3 | 19.4±0.31 | -4.0±0.88 |

The peripheral blood cells from a control individual were labeled with the dry panel 1 DuraClone formulation. The mean fluorescence intensity of the positive cells for each marker was measured before (Reference) and after (Shift) the modification of the PMT values of a Navios flow cytometer. The variation with the reference after the shift is indicated. The application of the R script on the LMD files after the shift of the PMTs restores the initial values of the mean fluorescence intensity with abolition of the variations. PMT: photomultiplying tube; FITC: fluorescein isothiocyanate; PE: phycoerythrin; PC5.5: PE-cyanin 5.5; PC7: PE-cyanin 7; APC: allophycocyanin; APC-AF750: APC-alexa fluor 750; PB: pacific blue; KRO: krome orange. Mean±SD of 3 experiments.
