# Supplementary material for: Standardization procedure for flow cytometry data harmonization in prospective multicenter studies
Source: Sci Rep. 2020 Jul 14;10:11567. doi: 10.1038/s41598-020-68468-3 (PMC7360585; doi:10.1038/s41598-020-68468-3)
Supplement: Supplementary file 3 — Supplementary Documents. [file 41598_2020_68468_MOESM3_ESM.pdf]

Standardization procedure for flow cytometry data harmonization in prospective multicenter studies

Lucas Le Lann[1], PRECISESADS Flow Cytometry Study Group[1] and PRECISESADS Clinical Consortium[1], Pierre-Emmanuel Jouve[2], Marta Alarcón-Riquelme[3], Christophe Jamin[1,4], Jacques-Olivier Pers[1]

Supplementary Documents

Supplementary Document 1. R script for the data normalization of a single flow cytometer.

```
## loading packages ##

suppressPackageStartupMessages(
{
  library(flowCore)
  library(flowStats)
  library(flowViz)
  library(ggcyto)
})
require(Ckmeans.1d.dp)


#### BEGIN FUNCTION ####


# Function to put the flow cytometry data to the scale used by Kaluza software from Beckman Coulter.
KalTransform <- function(transformId, a){
  t = new("transform", .Data = function(x) (x/a))
  t@transformationId = transformId
  t
}


#Function that will extract the MFI of the 8 peak beads files.
border <-function(fcs,eight_peaks){


        name_file<-basename(eight_peaks)
```

median_peak <- vector("list", 8)

#Test if the file is from Beckman Coulter or from Beckton Dickinson.

test_lmd <- grepl(".LMD",eight_peaks , fixed=TRUE)

if (test_lmd){

KalT <- KalTransform(transformId="KalT",a=1024)

mult=1024

} else{

KalT <- KalTransform(transformId="KalT",a=256)

mult=256

}

#Transform the data from linear to non-linear (inverse hyperbolic sine).

translist <- transformList(fluo.channels, KalT)

after_tmp<- transform(fcs, translist)

arcTrans <- arcsinhTransform(transformationId="defaultArcsinhTransform", a=0, b=1, c=0)

translist <- transformList(fluo.channels,arcTrans)

after<- transform(after_tmp, translist)

#Test if the center is IDIBELL to have an additional gate to remove debris.

#Use lymphGate to remove unintended events

test_IDIBELL <- grepl("IDIBELL",eight_peaks , fixed=TRUE)

if (test_lmd){

```
			if (test_IDIBELL){
				rgate <- rectangleGate(filterId="RG","FS-A"=c(100, 1000000),"SS-
A"=c(100000, 1000000))
				xyplot(`SS-A`~`FS-A`, after, filter=rgate)
				fres <- filter(after, rgate)
				fcs_split<- split(after, fres)
				beads <- fcs_split[1]$'RG+'
				lg <- flowStats::lymphGate(beads, channels = c("SS-A", "FS-
A"),filterID=rgate1,scale= 3.0, plot = F)
				xyplot(`SS-A`~`FS-A`,beads, filter=lg$n2gate)

			} else{
				lg <- flowStats::lymphGate(after, channels = c("SS-A", "FS-
A"),scale= 3.0, plot = F)
				xyplot(`SS-A`~`FS-A`, after, filter=lg$n2gate)
			}
		} else{
		lg <- flowStats::lymphGate(after, channels = c("SSC-A", "FSC-A"),scale= 5.0, plot = F)
		xyplot(`SSC-A`~`FSC-A`, after, filter=lg$n2gate)
		}

		i=1
		# Define the number of cluster for each channel and special one for UBO
		for (channel in fluo.channels){
			nb_clust <- function(type) {
```

```
		switch(type,"FITC.A"=8,
				"PE.A"=8,
				"PC5.5.A"=6,
				"PC7.A"=6,
				"APC.A"=8,
				"APC.AF750.A"=8,
				"PB.A"=8,
				"KO.A"=8)
	}
	k <- nb_clust(channel)

	if (grepl( "UBO",name_file) & (channel == "APC.AF750.A")){
		k <- 9
	}
```

#Use of Ckmedian.1d.dp to get the number of cluster in 1 dimension, the channel.

```
	result <- Ckmedian.1d.dp(lg$x@exprs[,channel], k)
	m<-result$center

	#Remove some particularities due to different sensibilities in the cytometers
	#And add the channel to the median matrix.
	if (grepl( "UCL",name_file) & (channel == "KO.A")){
		median_peak[[i]]<-(sinh(m)*mult)[-8]
	} else if (grepl("KUL",name_file) & (channel == "APC.A")){
```

```
                median_peak[[i]]<-(sinh(m)*mult)[-8]
            }else {
                median_peak[[i]]<-sinh(m)*mult
            }
            names(median_peak)<-fluo.channels
            i=i+1

            #plot for the pdf
            plot(density(lg$x@exprs[,channel],bw = 0.001),main = channel)
            points(x=result$center,y = rep(3:4,length.out=k),col=1:k,pch=0,cex=1)

        }
        dev.off()
        return(median_peak)
}
```

#### END FUNCTION ####

```
args <- commandArgs(trailingOnly = TRUE)

PEAKSDataPath<-args[1]
REFDataPath<-args[2]
flowDataPath<-args[3]

#PEAKSDataPath<- 8 peak beads file
```

#REFDataPath<- Reference for the 8 peak bead file

#flowDataPath<- Path to the associated panel

```
fluo.channels<-
c("FITC.A","PE.A","PC5.5.A","PC7.A","APC.A","APC.AF750.A","PB.A","KO.A")

PB_REF <- flowCore::read.FCS(file.path(REFDataPath),dataset=2)
```

#Deal with particularities in the channel name position and renaming to standard names

```
test_nb_colname <- grepl("MHH",REFDataPath , fixed=TRUE)

test_parameters1<-grepl("DRFZ_CANTO2_12SEP2017",REFDataPath , fixed=TRUE)

test_parameters2<-grepl("DRFZ_CANTO2_21OCT2015",REFDataPath , fixed=TRUE)

if (test_nb_colname & length(colnames(PB_REF@exprs))<12){
        colnames(PB_REF@exprs)[3:10]<- fluo.channels
        colnames(PB_REF)[3:10]<- fluo.channels
} else if ( test_parameters1 | test_parameters2 ){
        colnames(PB_REF@exprs)[8:15]<- fluo.channels
        colnames(PB_REF)[8:15]<- fluo.channels
} else{
        colnames(PB_REF@exprs)[7:14]<- fluo.channels
        colnames(PB_REF)[7:14]<- fluo.channels
}
```

```
PB_center <- flowCore::read.FCS(file.path(PEAKSDataPath),dataset=2)

colnames(PB_center@exprs)[7:14]<- fluo.channels

colnames(PB_center)[7:14]<- fluo.channels
```

#Extract the MFI for the 8 peak beads and the reference as 2 matrices.

```
border_lim_REF<-border(PB_REF,REFDataPath)

border_lim_center<-border(PB_center,PEAKSDataPath)
```

#Test if the file is from Beckman Coulter or from Beckton Dickinson.

```
test_lmd <- grepl(".LMD",PEAKSDataPath , fixed=TRUE)

if (test_lmd){

        ext=".LMD"

        mult=1024

} else{

        ext=".fcs"

        mult=256

}
```

#Put data in same scale as kaluza (facilitate manual comparisons)

```
y<-do.call(cbind,border_lim_REF)/mult

x<-do.call(cbind,border_lim_center)/mult
```

#Save the matrices

```
refnfile=basename(REFDataPath)
```

refCSVPath = paste("C:/Users/Beckman-Coulter/Documents/PRECISESADS_DATA/REPORTS/8PEAKBEADS/MATRICES/",refnfile,"_REF.csv",sep="")

write.csv(y,refCSVPath)

nfile=basename(PEAKSDataPath)

CSVPath = paste("C:/Users/Beckman-Coulter/Documents/PRECISESADS_DATA/REPORTS/8PEAKBEADS/MATRICES/",nfile,".csv",sep="")

write.csv(x,CSVPath)

#Define the transformation parameters alpha and beta that will normalise the fcs files

trans <-do.call('rbind', lapply(fluo.channels, function(chan) coefficients(lm(y[,chan] ~ x[,chan]))))

rownames(trans) <- fluo.channels

colnames(trans) <- c('alpha','beta')

#Save the normalisation parameters

nfile=basename(PEAKSDataPath)

transCSVPath = paste("C:/Users/Beckman-Coulter/Documents/PRECISESADS_DATA/REPORTS/NORMALISATION/trans_",nfile,".csv",sep="")

write.csv(trans,transCSVPath)

#Check if the alpha parameter is greater than 1

#Because it can move the low intensities to another decade

#Choice to only use the beta value(still reduce variation for the MFI)

#if yes save the information in a file and change the value to 0.

```
ErrorTransPath<-"C:/Users/Beckman-
Coulter/Documents/PRECISESADS_DATA/REPORTS/NORMALISATION/high_alpha_norm.txt"
for (chan_t in fluo.channels){
	intra_alpha <-trans[chan_t,'alpha']
	if (abs(intra_alpha) > 1){
		write(paste(nfile,chan_t,intra_alpha,sep=";"),ErrorTransPath,append = TRUE)
	}
	trans[chan_t,'alpha']<-0
}
```

#Define the normalisation function.

```
normalisation <- lapply(fluo.channels , function(n) return(function(x)  trans[n,'alpha'] +
x*trans[n,'beta']) )
names(normalisation) <- fluo.channels
```

#Liste the panels that will be normalised.

```
Panels_fcs <- list.files(pattern = ext, flowDataPath,recursive=F, full = TRUE)
for (fcs_file in Panels_fcs){
	fcs <- flowCore::read.FCS(file.path(fcs_file),dataset=2)
	colnames(fcs)[7:14]<- fluo.channels
	if (is.null(fcs@description$SPILL)){
		colnames(fcs@description$'$SPILLOVER')<- fluo.channels
	}	else{
		colnames(fcs@description$SPILL)[1:8]<- fluo.channels
	}
```

```
X <- fcs@exprs/mult
for (chan in fluo.channels){
    x.norm <- normalisation[[chan]](X[,chan])
    fcs@exprs[,chan] <- x.norm*mult
}

filename = paste(fcs_file,"_intra.fcs",sep="")
write.FCS(fcs,filename , delimiter = "\\")
```

# Test if the application of the normalisation reduce the variation to acceptable level in all the channel

```
test_QC <- grepl("QC8",fcs_file , fixed=TRUE)
if( test_QC){
    ErrorPath<-"C:/Users/Beckman-Coulter/Documents/PRECISESADS_DATA/REPORTS/8PEAKBEADS/CHECK_NORM/8peakbeads_norm_mistake.txt"
    border_norm_center<-border(fcs,filename)
    xnorm<-do.call(cbind,border_norm_center)/mult

    dif_norm <- ((y-xnorm)/y)*100
    for (chan in fluo.channels){
        nb_peak <- function(type) {
        switch(type,"FITC.A"=8,
                    "PE.A"=8,
                    "PC5.5.A"=6,
```

```
"PC7.A"=6,
"APC.A"=8,
"APC.AF750.A"=8,
"PB.A"=8,
"KO.A"=8)
}
starting_peak <- function(type) {
switch(type,"FITC.A"=2,
"PE.A"=2,
"PC5.5.A"=2,
"PC7.A"=3,
"APC.A"=2,
"APC.AF750.A"=4,
"PB.A"=2,
"KO.A"=2)
}
peak_nb<-nb_peak(chan)
peak<- starting_peak(chan)
while(peak <= peak_nb){
peak_value_dif <- dif_norm[peak,chan]
if (peak_value_dif > 5){

write(paste(basename(fcs_file),chan,peak,peak_value_dif,sep=";"),ErrorPath,append =
TRUE)
}
```

```
                peak<-peak+1
            }
        }
    }
}

print("intra normalisation is over for this patient")
```

Supplementary Document 2. Python script for the data correction of a single flow cytometer.

```
#!/usr/bin/python
# Lucas Le Lann
#12/10/2018

### Importation of usefull modules
from collections import defaultdict
from function_cytometry_results import *
from numpy import median
from datetime import datetime
import os.path
import glob, os
import matplotlib.pyplot as plt
import matplotlib.cbook as cbook
import numpy as np
import pandas as pd
import csv
import re

### FUNCTION

# Extract the Date from the path to the FCS files for the OMIC ID
searched
# If missing return 0
def get_DATE(OMICID):
    path="E:/PRECISESADS_DATA/PATIENTS/"
    for PHASE in glob.glob(path+"*"):
        for center in glob.glob(PHASE+"/*"):
            for patient in glob.glob(center+"/*"):
                OMIC=patient.split("\\")[-1]
                if OMIC==OMICID:
                    list_files=glob.glob(patient+"/*")
                    DATE=list_files[0].split("_")[-1].split(".")[0]
                    return DATE
        return 0

# Reformate the date ( change to a numeral definition for the month)
# Taking into acount some of the mistake for the writing of the
month.
# (like using German or spanish abreviation for the month)
def formating_date(date):
    if date == "DATE" or "-" in date:
        return date
    elif date == "":
        date="DATE"
    else:
        print date

    date=date.replace(".fcs","")
    date=date.replace(".LMD","")
    date=date.replace(".csv","")
    date=date.replace("JAN","/01/")
    date=date.replace("FEB","/02/")
    date=date.replace("FEV","/02/")
    date=date.replace("MAR","/03/")
    date=date.replace("APR","/04/")
```

```
    date=date.replace("AVR","/04/")
    date=date.replace("MAY","/05/")
    date=date.replace("MAI","/05/")
    date=date.replace("JUN","/06/")
    date=date.replace("Jun","/06/")
    date=date.replace("JUL","/07/")
    date=date.replace("AUG","/08/")
    date=date.replace("AGU","/08/")
    date=date.replace("SEP","/09/")
    date=date.replace("OCT","/10/")
    date=date.replace("Oct","/10/")
    date=date.replace("OKT","/10/")
    date=date.replace("NOV","/11/")
    date=date.replace("DEC","/12/")
    date=date.replace("DIC","/12/")
    date=date.replace("T","")
    date=date.replace("IL","")
    date=date.replace(" ","")
    date=date.replace(".","")
    tmp_date=date.split("/")
    print tmp_date
    if len(list(tmp_date[2]))== 2:
        tmp_date[2]="20"+tmp_date[2]
    newdate=tmp_date[2]+"-"+tmp_date[1]+"-"+tmp_date[0]
    return newdate

# Search inside the transmart file to get the date of acquisition
for the ID of the patient
def search_date(OMICID):
    PHASEI={}
    PHASEII={}
    INCEPTION={}
    with open("C:/Users/Beckman-
Coulter/Documents/PRECISESADS_DATA/TRANSMART/transmart_15_06_2018_PH
ASE_I_II.tsv","r") as transmart_phase:
        header=0
        transmart_phase.seek(0)
        for line in transmart_phase:
            if header==0:
                header=1
            line=line.split("\t")
            col_nb=0
            for head in line:
                if "number" in head:
                    OMICID_col=col_nb
                elif "Sampling\Date Of Sampling" in head:
                    DATE_col=col_nb
                elif "CS Phase" in head:
                    PHASE_col=col_nb
                    col_nb+=1
                else:
                    line=line.split("\t")
                    OMICID_tm=line[OMICID_col]

                DATE_tm=line[DATE_col]
                if "II" in line[PHASE_col]:
                    PHASEII[OMICID_tm]=DATE_tm
```

```
                elif "I" in line[PHASE_col]:
                    PHASEI[OMICID_tm]=DATE_tm

    with open("C:/Users/Beckman-
Coulter/Documents/PRECISESADS_DATA/TRANSMART/transmart_15_06_2018_in
ception.tsv","r") as transmart_phase:
        header=0
        for line in transmart_phase:
            if header==0:
                header=1
                line=line.split("\t")
                col_nb=0
                for head in line:
                    if "Sampling\OMIC number\M000" in head:
                        OMICID_M00_col=col_nb
                    elif "Sampling\OMIC number\M006" in head:
                        OMICID_M06_col=col_nb
                    elif "Sampling\OMIC number\M014" in head:
                        OMICID_M14_col=col_nb
                    elif "Date Of Sampling\M000" in head:
                        DATE_M00_col=col_nb
                    elif "Date Of Sampling\M006" in head:
                        DATE_M06_col=col_nb
                    elif "Date Of Sampling\M014" in head:
                        DATE_M14_col=col_nb
                    col_nb+=1
            else:
                line=line.split("\t")
                OMICID_M00_tm=line[OMICID_M00_col]
                DATE_M00_tm=line[DATE_M00_col]
                INCEPTION[OMICID_M00_tm]=DATE_M00_tm+"_M0"
                OMICID_M06_tm=line[OMICID_M06_col]
                DATE_M06_tm=line[DATE_M06_col]
                INCEPTION[OMICID_M06_tm]=DATE_M06_tm+"_M6"
                OMICID_M14_tm=line[OMICID_M14_col]
                DATE_M14_tm=line[DATE_M14_col]
                INCEPTION[OMICID_M14_tm]=DATE_M14_tm+"_M14"
                OMICID="\"N"+OMICID+"\""
                if OMICID in PHASEII.keys():
                    if get_DATE(OMICID) == 0:
                        DATE=PHASEII[OMICID]
                    else:
                        DATE=get_DATE(OMICID)
                elif OMICID in PHASEI.keys():
                    if get_DATE(OMICID) == 0:
                        DATE=PHASEI[OMICID]
                    else:
                        DATE=get_DATE(OMICID)
                elif OMICID in INCEPTION.keys():
                    if get_DATE(OMICID) == 0:
                        DATE=INCEPTION[OMICID].split("_")[0]
                        DATE=DATE.replace("\"","")
                    else:
                        DATE=get_DATE(OMICID)
                else:
                    DATE="DATE"
                    print "MISSING "+ OMICID
```

```
                DATE=formating_date(DATE)
        return DATE
```

# Get the coefficient for the correction of the Batch effect.
# Based on the date of the acquisition of the file

```
def get_coef(PANEL,CENTER,CHANNEL,DATE):
    coefFile =
open("./MFI/MATRICES/COEF_BATCH/matrice_panel_"+PANEL+"_coef_batch.c
sv", 'r')

    if DATE == "2014-12-31":
        col=2
    elif DATE == "2015-06-22":
        col=3
    elif DATE == "2015-10-19":
        col=4
    elif DATE == "2016-01-14":
        col=5
    elif DATE == "2016-04-14":
        col=6
    elif DATE == "2016-09-06":
        col=7
    elif DATE == "2017-01-09":
        col=8
    elif DATE == "2017-04-14":
        col=9
    elif DATE == "2017-09-11":
        col=10
    elif DATE == "2017-12-11":
        col=11
    else:
        print "Date did not match"


    for line in coefFile:
        line=line.split(";")
        if line[0] == CENTER and line[1]==CHANNEL:
            return line[col]
```

# Formating the month for easier use later as a numerical value

```
def formating_month(month):
    month=month.replace("JAN","01")
    month=month.replace("FEB","02")
    month=month.replace("FEV","02")
    month=month.replace("MAR","03")
    month=month.replace("APR","04")
    month=month.replace("AVR","04")
    month=month.replace("MAY","05")
    month=month.replace("MAI","05")
    month=month.replace("JUN","06")
    month=month.replace("Jun","06")
    month=month.replace("JUL","07")
    month=month.replace("AUG","08")
    month=month.replace("AGU","08")
    month=month.replace("SEP","09")
    month=month.replace("OCT","10")
```

```
    month=month.replace("Oct","10")
    month=month.replace("OKT","10")
    month=month.replace("NOV","11")
    month=month.replace("DEC","12")
    month=month.replace("DIC","12")
    month=month.replace("T","")
    month=month.replace("IL","")
    month=month.replace(" ","")
    month=month.replace(".","")
    return month

# Test if the date fit the required format
# And changed it if not
def testdate(date):
    match=re.search("^\d{2}\D{3}\d{2}",date)
    if match:
        sdate=list(date)
        month=sdate[2]+sdate[3]+sdate[4]
        newdate="20"+sdate[5]+sdate[6]+"-"+formating_month(month)+"-
"+sdate[0]+sdate[1]
        return newdate
    else:
        return date

#### END THE DEFINITION OF FUNCTIONS

print "start"
print " clear folders"
clear_panel=True

# Remove the files of previous iteration of the script
if clear_panel:
    for fichiers in glob.glob("./MFI/CORRECTED_MFI/*.csv"):
    os.remove(fichiers)
    for fichiers in glob.glob("./MFI/BATCH_MFI/*.csv"):
    os.remove(fichiers)
    for fichiers in glob.glob("./MFI/CENTER_MFI/*.csv"):
    os.remove(fichiers)

print "MFI folder clear"
print "define list of blacklisted OMICs"
### A list of files have been excludes due to external reasons like
decoupling or defined as outliers
### After verification some of those outliers have been validated as
biological outlier

# Setting the dictionary
list_panel=["P1","P2"]
BL={}
WL={}
for element in list_panel:
    BL[element]=[]
    WL[element]=[]
blacklist=open("detailedblacklist.txt","r")
whitelist=open("detailedwhitelist.txt","r")

##create list of whitelisted OMIC ID for each PANEL
```

```
for line_w in whitelist:
    OMICID_w=line_w.split(";")[0]
    PANELID_w=line_w.split(";")[2].replace("\n","")
    if PANELID_w == "ALL":
        for ID_w in WL.keys():
            WL[ID_w].append(OMICID_w)
    else:
        WL[PANELID_w].append(OMICID_w)

##create list of Blacklisted OMIC ID for each PANEL that are not in
the whitelisted list
for line in blacklist:
    OMICID=line.split(";")[0]
    PANELID=line.split(";")[2].replace("\n","")
    if PANELID == "ALL":
        for ID in BL.keys():
            BL[ID].append(OMICID)
    else:
        if OMICID not in WL[PANELID]:
            BL[PANELID].append(OMICID)

print "OMICs blacklist done"
print "### standardized channel names and add DATE ###"
count_panel=1
panel_nb_wanted=2
if True:
    while count_panel <= panel_nb_wanted:
        list_REF=[]
        for file in
glob.glob("./MFI/RAW_MFI/*"+str(count_panel)+".csv"):
            with open(file,"r") as MFI_file:
                IC=file.split("\\")[1]
                OMIC=IC.split("_")[0]
                date=search_date(OMIC)
                newMFI_file=file.replace("RAW_MFI","CORRECTED_MFI")

newMFI_file=newMFI_file.replace("DATE",date.replace("\"",""))
            if not os.path.isfile(newMFI_file) and OMIC not in
BL["P"+str(count_panel)]:
                with open(newMFI_file,"a") as newMFI_file:
                    header=0
                    for line in MFI_file:
                        sline=line.split("\t")
                        REF= sline[3]
                        REF=REF.replace("-",".")
                        list_REF.append(REF)
                        if count_panel == 1:
                            if "CD3" in REF or "APC.AF750" in REF :
                                REF= "CD3 APC.AF750"
                            elif "CD19" in REF or ("APC" in REF and
not "APC.AF750" in REF):
                                REF= "CD19 APC"
                            elif "CD16" in REF or "FITC" in REF:
                                REF= "CD16 FITC"
                            elif "CD15" in REF or "PE" in REF:
                                REF= "CD15 PE"
                            elif "CD56" in REF or "PC5.5" in REF:
```

```
                            REF= "CD56 PC5.5"
                        elif "CD14" in REF or "PC7" in REF:
                            REF= "CD14 PC7"
                        elif "CD4" in REF or "PB" in REF:
                            REF= "CD4 PB"
                        elif "CD8" in REF or "KO" in REF:
                            REF= "CD8 KO"
                        else:
                            print REF
                    elif count_panel == 2:
                        if "CD123" in REF or ("APC" in REF and
not "APC.AF750" in REF):
                            REF= "CD123 APC"
                        elif "CD1c" in REF or "FITC" in REF:
                            REF= "CD1c FITC"
                        elif "Lin" in REF or "PE" in REF:
                            REF= "Lin PE"
                        elif "CD141" in REF or "PC5" in REF:
                            REF= "CD56 PC5.5"
                        elif "CD11c" in REF or "PC7" in REF:
                            REF= "CD11c PC7"
                        elif "HLA" in REF or "PB" in REF:
                            REF= "HLADR PB"
                        else:
                            print REF
                    else:
                        print count_panel

                    if header==0:
                        header=1

newline=sline[0]+"\t"+sline[1]+"\t"+sline[2]+"\t"+REF+"\t"+sline[4].
replace("\n","")+"\tDATE\n"
                    else:

newline=sline[0]+"\t"+sline[1]+"\t"+sline[2]+"\t"+REF+"\t"+sline[4].
replace("\n","")+"\t"+date+"\n"
                        newMFI_file.write(newline)
        count_panel+=1

print "### CORRECTED DATE done ###"
list_chan=('channel','FITC','PE','PC5.5','PC7','APC','AF750','PB','K
O')
list_centre=("CHP","DRFZ","FPS","IRCCS","IDIBELL","MHH","KUL","SAS",
"UBO","UCL","UNIGE")
```

### COMPUTE the coefficient for each of the batches
### Then applied each coefficient to the MFI and saved in a new csv file

```
print "### BEGINING BATCH COEF ###"
if True:
    panel=1
    while panel<=panel_nb_wanted:
        value_dict={}
        if panel == 1:
```

```
            coef_file=
"./MFI/MATRICES/COEF_BATCH/matrice_panel_1_coef_batch.csv"
            med_file=
"./MFI/MATRICES/COEF_BATCH/matrice_panel_1_med_batch.csv"
            list_pop_selected=("CD15lowCD16high_Neutrophils_CD16
FITC","PMN_CD15 PE","CD3negCD56pos_NKcells_CD56
PC5.5","CD14highCD16neg_classicalMonocytes_CD14
PC7","CD19pos_Bcells_CD19 APC","CD3pos_Tcells_CD3
APC.AF750","CD4pos_Tcells_CD4 PB","CD8pos_Tcells_CD8 KO")
        elif panel == 2:
            coef_file=
"./MFI/MATRICES/COEF_BATCH/matrice_panel_2_coef_batch.csv"
            med_file=
"./MFI/MATRICES/COEF_BATCH/matrice_panel_2_med_batch.csv"
            list_pop_selected=("mDC1_CD1c FITC","mDC2_CD56
PC5.5","mDC_CD11c PC7","BASO_CD123 APC","DRpos_linneg_HLADR PB")
        else:
            print "Panel number not implemented"

        os.remove(coef_file)
        f=open(coef_file,"a")
        f.write("CENTER;CHANNEL;X2015.10.19;X2017.04.14\n")
        f.close()

        os.remove(med_file)
        f=open(med_file,"a")
        f.close()

        for panel_csv in
glob.glob("./MFI/CORRECTED_MFI/*PANEL_"+str(panel)+".csv"):
                 first_line=True
            site=panel_csv.split("_")[2]
            if "CHAR" in site:
                site="DRFZ"
            if site == "IDIBEL":
                site="IDIBELL"
            for line in open(panel_csv ,"r"):
                if first_line:
                    OMIC=line.split("\t")[0]
                    first_line=False
                else:
                    line=line.split("\t")
                    pop=line[1]
                    ref=line[3]
                    pop_ref=pop+"_"+ref
                    value = line[4]
                    date=line[5].replace("\n","")
                    date=testdate(date)
                    # determination whitch "batch" the patient is in
                    if "DATE" not in date and date != '':
                        date=date.replace("\"","")
                        date=datetime.strptime(date, '%Y-%m-%d')
                        ref_batch=datetime.strptime("2015-06-22",
'%Y-%m-%d')
                        first_batch=datetime.strptime("2015-10-19",
'%Y-%m-%d')
```

```
                        calibration_01=datetime.strptime("2016-01-
14", '%Y-%m-%d')
                        calibration_02=datetime.strptime("2016-04-
14", '%Y-%m-%d')

                        second_batch=datetime.strptime("2016-09-06",
'%Y-%m-%d')
                        third_batch=datetime.strptime("2017-01-09",
'%Y-%m-%d')

                        calibration_03=datetime.strptime("2017-04-
14", '%Y-%m-%d')
                        calibration_04=datetime.strptime("2017-09-
11", '%Y-%m-%d')

                        forth_batch=datetime.strptime("2017-12-11",
'%Y-%m-%d')
                        #print line[3]
                        channel=line[3].split(" ")[1]
                        if date < ref_batch:
                            batch="liquid"
                        elif ref_batch<= date < first_batch:
                            batch= "ref"
                        elif first_batch<= date < calibration_01:
                            batch= "1"
                        elif calibration_01<= date < calibration_02:
                            batch= "cal_1"
                        elif calibration_02<= date < second_batch:
                            batch= "cal_2"
                        elif second_batch<= date < third_batch:
                            batch= "2"
                        elif third_batch<= date < calibration_03:
                            batch= "3"
                        elif calibration_03<= date < calibration_04:
                            batch= "cal_3"
                        elif calibration_04<= date < forth_batch:
                            batch= "cal_4"
                        elif forth_batch<= date :
                            batch= "4"


                    if pop_ref in list_pop_selected:
                        batch = str(batch)
                        if batch not in value_dict.keys():
                            value_dict[batch]={}
                            for centre in list_centre:
                                value_dict[batch][centre]={}
                                for refpop in list_pop_selected:

value_dict[batch][centre][refpop]=[]
                        if value != "NA" and value != "" and value
!= "N/A":
                            print "############"
                            print OMIC
                            print value

value_dict[batch][site][pop_ref].append(float(value))
```

```
        batch_med=value_dict
        for lot in value_dict.keys():
            for center in value_dict[lot].keys():
                print center
                for pop in value_dict[lot][center].keys():
                    if value_dict[lot][center][pop] != []:


number_used_for_med=len(value_dict[lot][center][pop])
                        #if center=="UBO" and "CD19" in pop:
                            #print sum(value_dict[lot][center][pop])
                            #print len(value_dict[lot][center][pop])
                        med=median(value_dict[lot][center][pop])
                        batch_med[lot][center][pop]=med

                        mf=open(med_file,"a")
                        mf.write(lot +";"+center + ";"+
pop+";"+str(med)+";"+str(number_used_for_med)+"\n")
                        mf.close()
        print "###################"
        print panel
        print value_dict.keys()

        for center in value_dict[batch_ref].keys():
            for pop in value_dict[batch_ref][center].keys():

                med_ref=value_dict[batch_ref][center][pop]
                med_center_liq=value_dict["liquid"][center][pop]
                med_center_1=value_dict["1"][center][pop]
                med_center_2=value_dict["2"][center][pop]
                med_center_3=value_dict["3"][center][pop]
                med_center_4=value_dict["4"][center][pop]

                med_center_cal_1=value_dict["cal_1"][center][pop]
                med_center_cal_2=value_dict["cal_2"][center][pop]
                med_center_cal_3=value_dict["cal_3"][center][pop]
                med_center_cal_4=value_dict["cal_4"][center][pop]

                if isinstance(med_ref, float) :
                    if isinstance(med_center_liq, float):

coef_batch_liq=float(med_ref)/float(med_center_liq)
                    else:
                        coef_batch_liq= 1

                    if isinstance(med_center_1, float):

coef_batch_1=float(med_ref)/float(med_center_1)
                    else:
                        coef_batch_1=1

                    if isinstance(med_center_2, float):

coef_batch_2=float(med_ref)/float(med_center_2)
                    else:
                        coef_batch_2=1
```

```
                    if isinstance(med_center_3, float):

coef_batch_3=float(med_ref)/float(med_center_3)
                    else:
                        coef_batch_3=1

                    if isinstance(med_center_4, float):

coef_batch_4=float(med_ref)/float(med_center_4)
                    else:
                        coef_batch_4=1

                    if isinstance(med_center_cal_1, float):

coef_batch_cal_1=float(med_ref)/float(med_center_cal_1)
                    else:
                        coef_batch_cal_1=1

                    if isinstance(med_center_cal_2, float):

coef_batch_cal_2=float(med_ref)/float(med_center_cal_2)
                    else:
                        coef_batch_cal_2=1

                    if isinstance(med_center_cal_3, float):

coef_batch_cal_3=float(med_ref)/float(med_center_cal_3)
                    else:
                        coef_batch_cal_3=1

                    if isinstance(med_center_cal_4, float):

coef_batch_cal_4=float(med_ref)/float(med_center_cal_4)
                    else:
                        coef_batch_4=1

                cf=open(coef_file,"a")
                chan=pop.split(" ")[1]

cf.write(center+";"+chan+";"+str(coef_batch_liq)+";1;"+str(coef_batc
h_1)+";"+str(coef_batch_cal_1)+";"+str(coef_batch_cal_2)+

";"+str(coef_batch_2)+";"+str(coef_batch_3)+";"+str(coef_batch_cal_3
)+";"+str(coef_batch_cal_4)+";"
                +str(coef_batch_4)+"\n")
                cf.close()

        panel+=1
    print "END COMPUTING COEF BATCH"

    ### APPLICATION OF THE COEF BATCH

if True:
    print "###   BEGINING APPLY BATCH COEF   ###"
    panel=1
    while panel<=panel_nb_wanted:
```

```
        for MFI_files in
glob.glob("./MFI/CORRECTED_MFI/*"+str(panel)+".csv"):
            print MFI_files

new_MFI_files=MFI_files.replace("CORRECTED_MFI","BATCH_MFI")
            #print MFI_files.split("_")
            CENTER=MFI_files.split("_")[2]
            if "CHAR" in CENTER :
                CENTER="DRFZ"
            elif CENTER == "IDIBEL":
                CENTER="IDIBELL"
            panel_s=MFI_files.split("_")[-1].replace(".csv","")
            MFI_file=open(MFI_files,"r")
            new_MFI_file=open(new_MFI_files,"w")

            for ligne in MFI_file:
                ligne=ligne.split("\t")
                date=ligne[5].replace("\n","")
                date=testdate(date)
                if "DATE" not in date and date != '':
                    date=date.replace("\"","")
                    date=datetime.strptime(date, '%Y-%m-%d')
                    ref_batch=datetime.strptime("2015-06-22", '%Y-
%m-%d')
                    first_batch=datetime.strptime("2015-10-19", '%Y-
%m-%d')

                    calibration_01=datetime.strptime("2016-01-14",
'%Y-%m-%d')
                    calibration_02=datetime.strptime("2016-04-14",
'%Y-%m-%d')

                    second_batch=datetime.strptime("2016-09-06",
'%Y-%m-%d')
                    third_batch=datetime.strptime("2017-01-09", '%Y-
%m-%d')

                    calibration_03=datetime.strptime("2017-04-14",
'%Y-%m-%d')
                    calibration_04=datetime.strptime("2017-09-11",
'%Y-%m-%d')

                    forth_batch=datetime.strptime("2017-12-11", '%Y-
%m-%d')
                    channel=ligne[3].split(" ")[1]

                    if "HLA" in ligne[3]:
                        channel="linneg_HLADR"

                    if date < ref_batch:
                        coef=get_coef(panel_s,CENTER,channel,"2014-
12-31")
                    elif ref_batch<= date < first_batch:
                        coef=get_coef(panel_s,CENTER,channel,"2015-
06-22")
                    elif first_batch<= date < calibration_01:
```

```
                        coef=get_coef(panel_s,CENTER,channel,"2015-
10-19")
                    elif calibration_01<= date < calibration_02:
                        coef=get_coef(panel_s,CENTER,channel,"2016-
01-14")
                    elif calibration_02<= date < second_batch:
                        coef=get_coef(panel_s,CENTER,channel,"2016-
04-14")
                    elif second_batch<= date < third_batch:
                        coef=get_coef(panel_s,CENTER,channel,"2016-
09-06")
                    elif third_batch<= date < calibration_03:
                        coef=get_coef(panel_s,CENTER,channel,"2017-
01-09")
                    elif calibration_03<= date < calibration_04:
                        coef=get_coef(panel_s,CENTER,channel,"2017-
04-14")
                    elif calibration_04<= date < forth_batch:
                        coef=get_coef(panel_s,CENTER,channel,"2017-
09-11")
                    elif forth_batch<= date :
                        coef=get_coef(panel_s,CENTER,channel,"2017-
12-11")

                    else:
                        print MFI_file
                        pause()
                    if "Lin" in ligne[3] or "lin" in channel:
                        coef=1
                    if ligne[4] == "NA":
                        newMFI="NA"
                    else:
                        if coef == None:
                            print panel_s
                            print channel
                            print MFI_files
                            print date
                        try:
                            newMFI=float(ligne[4])*float(coef)
                        except:
                            newMFI=ligne[4]
                else:
                    newMFI=ligne[4]

text=ligne[0]+"\t"+ligne[1]+"\t"+ligne[2]+"\t"+ligne[3]+"\t"+str(new
MFI)+"\t"+str(date)+"\n"
                new_MFI_file.write(text)
            MFI_file.close()
            new_MFI_file.close()
        panel+=1

    print " END APPLYING COEF BATCH"
```

Supplementary Document 3. Python script for the data correction between all flow cytometers.

```
#!/usr/bin/python
# Lucas Le Lann
#12/10/2018

### Importation of usefull packages
from collections import defaultdict
from numpy import median
from datetime import datetime
import os.path
import glob, os
import numpy as np
import re
import subprocess

### FUNCTION

# Extract the coefficient for the different cytometers used for each
center
def get_coef_center(PANEL,CENTER,CHANNEL):
    coefFile =
open("./MATRICES/COEF/matrice_panel_"+str(PANEL)+"_coef_"+CENTER+".c
sv", 'r')
    for line in coefFile.readlines():
        line=line.split(";")
        if CHANNEL == line[0].replace("\"",""):
            coef_cen=line[1].replace("\n","")
            return coef_cen

### END FUNCTION

### COMPUTE the coefficient for each of the machine/center
### Then applied each coefficient to the MFI saved in a new csv file

print "### BEGINING COMPUTING CENTER COEF ###"
### COMPUTE COEFFICIENT CENTER
panel=1
panel_nb_wanted=2
while panel<=panel_nb_wanted:
    value_dict={}
    if panel == 1:
        coef_file= "./MATRICES/MEDIANS/matrice_panel_1_med_"

list_cen=("CHP","DRFZ","FPS","IDIBELL","IRCCS","KUL","MHH","SAS","UB
O","UCL","UNIGE")
    elif panel == 2:
        coef_file= "./MATRICES/MEDIANS/matrice_panel_2_med_"

list_cen=("CHP","DRFZ","FPS","IDIBELL","IRCCS","KUL","MHH","SAS","UB
O","UCL","UNIGE")
    else:
        print "Panel number not implemented"

    for cen in list_cen:
        f=open(coef_file+cen+".csv","w")
        f.write("CENTER;CHANNEL;POPULATION;MEDIANS;NB_USED\n")
```

```
        f.close()

    for panel_csv in glob.glob("./BATCH_MFI/*"+str(panel)+".csv"):
        first_line=True
        site=panel_csv.split("_")[2]
        if "CHAR" in site:
            site="DRFZ"
        if site == "IDIBEL":
            site="IDIBELL"

        for line in open(panel_csv ,"r"):
            if first_line:
                OMIC=line.split("\t")[0]
                first_line=False
            else:
                line=line.split("\t")
            pop=line[1]
            ref=line[3]
            value = line[4]
            chan=ref.split(" ")[0]
            if site not in value_dict.keys():
                value_dict[site]={}
                if ref not in value_dict[site].keys():
                    value_dict[site][ref]={}
                    if pop not in value_dict[site][ref].keys():
                        value_dict[site][ref][pop]=[]
                        if value != "NA" or value != "":
                        try:
                            if float(value) > 1000.0:
                                print "extract values greater than
1000"

log_save_file=open("./MATRICES/VALUE_USED/log.csv","a")

log_save_file.write(str(panel)+";"+site +
";"+OMIC+";"+ref+";"+pop+";"+str(value)+"\n")
                            else:

value_dict[site][ref][pop].append(float(value))
                        except:
                            print str(OMIC)+" error in " +
str(value)+ "pop "+ pop


    center_med=value_dict
    for center in value_dict.keys():
        for reference in value_dict[center].keys():
            for population in value_dict[center][reference].keys():

med=median(value_dict[center][reference][population])
                if "nan" in str(med) or
len(value_dict[center][reference][population]) <= 5:
                    print "missing median"
                else:

med_save_file=open("./MATRICES/MEDIANS/matrice_panel_"+str(panel)+"_
med_"+center+".csv","a")
```

```
                med_save_file.write(center +
";"+reference+";"+population+";"+str(med)+";"+str(len(value_dict[cen
ter][reference][population]))+"\n")
                med_save_file.close()


val_save_file=open("./MATRICES/VALUE_USED/matrice_panel_"+str(panel)
+"_values_"+center+".csv","a")
                val_save_file.write(center +
";"+reference+";"+population+";"+str(med))
                for val in
value_dict[center][reference][population]:
                   val_save_file.write(";"+str(val))
                val_save_file.write("\n")
                val_save_file.close()
    panel+=1
    coef_center()
print " END COMPUTING COEF CENTER"
```

### APPLICATION OF THE COEF CENTER

```
print " APPLY COEF CENTER "
panel=1
while panel<=panel_nb_wanted:
    for MFI_files in glob.glob("./BATCH_MFI/*"+str(panel)+".csv"):
        new_MFI_files=MFI_files.replace("BATCH_MFI","CENTER_MFI")
        CENTER=MFI_files.split("_")[2]
        if "CHAR" in CENTER:
            CENTER="DRFZ"
        if CENTER == "IDIBEL":
            CENTER="IDIBELL"

        omic_test=MFI_files.split("_")[1]
        omic_test=omic_test.replace("MFI\\","")
        MFI_file=open(MFI_files,"r")
        new_MFI_file=open(new_MFI_files,"w")
        first_ligne=True
        for ligne in MFI_file:
            if first_ligne:
                text=ligne
                new_MFI_file.write(text)
                first_ligne=False
            else:
                ligne=ligne.split("\t")
                channel=ligne[3]
                print str(panel)+","+CENTER+","+channel

coef_value=get_coef_center(panel,CENTER,str(channel))
                if ligne[4] == "NA" or ligne[4] == "" or ligne[4] ==
"N/A" :
                    newMFI="NA"
                else:
                    coef_value=coef_value.replace(",",".")
                    newMFI=(float(ligne[4])*float(coef_value))
                    newMFI=round(newMFI,2)

text=ligne[0]+"\t"+ligne[1]+"\t"+ligne[2]+"\t"+ligne[3]+"\t"+str(new
MFI)+"\t"+ligne[5]
```

```
            new_MFI_file.write(text)
        MFI_file.close()
        new_MFI_file.close()
panel+=1
```
