# Supplementary material for: Standardization procedure for flow cytometry data harmonization in prospective multicenter studies
Source: Sci Rep. 2020 Jul 14;10:11567. doi: 10.1038/s41598-020-68468-3 (PMC7360585; doi:10.1038/s41598-020-68468-3)
Supplement: Supplementary file 4 — Supplementary Figure 1. [file 41598_2020_68468_MOESM4_ESM.pdf]

Standardization procedure for flow cytometry data harmonization in prospective multicenter studies

Lucas Le Lann[1], PRECISESADS Flow Cytometry Study Group[1] and PRECISESADS Clinical Consortium[1], Pierre-Emmanuel Jouve[2], Marta Alarcón-Riquelme[3], Christophe Jamin[1,4], Jacques-Olivier Pers[1]

Supplementary Figure 1

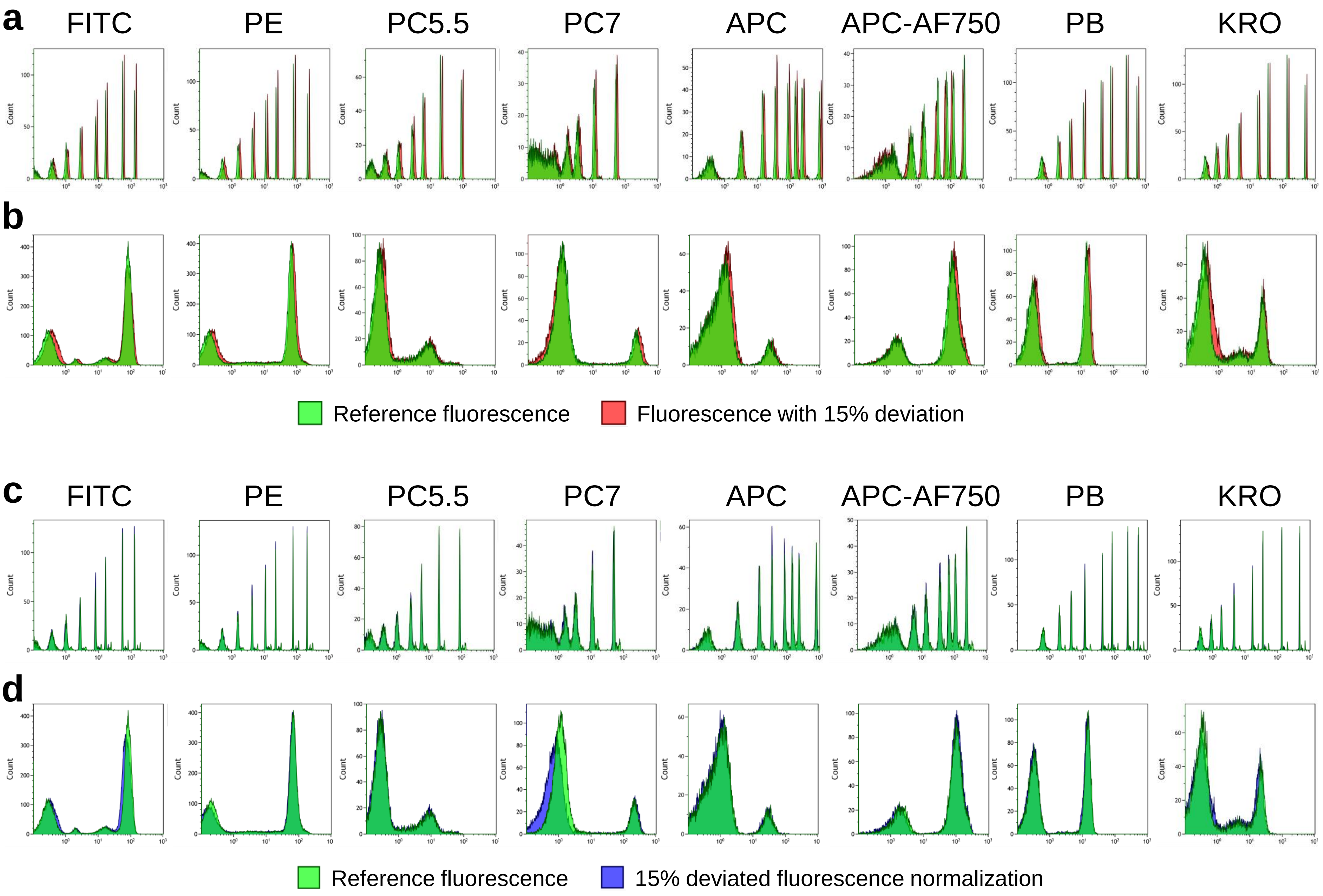
a
FITC
PE
PC5.5
PC7
APC
APC-AF750
PB
KRO
b
Reference fluorescence
Fluorescence with 15% deviation
c
FITC
PE
PC5.5
PC7
APC
APC-AF750
PB
KRO
d
Reference fluorescence
15% deviated fluorescence normalization
