# Supplementary material for: Standardization procedure for flow cytometry data harmonization in prospective multicenter studies
Source: Sci Rep. 2020 Jul 14;10:11567. doi: 10.1038/s41598-020-68468-3 (PMC7360585; doi:10.1038/s41598-020-68468-3)
Supplement: Supplementary file 5 — Supplementary Figure 2. [file 41598_2020_68468_MOESM5_ESM.pdf]

Standardization procedure for flow cytometry data harmonization in prospective multicenter studies

Lucas Le Lann[1], PRECISESADS Flow Cytometry Study Group[1] and PRECISESADS Clinical Consortium[1], Pierre-Emmanuel Jouve[2], Marta Alarcón-Riquelme[3], Christophe Jamin[1,4], Jacques-Olivier Pers[1]

Supplementary Figure 2

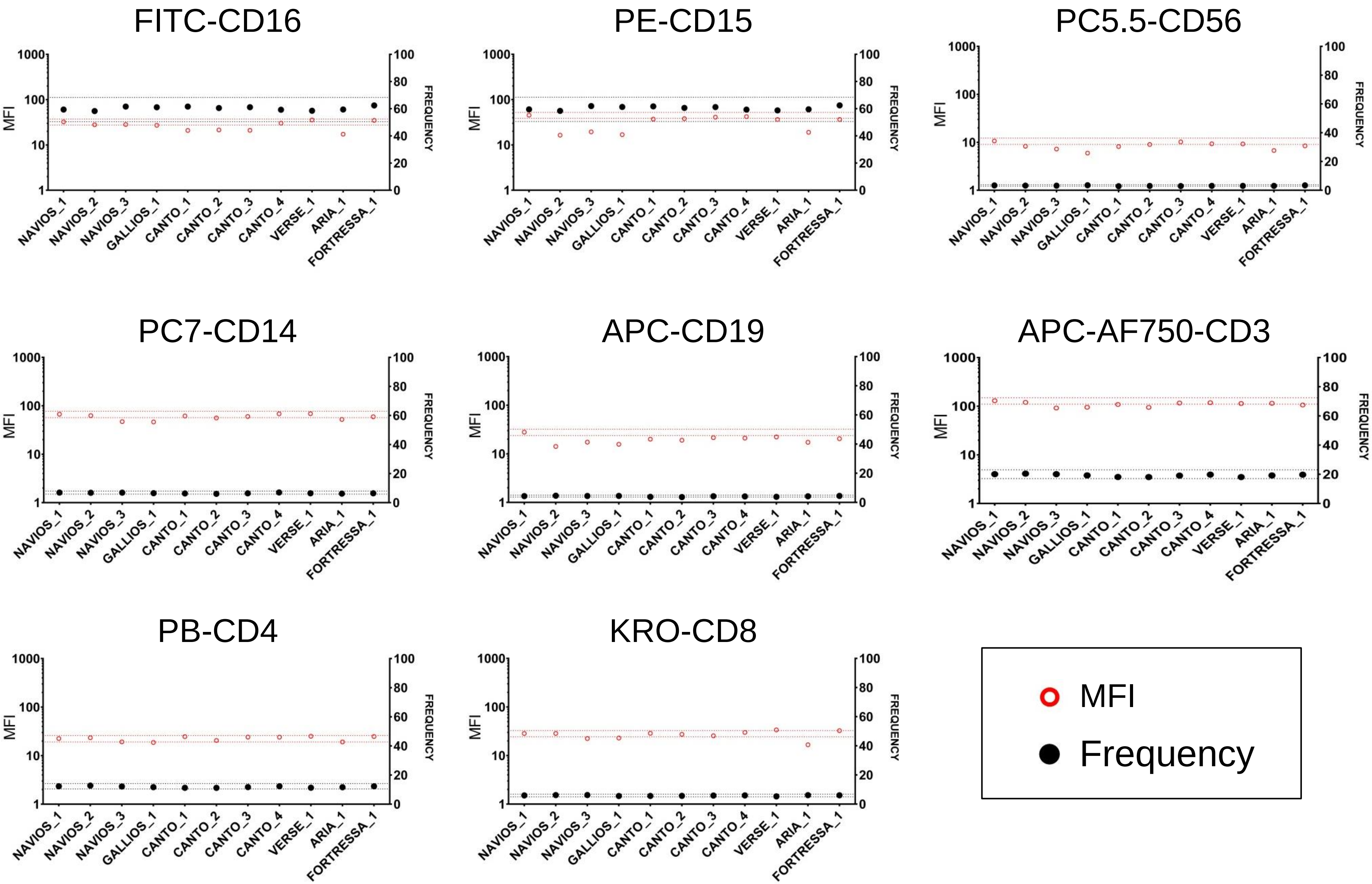
FITC-CD16
PE-CD15
PC5.5-CD56
PC7-CD14
APC-CD19
APC-AF750-CD3
PB-CD4
KRO-CD8
MFI
FREQUENCY
1000
100
10
1
100
80
60
40
20
0
NAVIOS_1
NAVIOS_2
NAVIOS_3
GALLIOS_1
CANTO_1
CANTO_2
CANTO_3
CANTO_4
VERSE_1
ARIA_1
FORTRESSA_1
MFI
Frequency
