# Supplementary material for: Standardization procedure for flow cytometry data harmonization in prospective multicenter studies
Source: Sci Rep. 2020 Jul 14;10:11567. doi: 10.1038/s41598-020-68468-3 (PMC7360585; doi:10.1038/s41598-020-68468-3)
Supplement: Supplementary file 6 — Supplementary Figure 3. [file 41598_2020_68468_MOESM6_ESM.pdf]

Standardization procedure for flow cytometry data harmonization in prospective multicenter studies

Lucas Le Lann[1], PRECISESADS Flow Cytometry Study Group[1] and PRECISESADS Clinical Consortium[1], Pierre-Emmanuel Jouve[2], Marta Alarcón-Riquelme[3], Christophe Jamin[1,4], Jacques-Olivier Pers[1]

Supplementary Figure 3

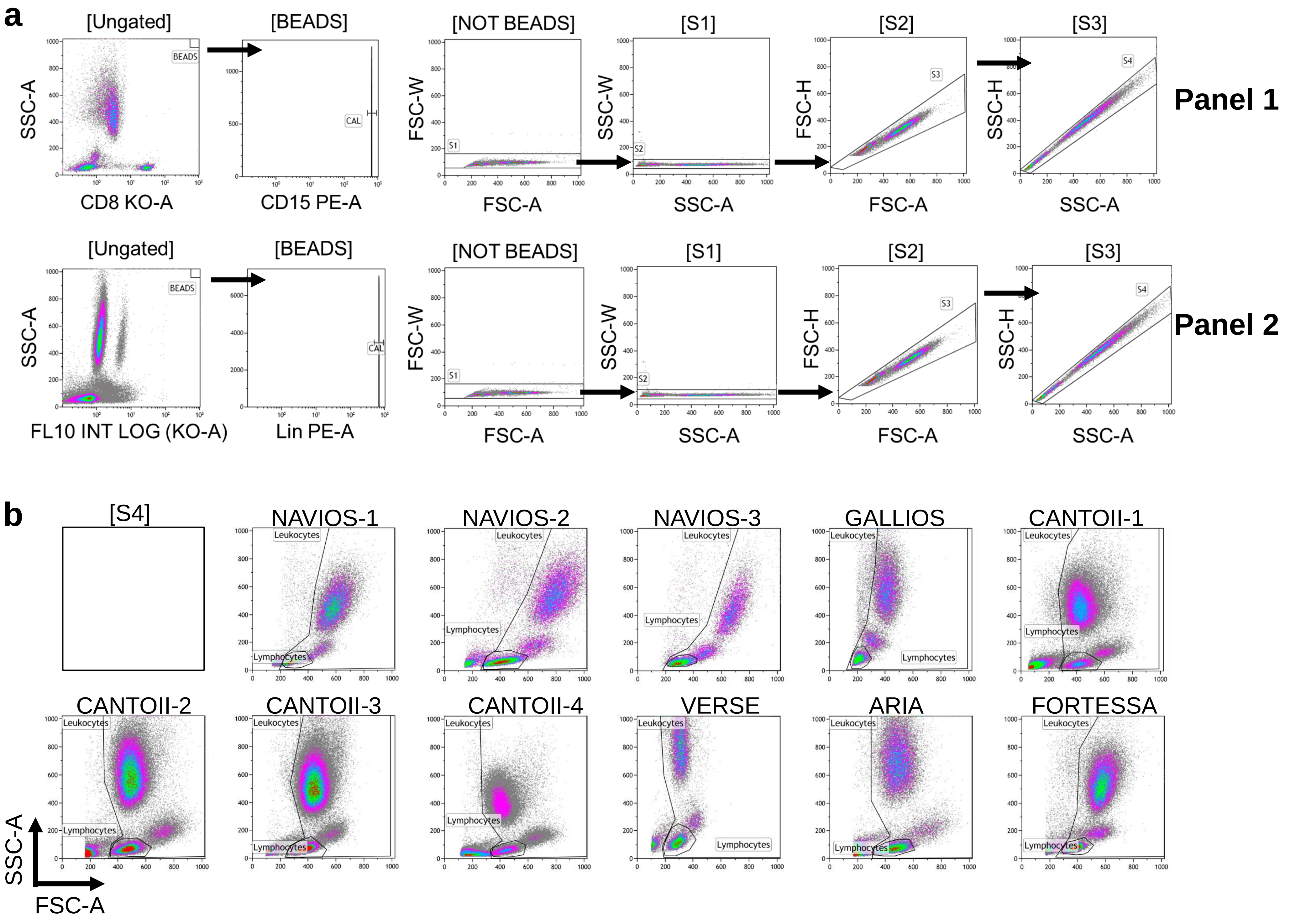
a
[Ungated]
[BEADS]
[NOT BEADS]
[S1]
[S2]
[S3]
Panel 1
SSC-A
CD8 KO-A
CD15 PE-A
FSC-W
FSC-A
SSC-W
SSC-A
FSC-H
SSC-H
BEADS
CAL
S1
S2
S3
S4
Panel 2
FL10 INT LOG (KO-A)
Lin PE-A
b
[S4]
NAVIOS-1
NAVIOS-2
NAVIOS-3
GALLIOS
CANTOII-1
CANTOII-2
CANTOII-3
CANTOII-4
VERSE
ARIA
FORTESSA
Leukocytes
Lymphocytes
SSC-A
FSC-A

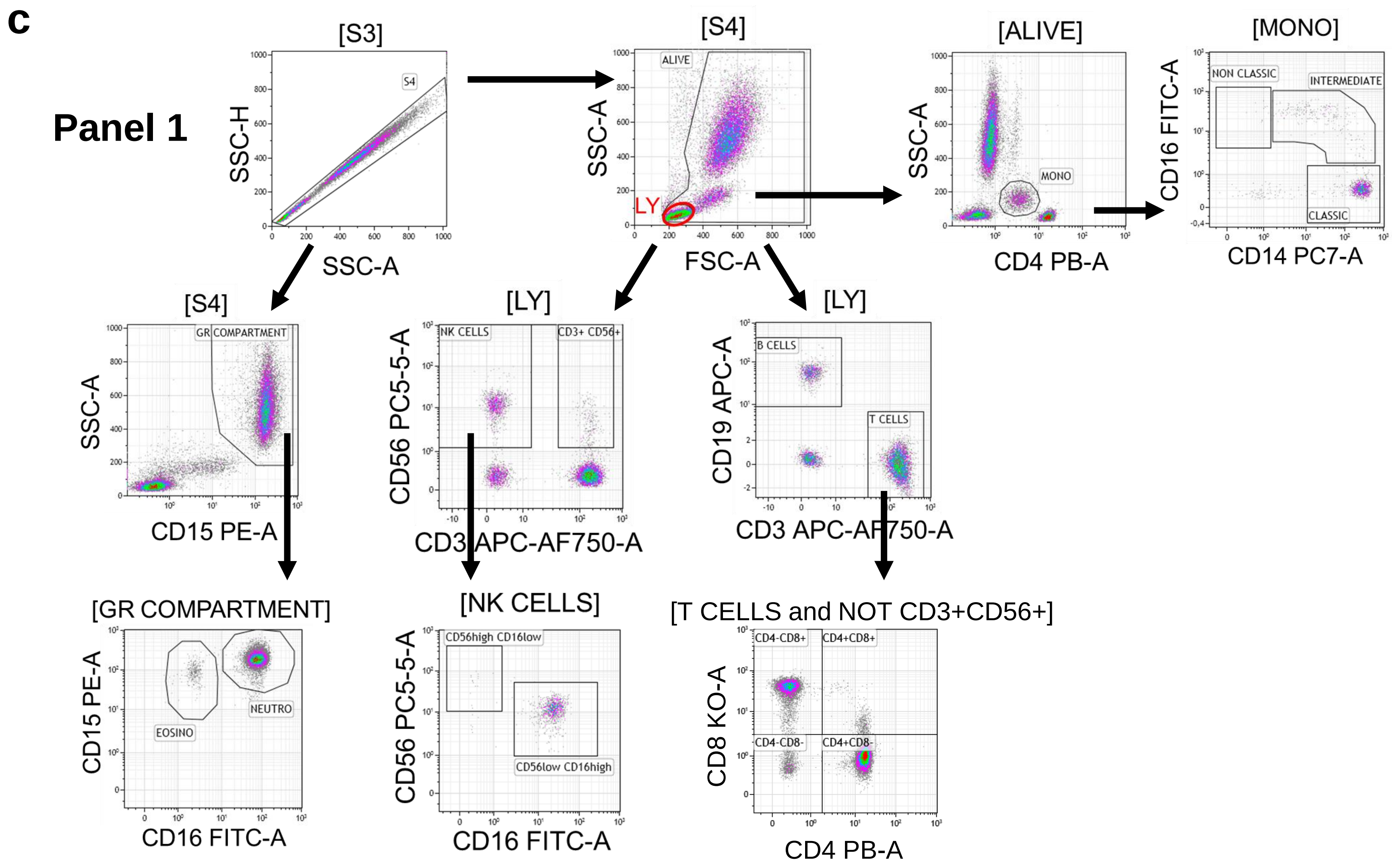
c
Panel 1
[S3]
SSC-H
SSC-A
S4
[S4]
SSC-A
FSC-A
ALIVE
LY
[ALIVE]
SSC-A
CD4 PB-A
MONO
[MONO]
CD16 FITC-A
CD14 PC7-A
NON CLASSIC
INTERMEDIATE
CLASSIC
[S4]
SSC-A
CD15 PE-A
GR COMPARTMENT
[LY]
CD56 PC5-5-A
CD3 APC-AF750-A
NK CELLS
CD3+ CD56+
[LY]
CD19 APC-A
CD3 APC-AF750-A
B CELLS
T CELLS
[GR COMPARTMENT]
CD15 PE-A
CD16 FITC-A
EOSINO
NEUTRO
[NK CELLS]
CD56 PC5-5-A
CD16 FITC-A
CD56high CD16low
CD56low CD16high
[T CELLS and NOT CD3+CD56+]
CD8 KO-A
CD4 PB-A
CD4-CD8+
CD4+CD8+
CD4-CD8-
CD4+CD8-

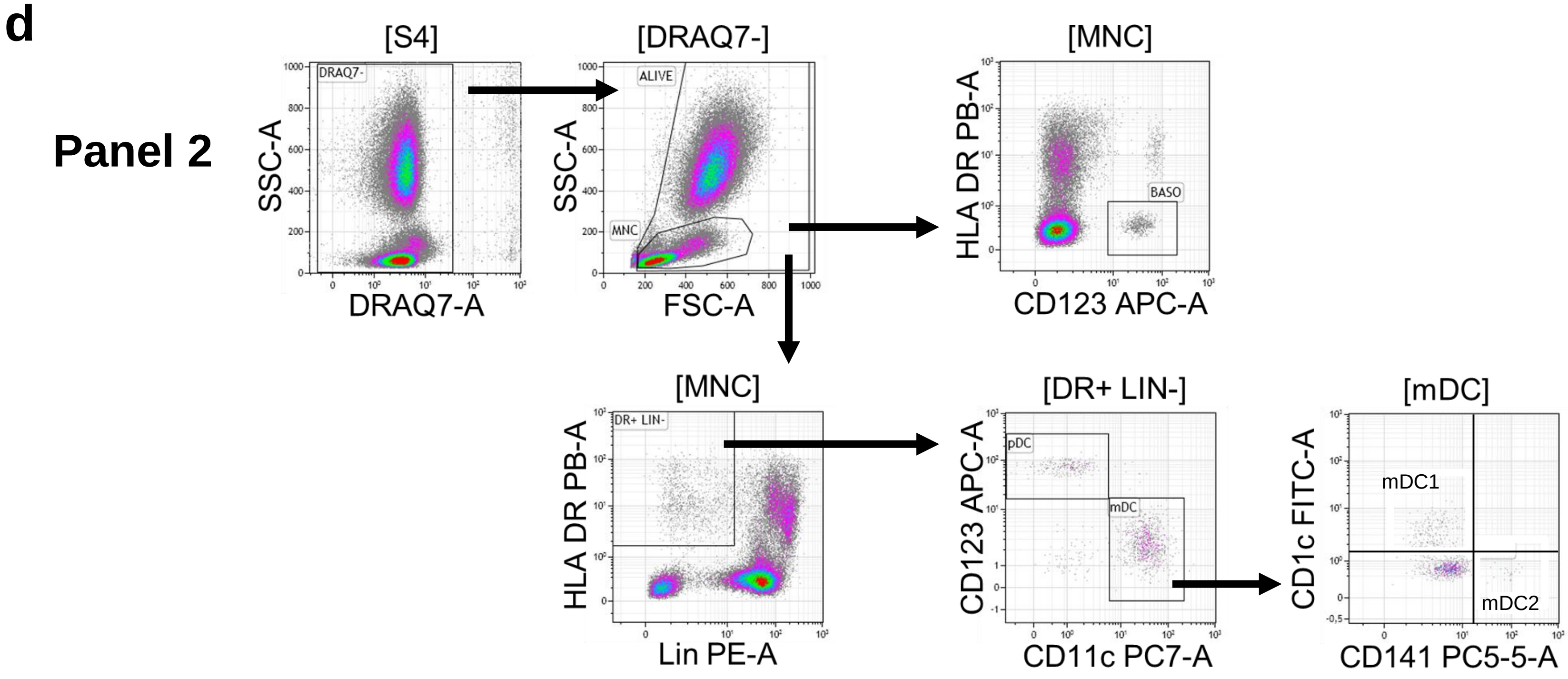
d
Panel 2
[S4]
DRAQ7-
SSC-A
DRAQ7-A
[DRAQ7-]
ALIVE
MNC
SSC-A
FSC-A
[MNC]
BASO
HLA DR PB-A
CD123 APC-A
[MNC]
DR+ LIN-
HLA DR PB-A
Lin PE-A
[DR+ LIN-]
pDC
mDC
CD123 APC-A
CD11c PC7-A
[mDC]
mDC1
mDC2
CD1c FITC-A
CD141 PC5-5-A
