# Supplementary material for: Standardization procedure for flow cytometry data harmonization in prospective multicenter studies
Source: Sci Rep. 2020 Jul 14;10:11567. doi: 10.1038/s41598-020-68468-3 (PMC7360585; doi:10.1038/s41598-020-68468-3)
Supplement: Supplementary file 7 — Supplementary Figure 4. [file 41598_2020_68468_MOESM7_ESM.pdf]

Standardization procedure for flow cytometry data harmonization in prospective multicenter studies

Lucas Le Lann[1], PRECISESADS Flow Cytometry Study Group[1] and PRECISESADS Clinical Consortium[1], Pierre-Emmanuel Jouve[2], Marta Alarcón-Riquelme[3], Christophe Jamin[1,4], Jacques-Olivier Pers[1]

Supplementary Figure 4

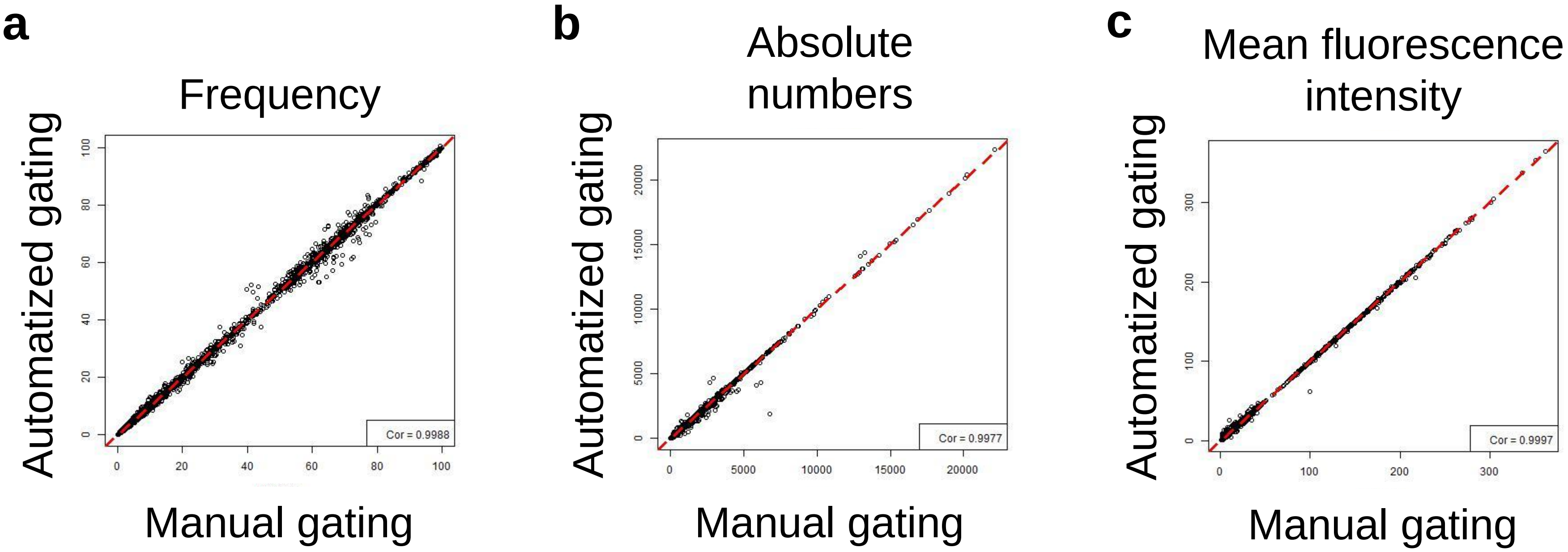
a
Frequency
Automatized gating
Manual gating
0
20
40
60
80
100
Cor = 0.9988
b
Absolute numbers
Automatized gating
Manual gating
0
5000
10000
15000
20000
Cor = 0.9977
c
Mean fluorescence intensity
Automatized gating
Manual gating
0
100
200
300
Cor = 0.9997
