# Supplementary material for: Standardization procedure for flow cytometry data harmonization in prospective multicenter studies
Source: Sci Rep. 2020 Jul 14;10:11567. doi: 10.1038/s41598-020-68468-3 (PMC7360585; doi:10.1038/s41598-020-68468-3)
Supplement: Supplementary file 8 — Supplementary Figure 5. [file 41598_2020_68468_MOESM8_ESM.pdf]

Standardization procedure for flow cytometry data harmonization in prospective multicenter studies

Lucas Le Lann[1], PRECISESADS Flow Cytometry Study Group[1] and PRECISESADS Clinical Consortium[1], Pierre-Emmanuel Jouve[2], Marta Alarcón-Riquelme[3], Christophe Jamin[1,4], Jacques-Olivier Pers[1]

Supplementary Figure 5

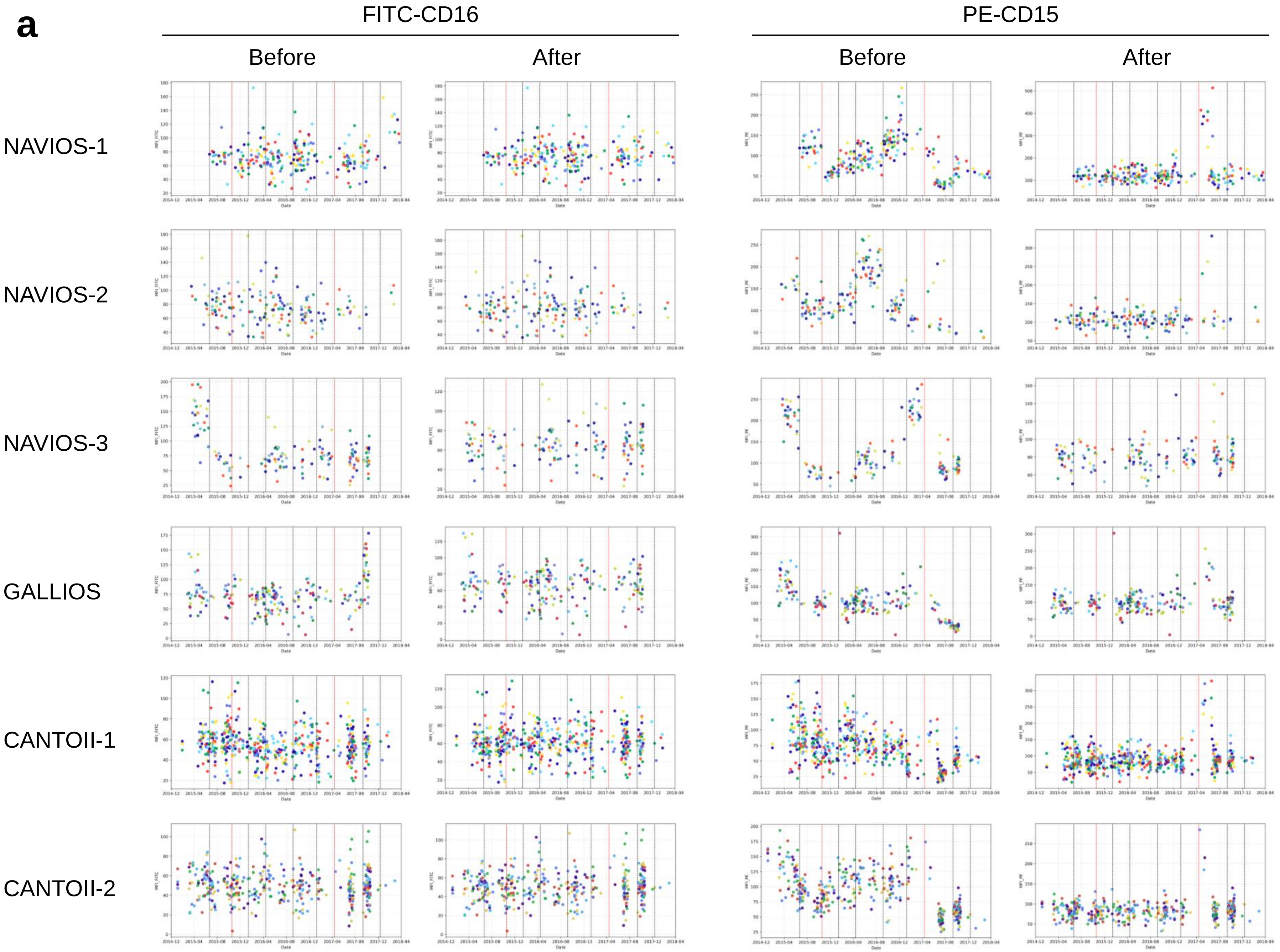
a
FITC-CD16
PE-CD15
Before
After
Before
After
NAVIOS-1
NAVIOS-2
NAVIOS-3
GALLIOS
CANTOII-1
CANTOII-2

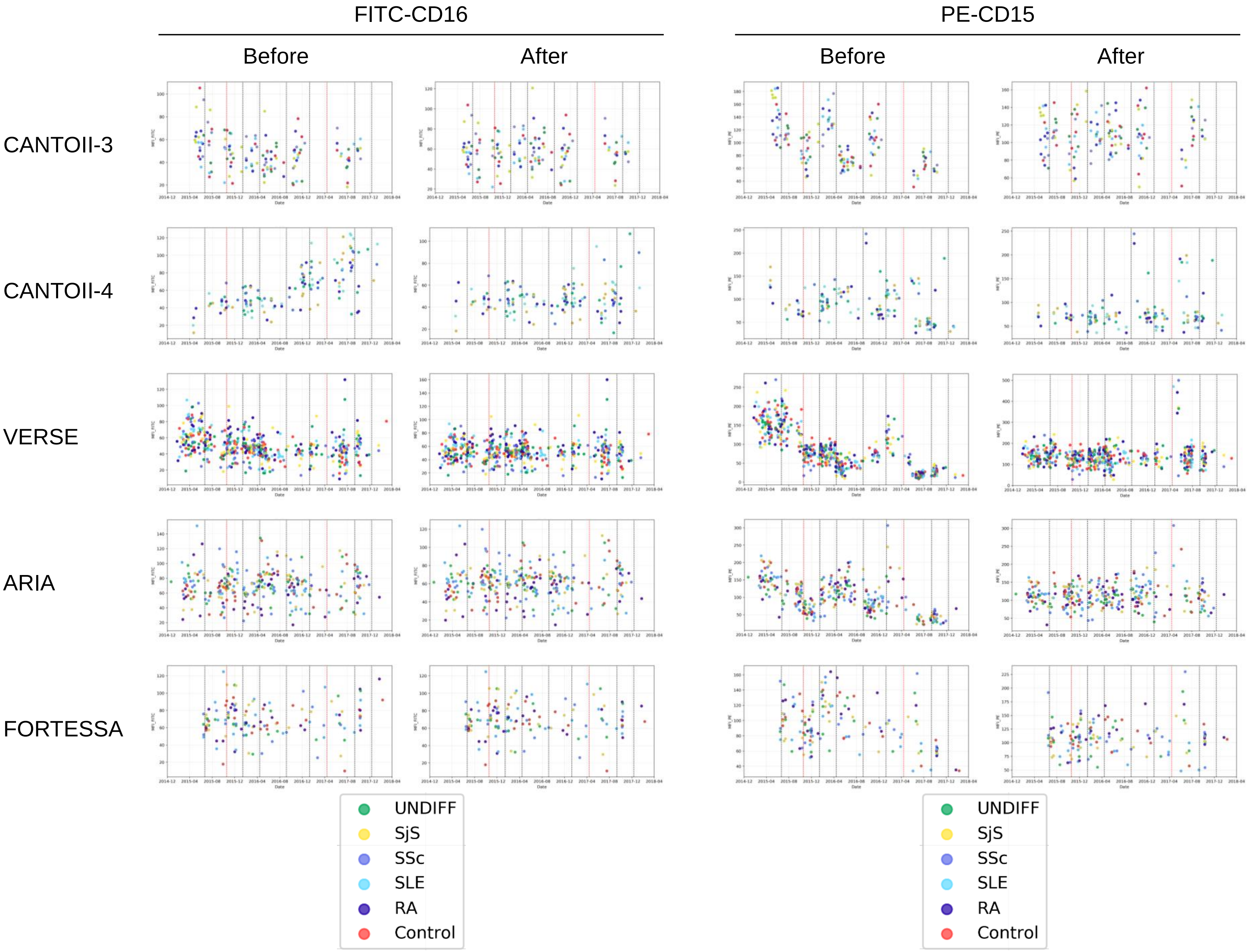
FITC-CD16
PE-CD15
Before
After
Before
After
CANTOII-3
CANTOII-4
VERSE
ARIA
FORTESSA
UNDIFF
SjS
SSc
SLE
RA
Control
UNDIFF
SjS
SSc
SLE
RA
Control

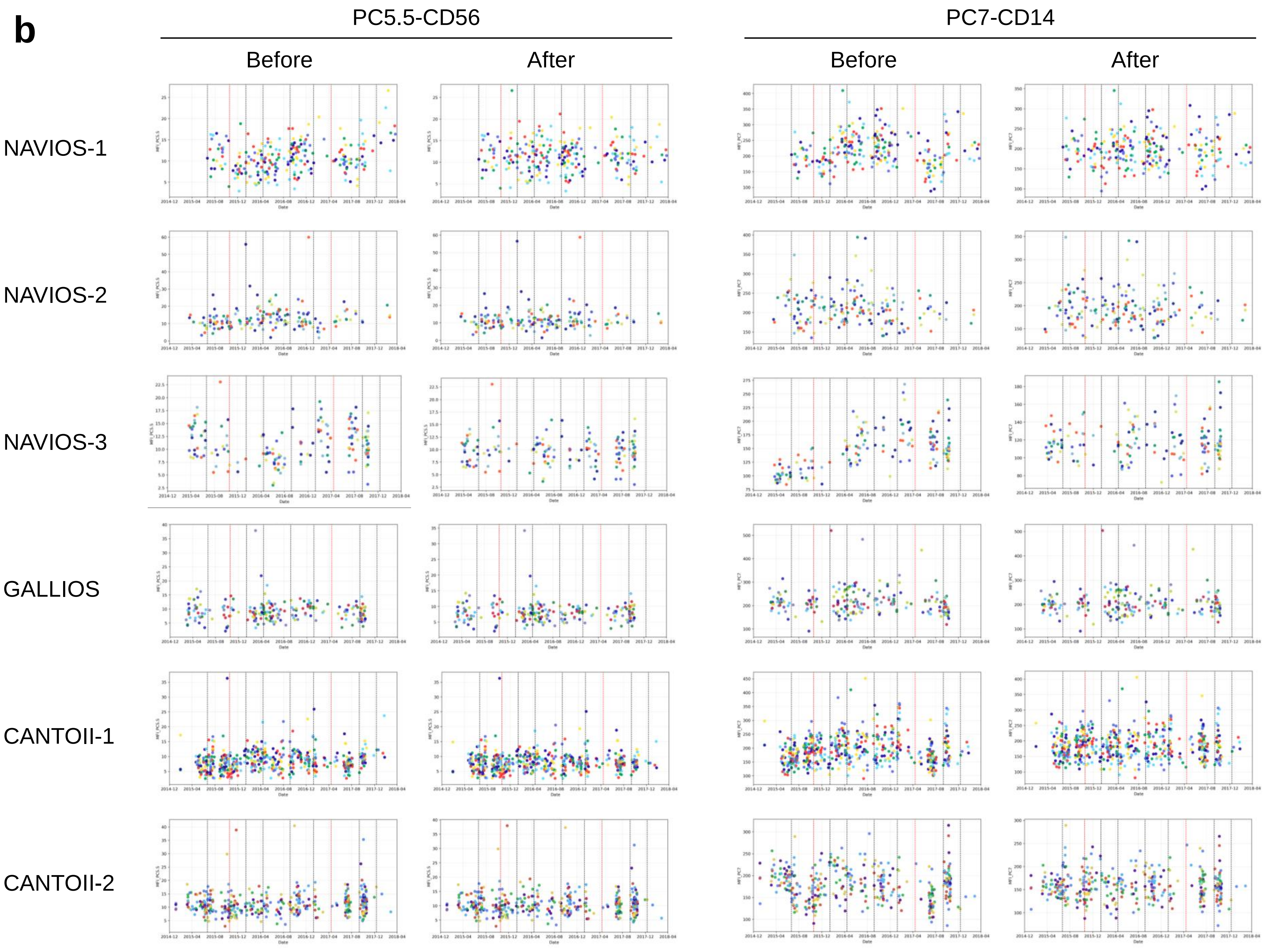
b
PC5.5-CD56
PC7-CD14
Before
After
Before
After
NAVIOS-1
NAVIOS-2
NAVIOS-3
GALLIOS
CANTOII-1
CANTOII-2
Date

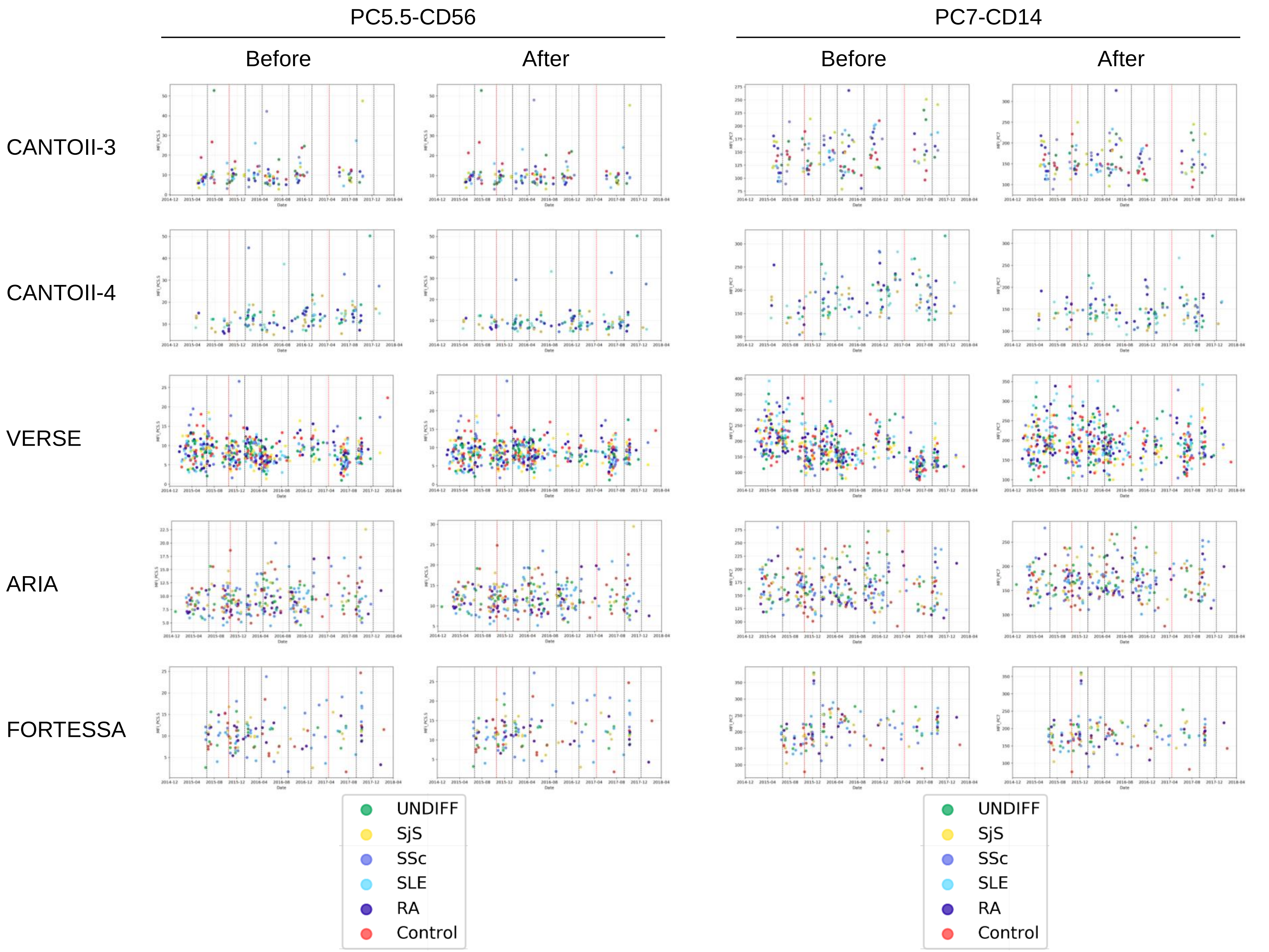
PC5.5-CD56
PC7-CD14
Before
After
Before
After
CANTOII-3
CANTOII-4
VERSE
ARIA
FORTESSA
UNDIFF
SjS
SSc
SLE
RA
Control

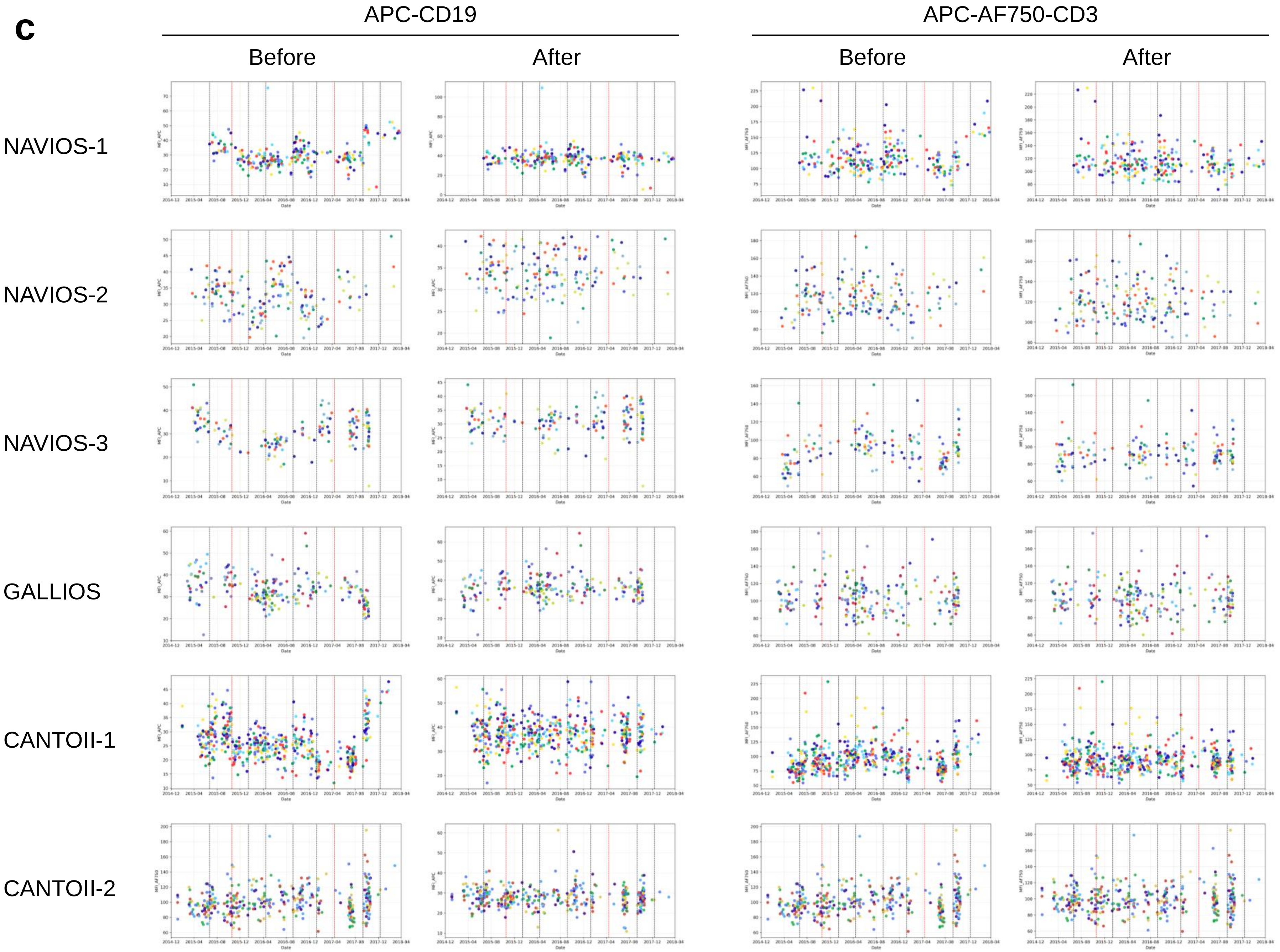
c
APC-CD19
APC-AF750-CD3
Before
After
Before
After
NAVIOS-1
NAVIOS-2
NAVIOS-3
GALLIOS
CANTOII-1
CANTOII-2

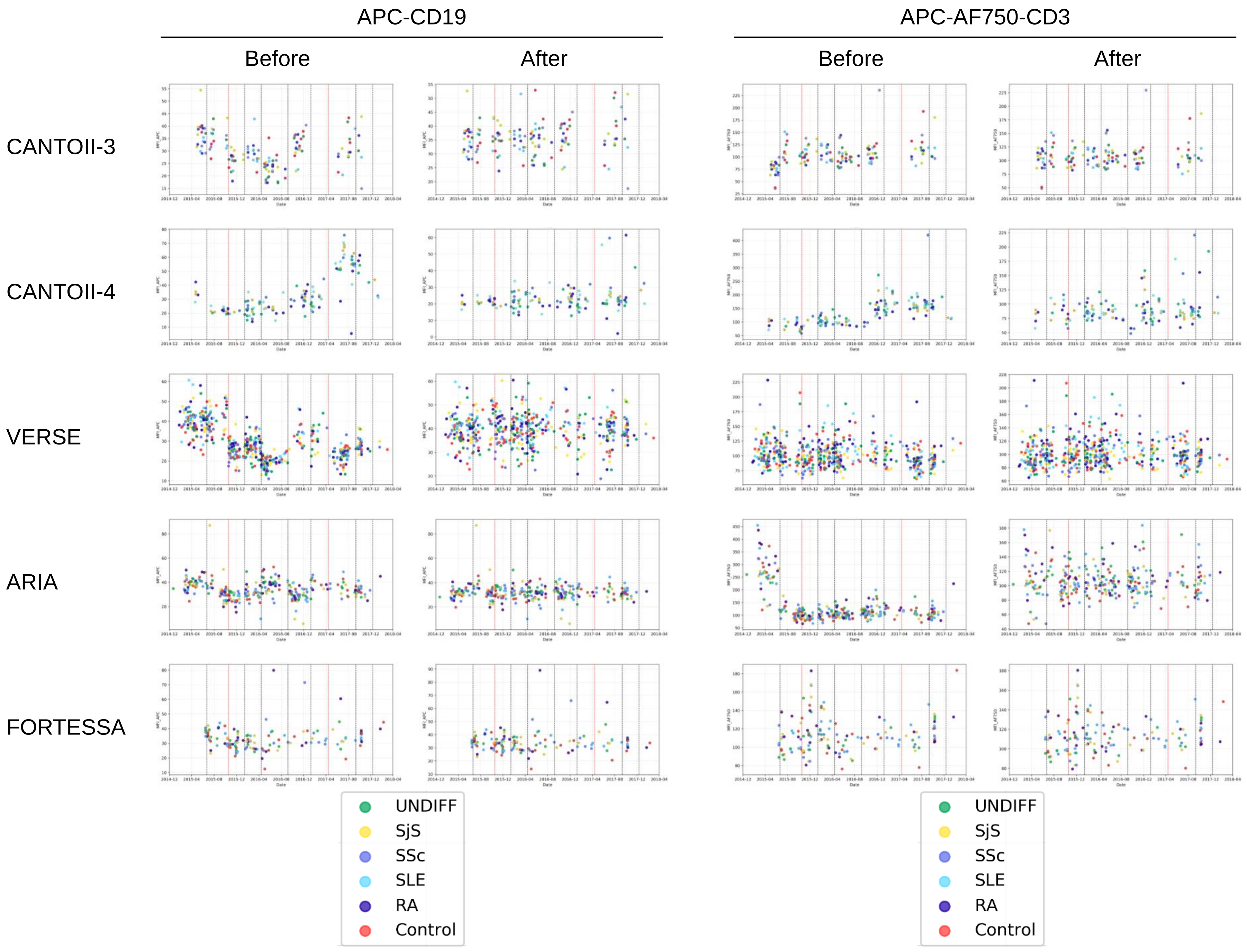
APC-CD19
APC-AF750-CD3
Before
After
Before
After
CANTOII-3
CANTOII-4
VERSE
ARIA
FORTESSA
UNDIFF
SjS
SSc
SLE
RA
Control

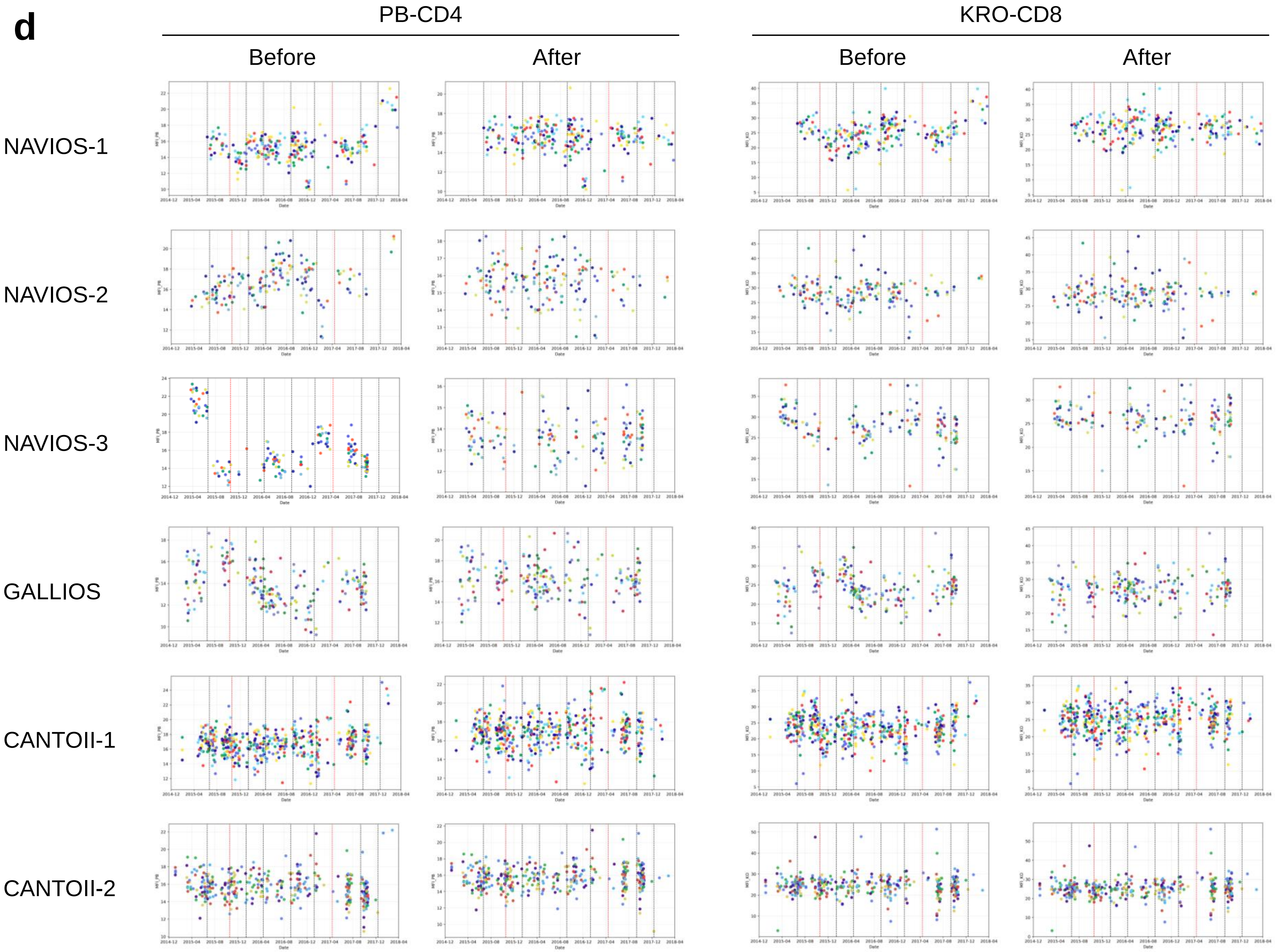
d
PB-CD4
KRO-CD8
Before
After
Before
After
NAVIOS-1
NAVIOS-2
NAVIOS-3
GALLIOS
CANTOII-1
CANTOII-2

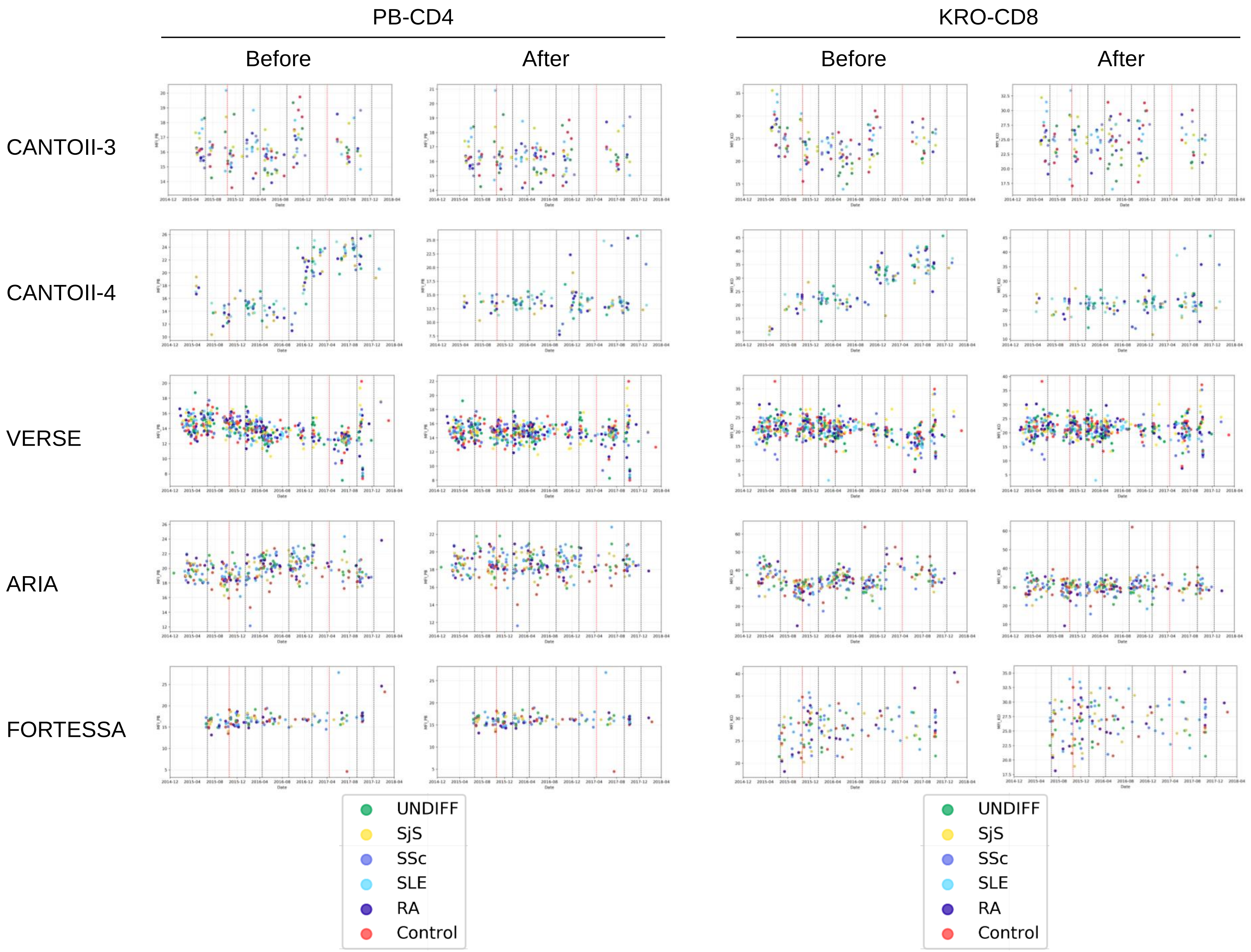
PB-CD4
KRO-CD8
Before
After
Before
After
CANTOII-3
CANTOII-4
VERSE
ARIA
FORTESSA
UNDIFF
SjS
SSc
SLE
RA
Control

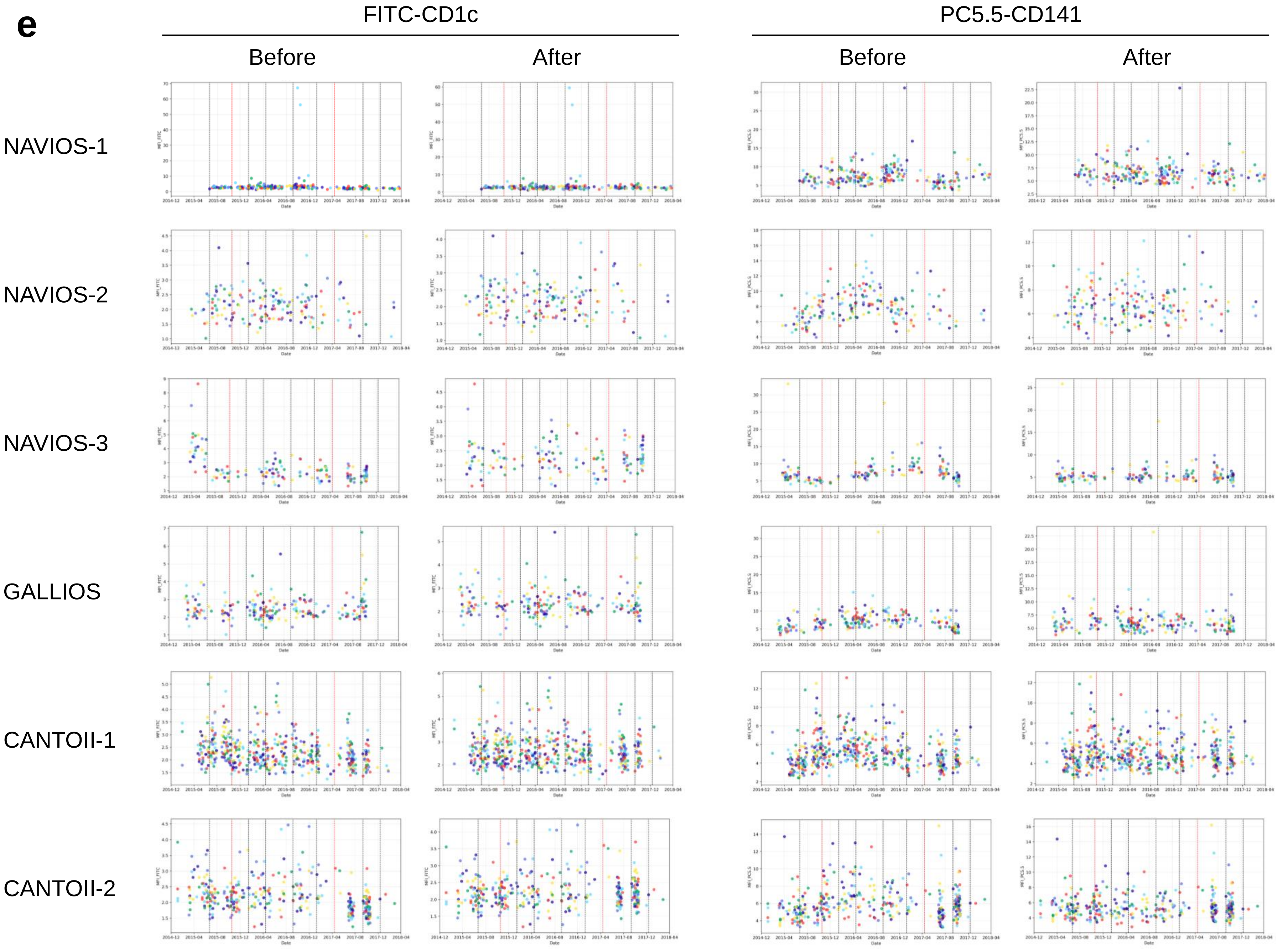
e
FITC-CD1c
PC5.5-CD141
Before
After
Before
After
NAVIOS-1
NAVIOS-2
NAVIOS-3
GALLIOS
CANTOII-1
CANTOII-2

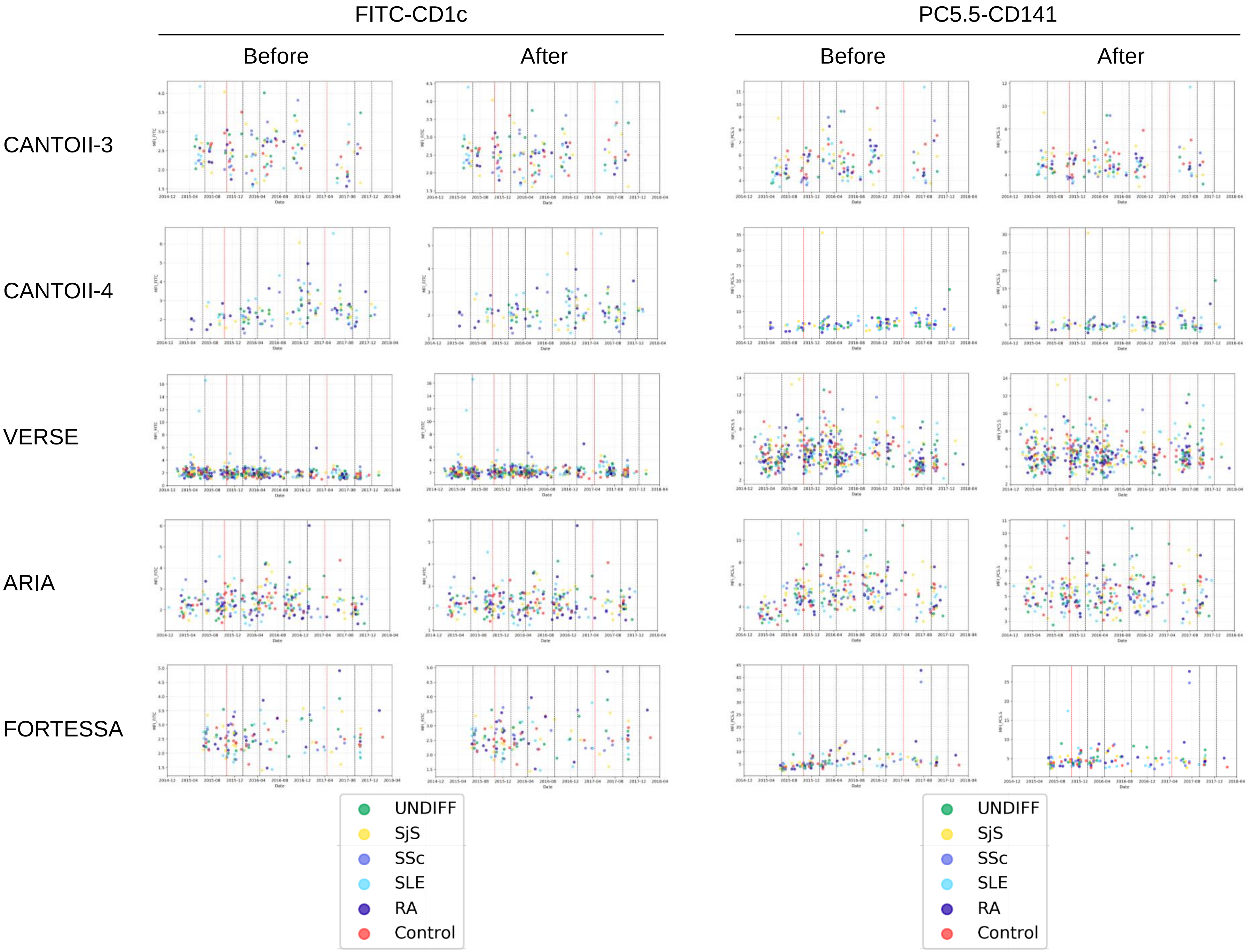
FITC-CD1c
PC5.5-CD141
Before
After
Before
After
CANTOII-3
CANTOII-4
VERSE
ARIA
FORTESSA
UNDIFF
SjS
SSc
SLE
RA
Control
UNDIFF
SjS
SSc
SLE
RA
Control

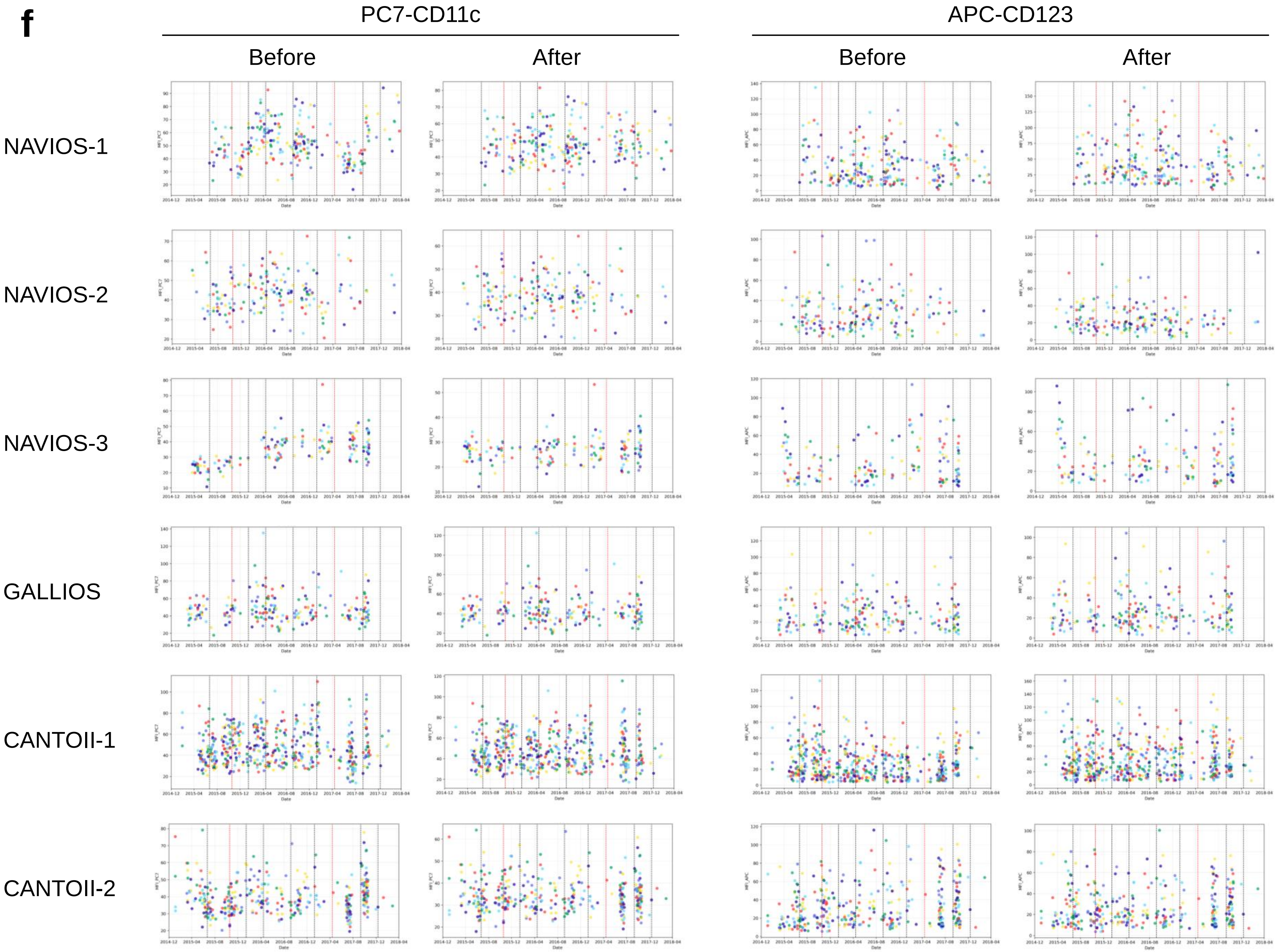
f
PC7-CD11c
APC-CD123
Before
After
Before
After
NAVIOS-1
NAVIOS-2
NAVIOS-3
GALLIOS
CANTOII-1
CANTOII-2

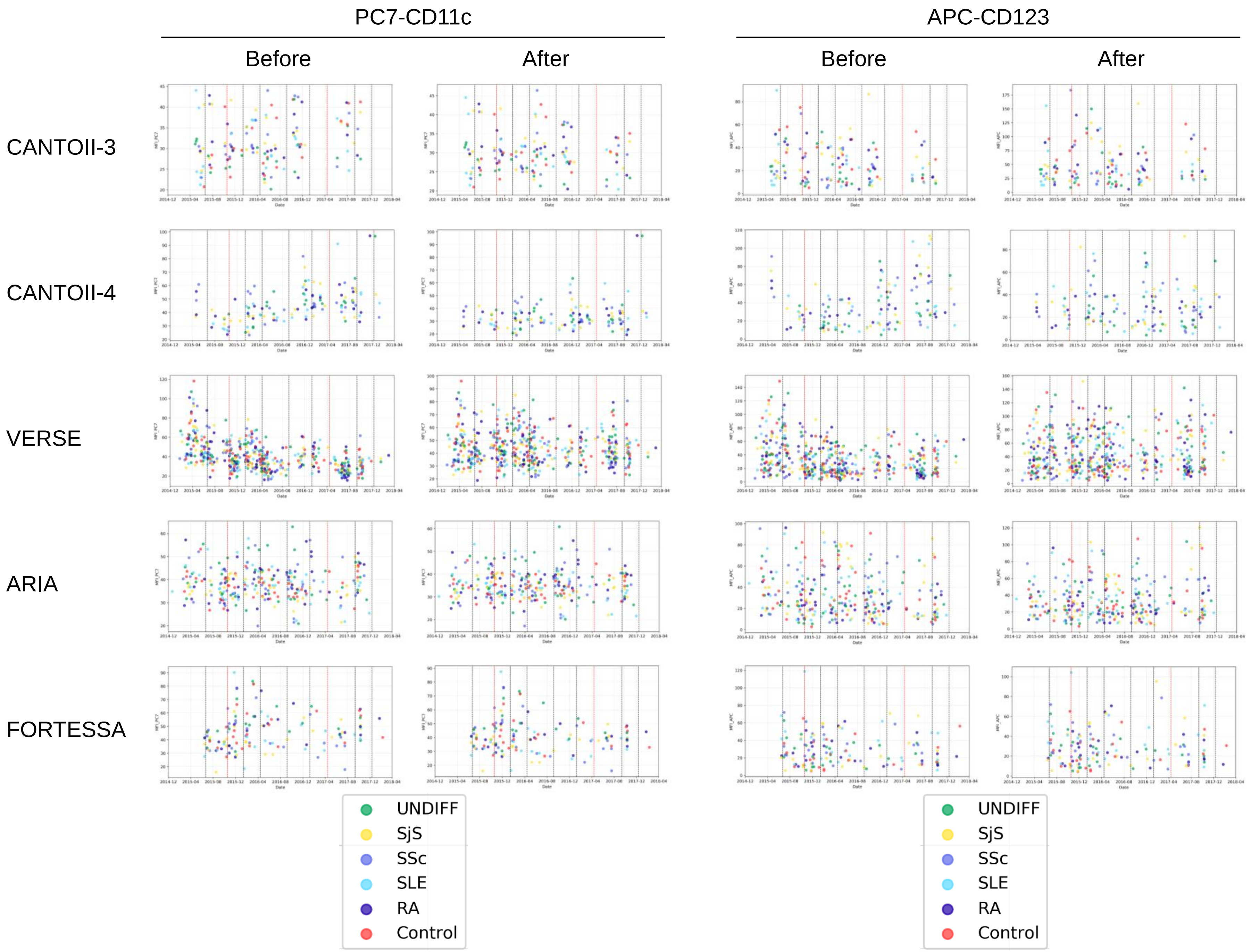
PC7-CD11c
APC-CD123
Before
After
Before
After
CANTOII-3
CANTOII-4
VERSE
ARIA
FORTESSA
UNDIFF
SjS
SSc
SLE
RA
Control
UNDIFF
SjS
SSc
SLE
RA
Control

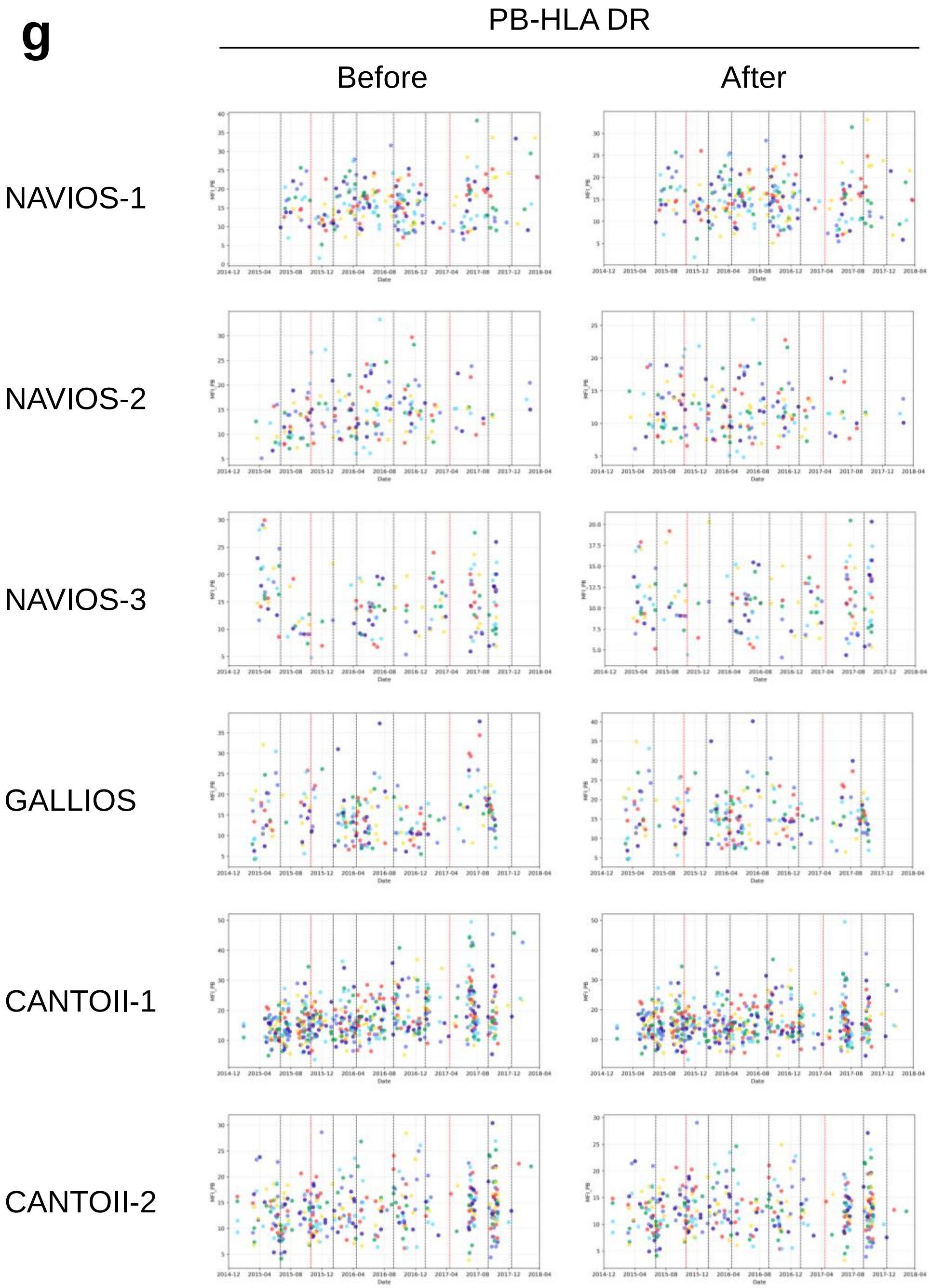
g
PB-HLA DR
Before
After
NAVIOS-1
NAVIOS-2
NAVIOS-3
GALLIOS
CANTOII-1
CANTOII-2

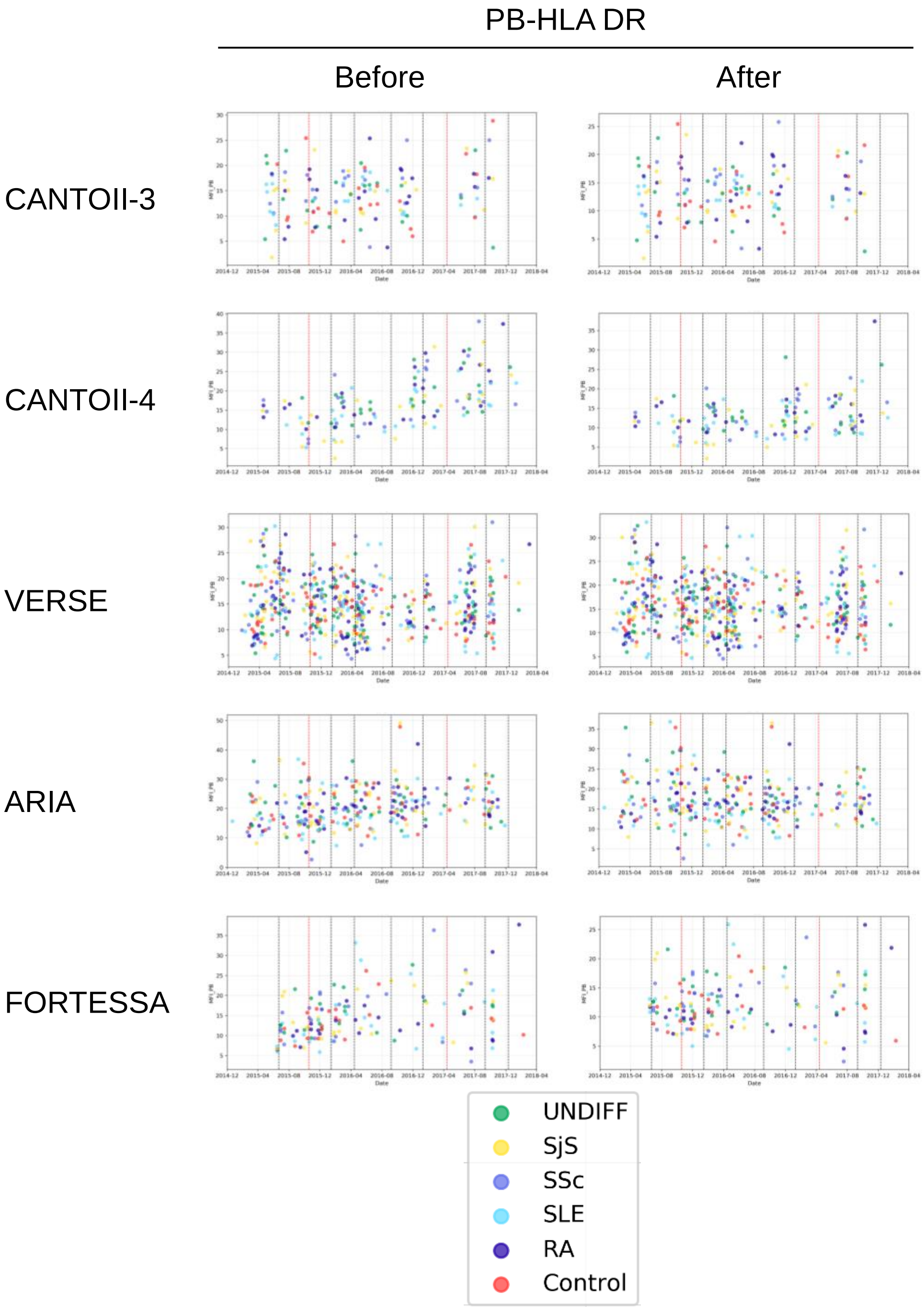
PB-HLA DR
Before
After
CANTOII-3
CANTOII-4
VERSE
ARIA
FORTESSA
UNDIFF
SjS
SSc
SLE
RA
Control
