# Supplementary material for: Standardization procedure for flow cytometry data harmonization in prospective multicenter studies
Source: Sci Rep. 2020 Jul 14;10:11567. doi: 10.1038/s41598-020-68468-3 (PMC7360585; doi:10.1038/s41598-020-68468-3)
Supplement: Supplementary file 9 — Supplementary Figure 6. [file 41598_2020_68468_MOESM9_ESM.pdf]

Standardization procedure for flow cytometry data harmonization in prospective multicenter studies

Lucas Le Lann[1], PRECISESADS Flow Cytometry Study Group[1] and PRECISESADS Clinical Consortium[1], Pierre-Emmanuel Jouve[2], Marta Alarcón-Riquelme[3], Christophe Jamin[1,4], Jacques-Olivier Pers[1]

Supplementary Figure 6

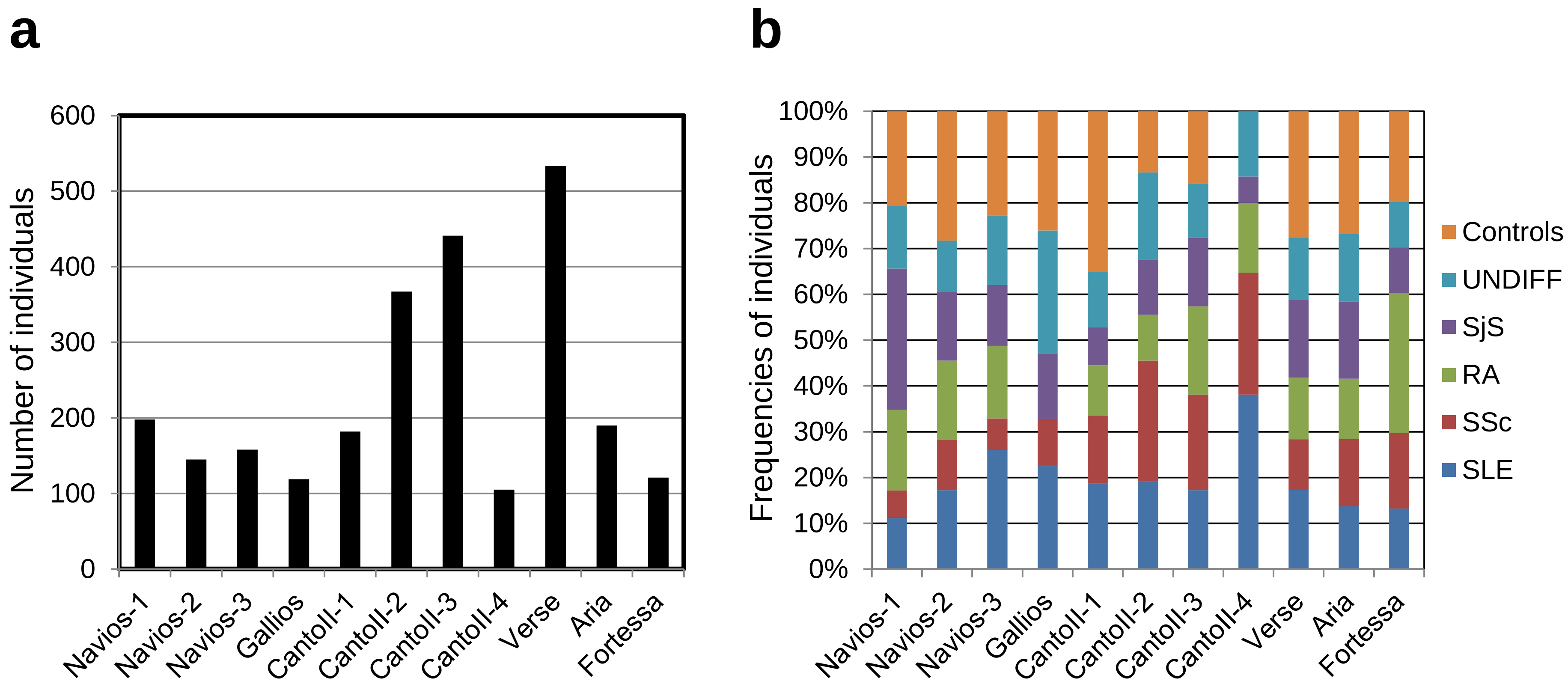
a
b
600
500
400
300
200
100
0
Number of individuals
Navios-1
Navios-2
Navios-3
Gallios
Cantoll-1
Cantoll-2
Cantoll-3
Cantoll-4
Verse
Aria
Fortessa
100%
90%
80%
70%
60%
50%
40%
30%
20%
10%
0%
Frequencies of individuals
Controls
UNDIFF
SjS
RA
SSc
SLE

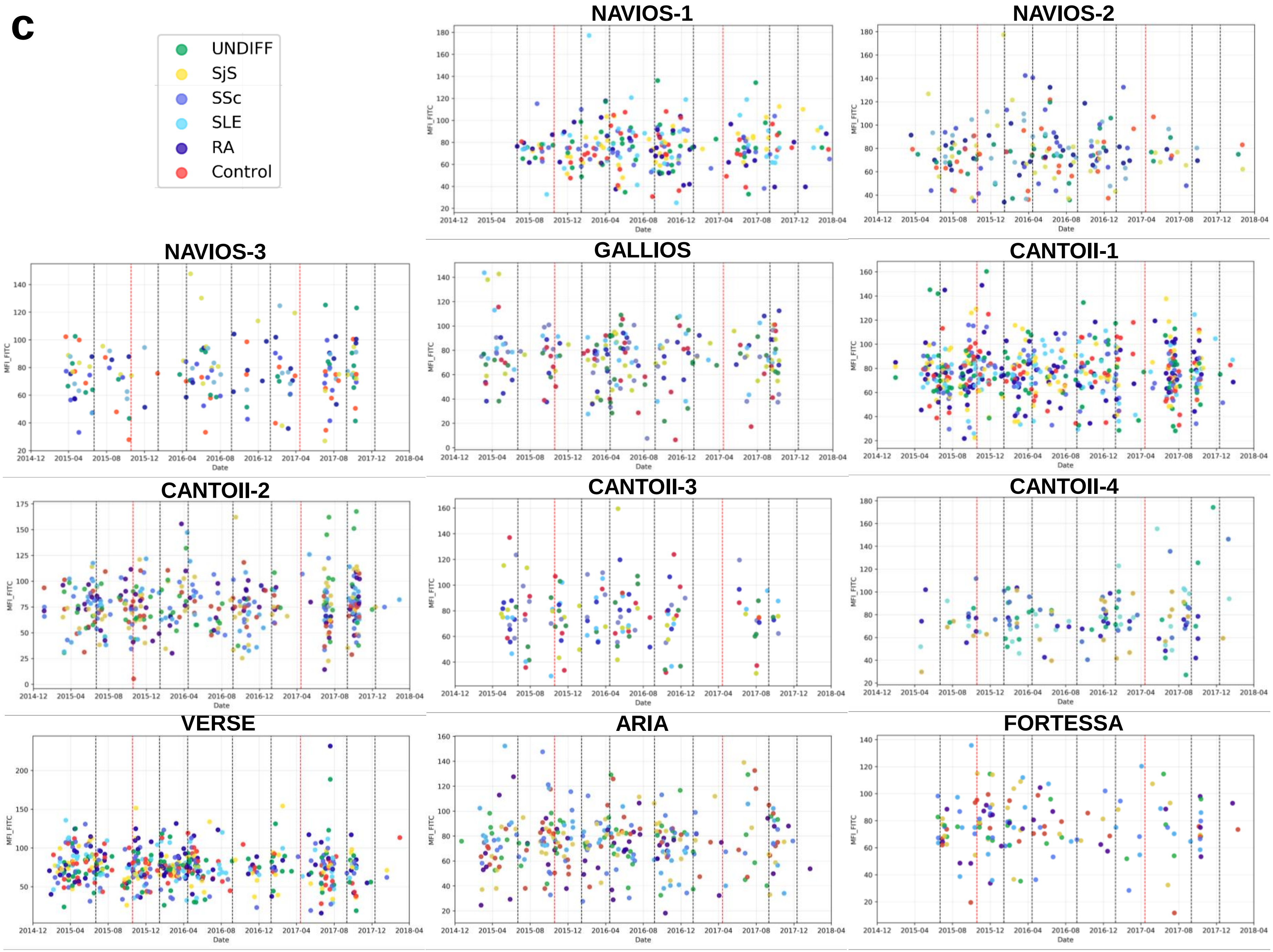
c
UNDIFF
SjS
SSc
SLE
RA
Control
NAVIOS-1
NAVIOS-2
NAVIOS-3
GALLIOS
CANTOII-1
CANTOII-2
CANTOII-3
CANTOII-4
VERSE
ARIA
FORTESSA
MFI_FITC
Date
