# Supplementary material for: Standardization procedure for flow cytometry data harmonization in prospective multicenter studies
Source: Sci Rep. 2020 Jul 14;10:11567. doi: 10.1038/s41598-020-68468-3 (PMC7360585; doi:10.1038/s41598-020-68468-3)
Supplement: Supplementary file 10 — Supplementary Figure 7. [file 41598_2020_68468_MOESM10_ESM.pdf]

Standardization procedure for flow cytometry data harmonization in prospective multicenter studies

Lucas Le Lann[1], PRECISESADS Flow Cytometry Study Group[1] and PRECISESADS Clinical Consortium[1], Pierre-Emmanuel Jouve[2], Marta Alarcón-Riquelme[3], Christophe Jamin[1,4], Jacques-Olivier Pers[1]

Supplementary Figure 7

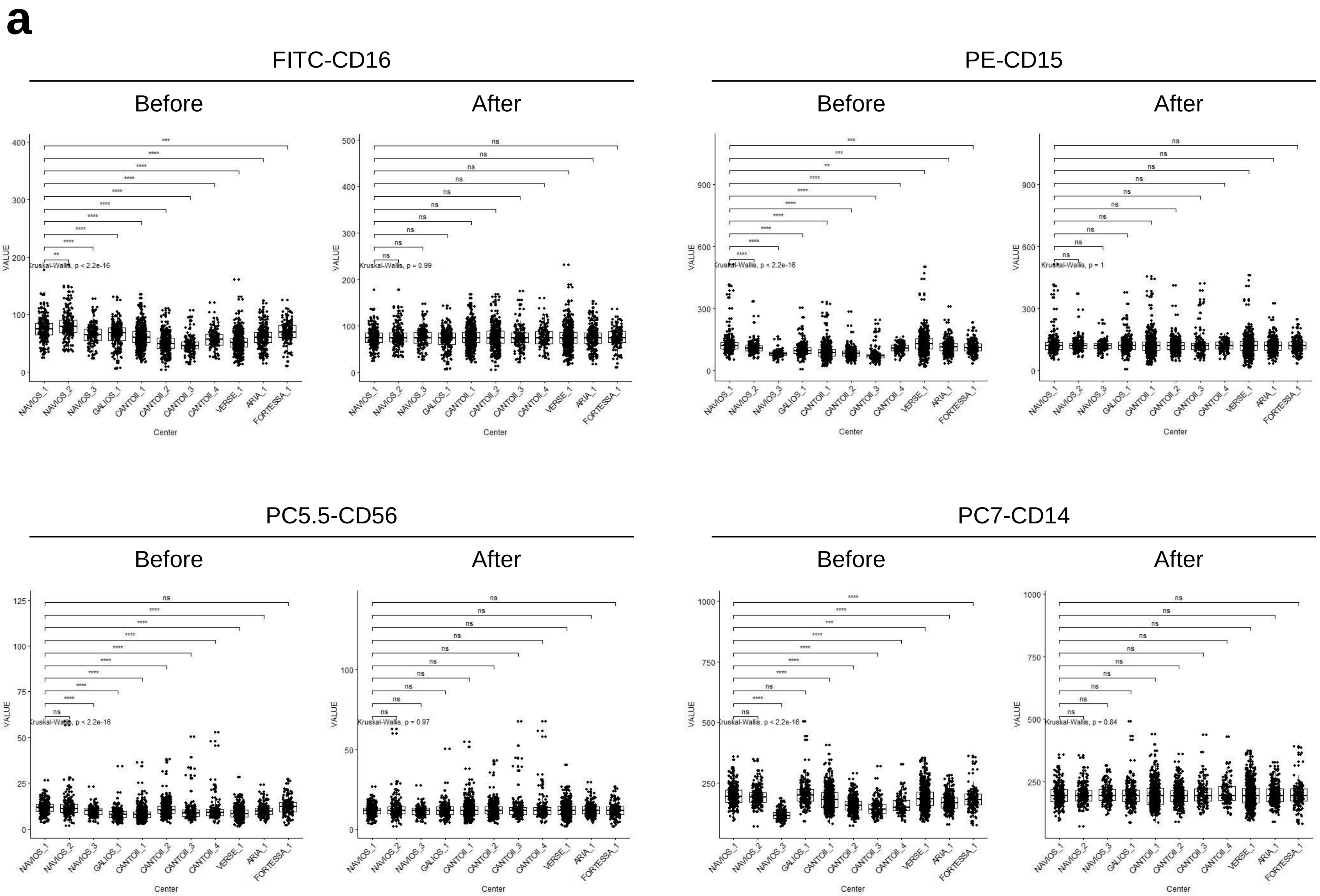
a
FITC-CD16
PE-CD15
PC5.5-CD56
PC7-CD14
Before
After
VALUE
Center
Kruskal-Wallis, p < 2.2e-16
Kruskal-Wallis, p = 0.99
Kruskal-Wallis, p = 1
Kruskal-Wallis, p = 0.97
Kruskal-Wallis, p = 0.84
NAVIOS_1
NAVIOS_2
NAVIOS_3
GALIOS_1
CANTOII_1
CANTOII_2
CANTOII_3
CANTOII_4
VERSE_1
ARIA_1
FORTESSA_1

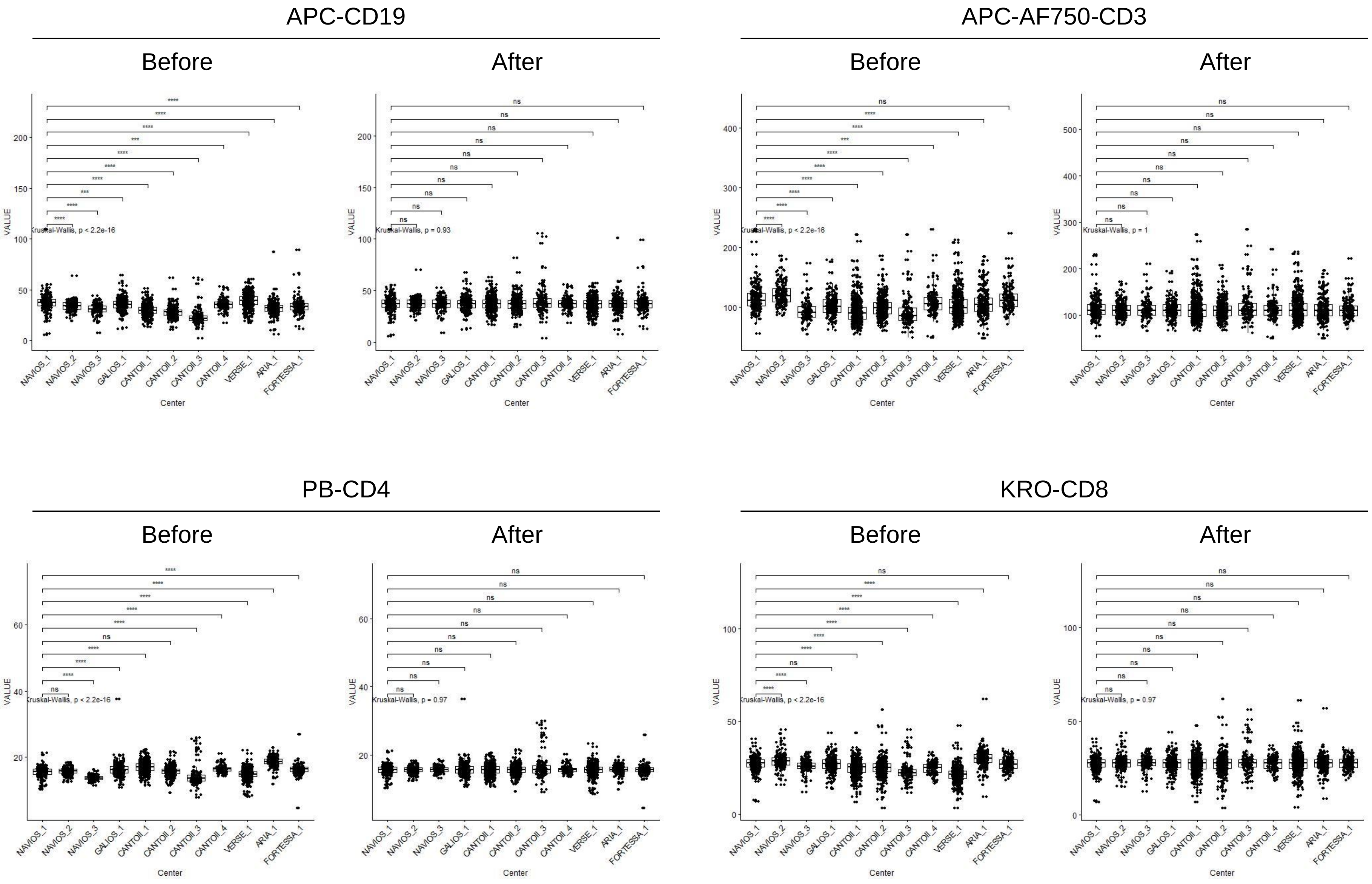
APC-CD19
Before
After
Kruskal-Wallis, p < 2.2e-16
Kruskal-Wallis, p = 0.93
APC-AF750-CD3
Before
After
Kruskal-Wallis, p < 2.2e-16
Kruskal-Wallis, p = 1
PB-CD4
Before
After
Kruskal-Wallis, p < 2.2e-16
Kruskal-Wallis, p = 0.97
KRO-CD8
Before
After
Kruskal-Wallis, p < 2.2e-16
Kruskal-Wallis, p = 0.97
VALUE
Center
NAVIOS_1
NAVIOS_2
NAVIOS_3
GALIOS_1
CANTOII_1
CANTOII_2
CANTOII_3
CANTOII_4
VERSE_1
ARIA_1
FORTESSA_1

b

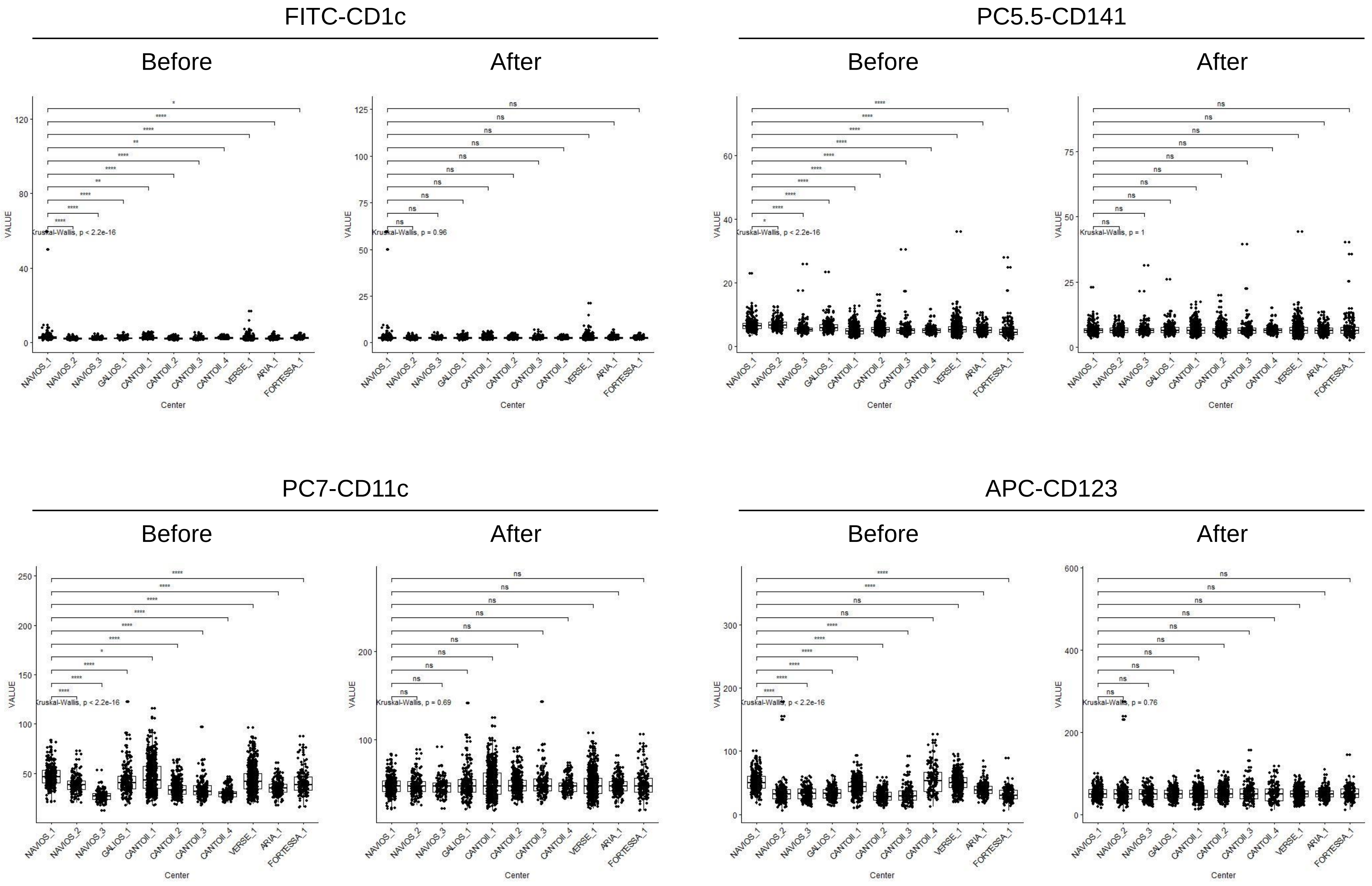
FITC-CD1c
Before
After
Kruskal-Wallis, p < 2.2e-16
Kruskal-Wallis, p = 0.96
PC5.5-CD141
Before
After
Kruskal-Wallis, p < 2.2e-16
Kruskal-Wallis, p = 1
PC7-CD11c
Before
After
Kruskal-Wallis, p < 2.2e-16
Kruskal-Wallis, p = 0.69
APC-CD123
Before
After
Kruskal-Wallis, p < 2.2e-16
Kruskal-Wallis, p = 0.76
VALUE
Center
NAVIOS_1
NAVIOS_2
NAVIOS_3
GALIOS_1
CANTOII_1
CANTOII_2
CANTOII_3
CANTOII_4
VERSE_1
ARIA_1
FORTESSA_1

PB-HLA DR

Before After

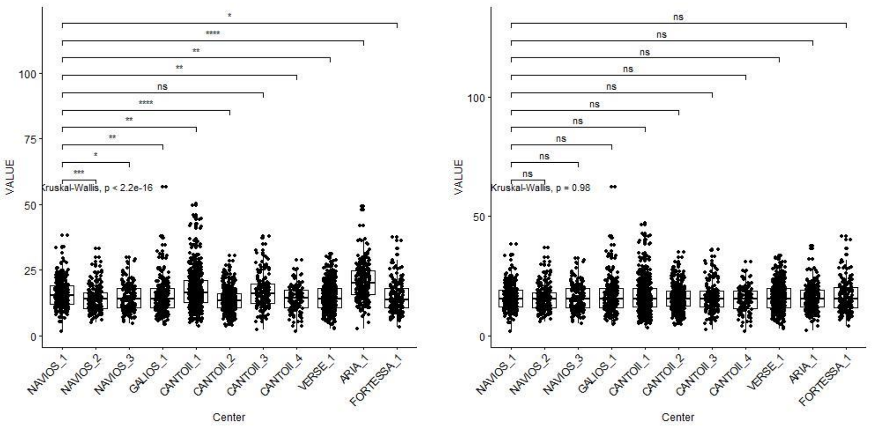

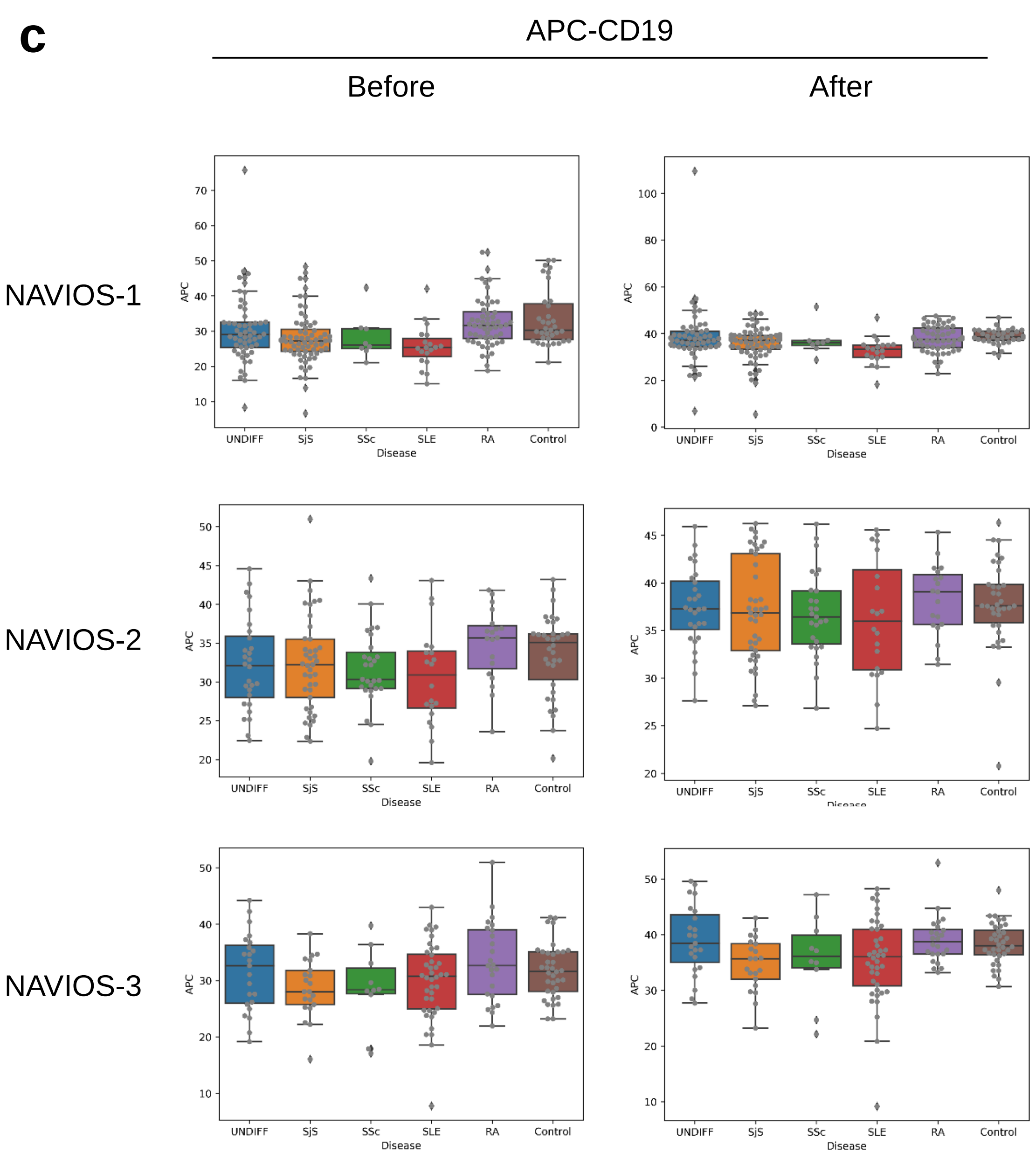

c
APC-CD19
Before
After
NAVIOS-1
NAVIOS-2
NAVIOS-3
APC
Disease
UNDIFF
SjS
SSc
SLE
RA
Control

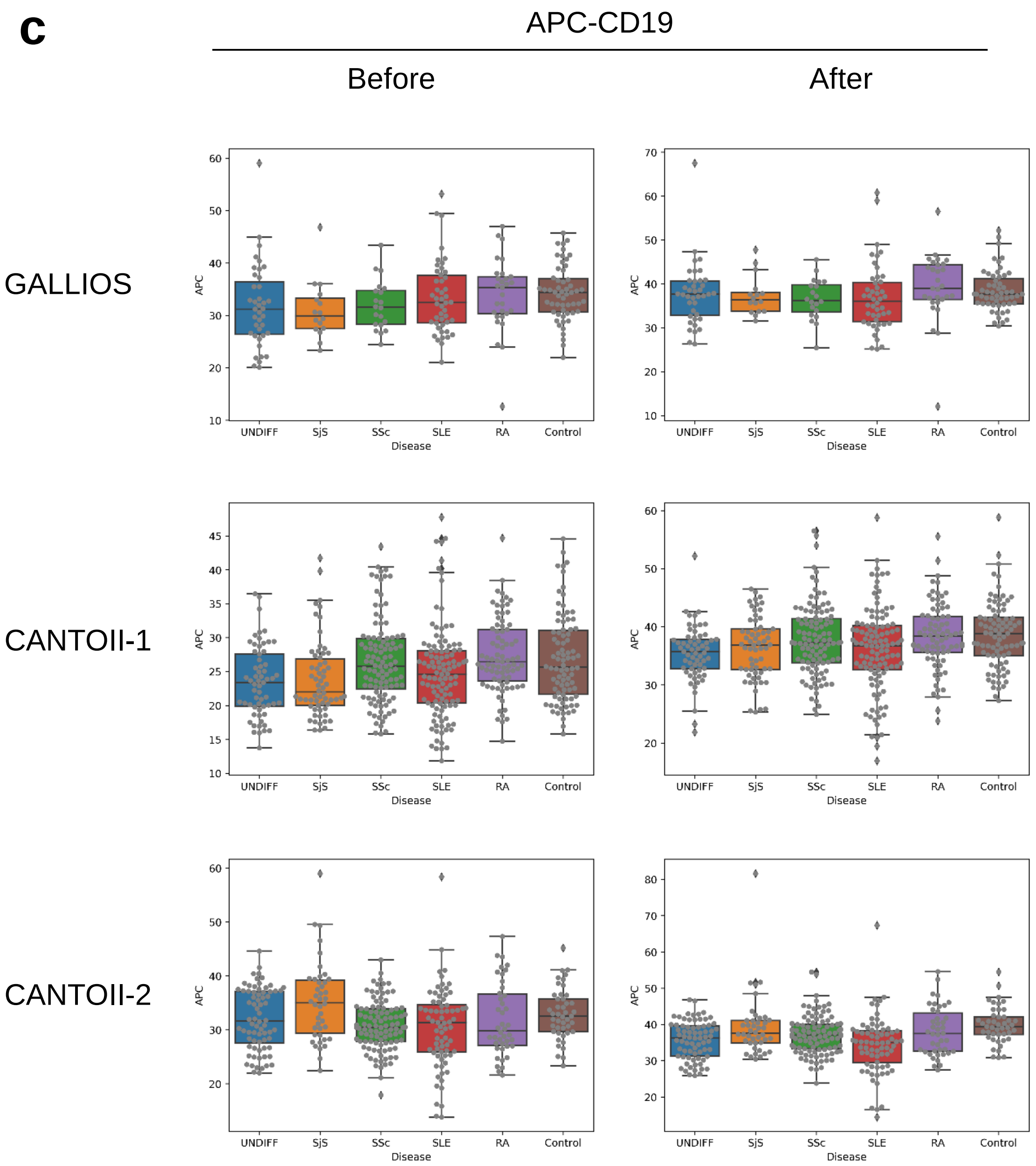
c
APC-CD19
Before
After
GALLIOS
CANTOII-1
CANTOII-2
APC
UNDIFF
SjS
SSc
SLE
RA
Control
Disease

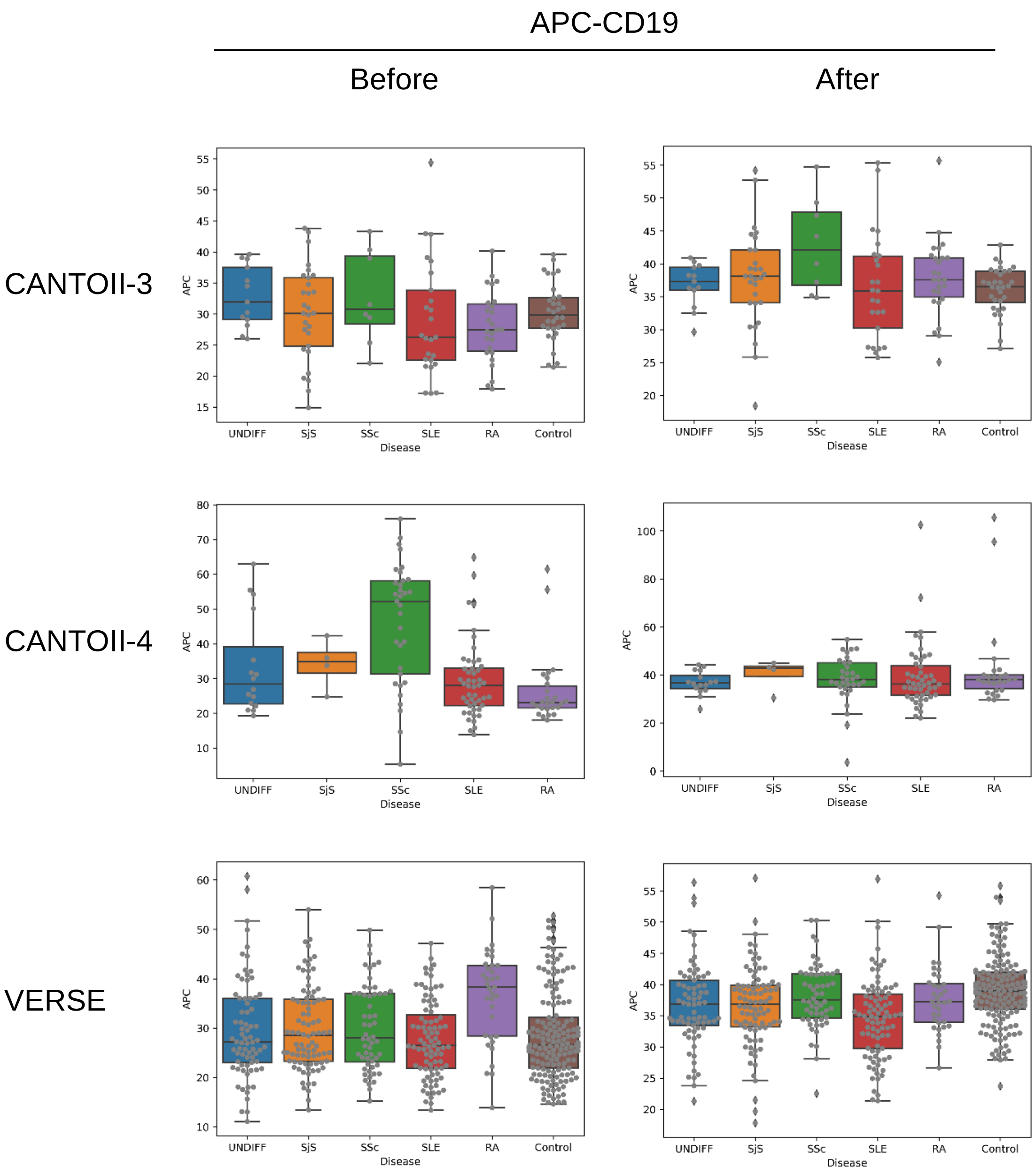

APC-CD19
Before
After
CANTOII-3
CANTOII-4
VERSE
APC
Disease
UNDIFF
SjS
SSc
SLE
RA
Control

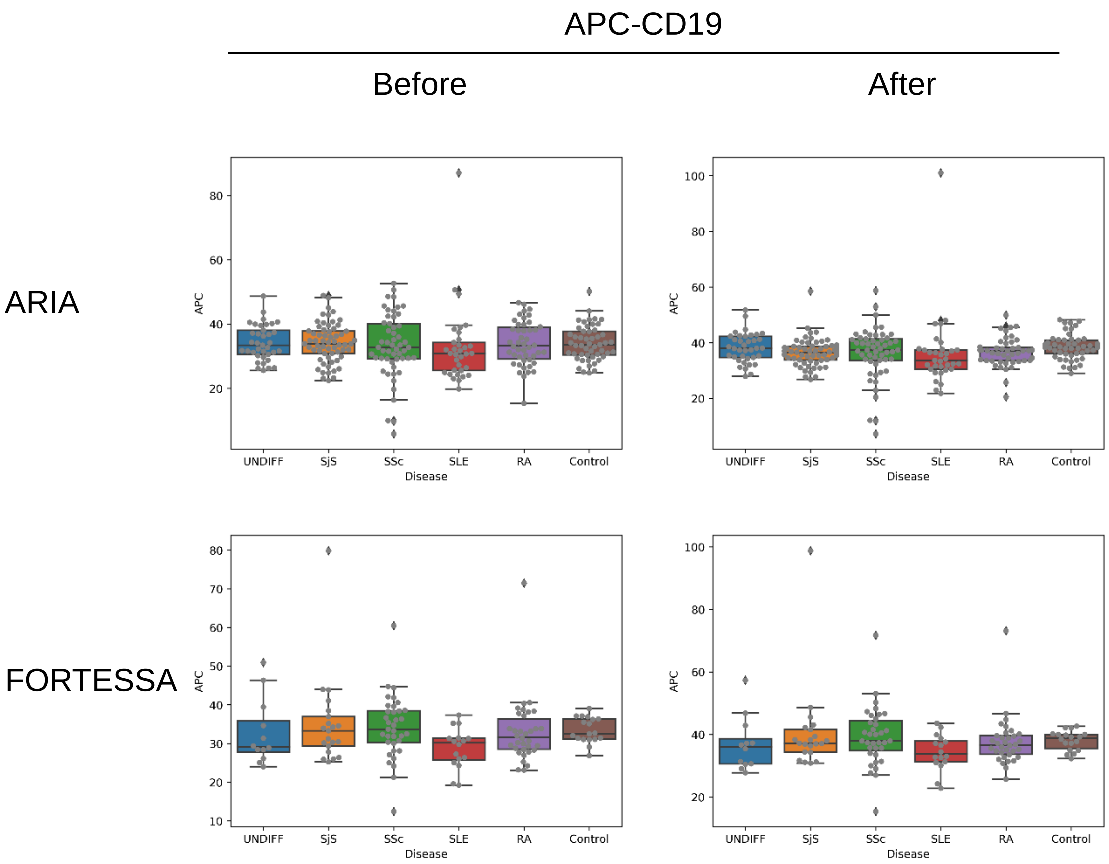
APC-CD19
Before
After
ARIA
FORTESSA
APC
Disease
UNDIFF
SjS
SSc
SLE
RA
Control
